# Transcriptome of the Female Synganglion of the Black-Legged Tick *Ixodes scapularis* (Acari: Ixodidae) with Comparison between Illumina and 454 Systems

**DOI:** 10.1371/journal.pone.0102667

**Published:** 2014-07-30

**Authors:** Noble Egekwu, Daniel E. Sonenshine, Brooke W. Bissinger, R. Michael Roe

**Affiliations:** 1 Department of Biological Sciences, Old Dominion University, Norfolk, Virginia, United States of America; 2 TyraTech, Inc., Morrisville, North Carolina, United States of America; 3 Department of Entomology, North Carolina State University, Raleigh, North Carolina, United States of America; Wake Forest University, United States of America

## Abstract

Illumina and 454 pyrosequencing were used to characterize genes from the synganglion of female *Ixodes scapularis*. GO term searching success for biological processes was similar for samples sequenced by both methods. However, for molecular processes, it was more successful for the Illumina samples than for 454 samples. Functional assignments of transcripts predicting neuropeptides, neuropeptide receptors, neurotransmitter receptors and other genes of interest was done, supported by strong e-values (<−6), and high consensus sequence alignments. Transcripts predicting 15 putative neuropeptide prepropeptides ((allatostatin, allatotropin, bursicon α, corticotropin releasing factor (CRF), CRF-binding protein, eclosion hormone, FMRFamide, glycoprotein A, insulin-like peptide, ion transport peptide, myoinhibitory peptide, inotocin ( =  neurophysin-oxytocin), Neuropeptide F, sulfakinin and SIFamide)) and transcripts predicting receptors for 14 neuropeptides (allatostatin, calcitonin, cardioacceleratory peptide, corazonin, CRF, eclosion hormone, gonadotropin-releasing hormone/AKH-like, insulin-like peptide, neuropeptide F, proctolin, pyrokinin, SIFamide, sulfakinin and tachykinin) are reported. Similar to *Dermacentor variabilis*, we found transcripts matching pro-protein convertase, essential for converting neuropeptide hormones to their mature form. Additionally, transcripts predicting 6 neurotransmitter/neuromodulator receptors (acetylcholine, GABA, dopamine, glutamate, octopamine and serotonin) and 3 neurotransmitter transporters (GABA transporter, noradrenalin-norepinephrine transporter and Na+-neurotransmitter/symporter) are described. Further, we found transcripts predicting genes for pheromone odorant receptor, gustatory receptor, novel GPCR messages, ecdysone nuclear receptor, JH esterase binding protein, steroidogenic activating protein, chitin synthase, chitinase, and other genes of interest. Also found were transcripts predicting genes for spermatogenesis-associated protein, major sperm protein, spermidine oxidase and spermidine synthase, genes not normally expressed in the female CNS of other invertebrates. The diversity of messages predicting important genes identified in this study offers a valuable resource useful for understanding how the tick synganglion regulates important physiological functions.

## Introduction

Ticks are obligate blood-feeding ectoparasites that serve as vectors of the causative agents of many important diseases affecting humans and animals, e.g., Lyme disease, Rocky Mountain spotted fever, tick-borne encephalitis, anaplasmosis, babesiosis and many others [1], [2]. The black-legged tick, *Ixodes scapularis*, is one of the most important vectors of infectious diseases to humans and animals throughout large areas of the United States and Canada [1], [3], [4]. *I. scapularis* is the primary vector of the microbial agents of Lyme disease, human granulocytic anaplasmosis, human babesiosis, relapsing fever and an encephalitis-causing agent (Powassan virus). Lyme disease is the most commonly reported vector-borne disease in the northern temperate zone regions of the northern hemisphere [5], [6] with 24,364 confirmed cases in the United States in 2011 [7]. Despite the many zoonotic diseases caused by tick-borne pathogens, control of ticks is still largely dependent on the use of chemical acaricides, with traditional acaricide targets being primarily neurologically-based [8]. The central nervous system (CNS) in ticks is composed of a single mass called the synganglion [9]. Despite the fact that most acaricides target the nervous system, relatively little is known about tick neurobiology and how blood-feeding and mating affect gene expression in the synganglion. Acaricide resistance has become widespread around the world causing a need for new acaricide targets or alternative methods of tick control [10]. Continued dependence on traditional chemical methods is becoming increasingly problematic in the face of ever greater demand for environmental protection and fear of chemical poisoning.

To search for alternatives to chemical control, a better understanding of the neurologic processes that control basic tick physiological processes is needed. Ixodid ticks must gorge on blood, often increasing 100 fold in body size, in order to stimulate tissue development, ecdysis, mating and reproduction [9]. Precisely how the tick's nervous system regulates these biological processes is largely unknown. During feeding, some synganglion cells, especially the neurosecretory cells, increase in size and accumulate neurosecretory substances [11], [12].

Neurohormones and neurotransmitters play key roles in tick development and physiology. Work has been conducted examining their occurrence in selected tissues in ticks such as the hemocytes [13], midgut [14], ovaries [13], and salivary glands [2], [13], [15]–[20]. However, much of the work on neurotransmitters focuses on the dopaminergic system in the salivary glands (reviewed by [21]–[23]). Much less is known about the transcribed genes in the tick synganglia, largely because of the difficulty in extracting sufficient amounts of tissue. Advances in sequencing technology now allow researchers to rapidly obtain large amounts of data from a small amount of tissue. Recent work by Simo et al. [24] showed the existence of a complex neuropeptidergic network extending to different body tissues as well as within the synganglion. However, the molecular basis for understanding just how these complex neurohormone-controlled networks regulate tick physiological functions remains to be determined. To this end, a global search to identify and characterize the numerous molecules expressed in the synganglion is needed.

Transcriptomics offers an excellent tool to approach this problem [25]. Using 454 pyrosequencing, Bissinger et al. [26] generated a cDNA library of expressed genes in the synganglion of adult female American dog ticks, *Dermacentor variabilis* and predicted many of the neuropeptides, neuropeptide receptors, neurotransmitters, iron transport proteins, transmembrane proteins (e.g., tetraspanins), stress reduction proteins and numerous housekeeping genes. Previous studies by Donohue et al. [27] using similar methods identified 14 neuropeptides and 5 neuropeptide receptors in this same species. Transcriptome analysis of the synganglion of the brown dog tick, *Rhipicephalus sanguineus*, was done with cDNA library construction in phage resistant *Escherichia coli*. However, this method yielded a total of only 1008 ESTs sequenced, with only 603 remaining after removal of vector contamination that could be clustered into unique transcripts [20]. Neuropeptides and their receptors have also been predicted in several species of hard ticks using bioinformatics [28] and immunohistochemistry [24] and identified by MALDI-TOF/TOF mass spectrometry [29]. Despite these few studies, there is a paucity of information about the neurobiology of ticks.

Solexa/Illumina (HiSeq), 454 pyrosequencing, and other next generation sequencing technologies provide high levels of coverage (millions of base pairs) far exceeding previous methods requiring cloning into *E. coli* [30]. Along with improvements in assembly technology these advances have greatly enhanced the value of transcriptomes for the study of messages encoding for diverse genes in selected tissues or whole organisms [31], even in organs as small as the tick synganglion.

Transcriptomes provide an opportunity to examine at high resolution the entire repertoire of mRNA molecules expressed in a particular organ or tissue at a particular moment in time. Transcriptomes have proven useful for the study of gene expression of many arthropods, including mosquitoes [32], bedbugs [33] and other arthropods, as well as selected organs, such as the transcriptome of the midgut of female *D. variabilis* [14] and the male reproductive system of this same species [34].

Here we present the first transcriptome of the synganglion of the black legged tick, *I. scapularis,* with identification of messages predicting neuropeptides, neuropeptide receptors, as well as receptors for neurotransmitters, oxidative stress peptides, reproduction-related peptides and many others in this important organ. We conducted BLAST matching against the published conspecific genome and gene sequence alignments which we believe are likely to increase the reliability of gene annotations in the relevant transcriptomes. Using Solexa/Illumina sequencing, we were able to create a large EST library with more than 100 million raw reads (8.49 billion bases) suitable for *de novo* transcriptome assembly and gene annotation/analysis. In view of the short read length (from 68–110 bp) characteristic of this technology at the time sequencing was done, we also used 454 pyrosequencing (∼450 bp read length) which was expected to offer significant advantages resulting from greater read length [25] to help recognize messages predicting genes that might have been missed by Illumina sequencing. In this report, we compare the success of these two different technologies in identifying the many messages predicting genes of interest for our understanding of synganglion function in this important tick species.

We believe that the assembled, annotated transcriptomes described in this report will provide an invaluable resource for future studies of the role of the genes involved in neurologic functioning of the tick nervous system.

## Materials and Methods

### Ticks

Black-legged ticks, *I. scapularis* were reared as previously described (Sonenshine, 1993) and originated from specimens collected near Armonk, New York, USA. Adult ticks were confined within plastic capsules attached to New Zealand white rabbits, *Oryctolagus cuniculus*, and allowed to feed to repletion and oviposit. Larvae and nymphs were allowed to feed on albino mice, *Mus musculus*. Engorged ticks were held in a Parameter Generation and Control incubator (Black Mountain, N.C.) at 26±1°C, 92±1% relative humidity and 14:10 (L:D) for oviposition or molting.

### Ethics statement

All use of animals in this study was carried out in strict accordance with the recommendations in the Guide for the Care and Use of Laboratory Animals of the National Institutes of Health. The protocols were approved by the Old Dominion University Institutional Animal Care and Use Committee ((Animal Welfare Assurance Number: A3172-01). The approved protocols (#10-018 and #10-032) are on file at the Office of Research, Old Dominion University, Norfolk, Virginia. Tranquilizers (Acepromazine) were administered to the animals prior to handling to minimize anxiety and/or discomfort.

### Synganglion RNA collection and sample preparation

Adult *I. scapularis* females, either unfed, partially-fed 5–6 days (virgin) or replete (mated), were dissected, the synganglia were excised and then washed in phosphate-buffered saline on ice (PBS: pH 7.0, 10 mM NaH_2_PO_4_, 14 mM Na_2_HPO_4_, and 150 mM NaCl). The cleaned tissues were homogenized in Qiagen RLT buffer and total RNA extracted in accordance with the manufacturer's recommendations (Qiagen, Valencia, CA). Samples were collected and frozen (−80°C) until needed.

Three different RNA samples were prepared, 1) 5.1 µg of total RNA from a mixture of ∼50 unfed, part-fed virgin and replete females for Illumina sequencing (sample Il-1); 2) 3.28 µg total RNA from ∼45 part-fed virgin females for Illumina sequencing (sample Il-2); and 3) 3.25 µg total RNA from ∼30 part-fed virgin females for 454 pyrosequencing (sample 454). RNA yield and purity (260/280) were determined using a Nanodrop 2000 spectrophotometer (Thermofisher, Wilmington, DE). Samples with low purity (<1.8) were discarded.

### Illumina sequencing

For Illumina sequencing, samples extracted at ODU were submitted to the North Carolina State University Genome Sciences Laboratory and prepared for sequencing using the Illumina TruSeq RNA Sample Prep Kit v2 (Part No. 15026495, Illumina, Inc. San Diego, CA). The integrity of the individual RNA samples was evaluated using the Bioanalyzer 2100 (Agilent Technologies, Santa Clara, CA); samples that did not meet minimum requirements (RNA integrity ≥8) were discarded. A minimum of 1.0 µg high quality total RNA was used for Illumina sequencing.

Following PCR amplification, adapters were included for sequencing with paired ends. For paired ends, Illumina GA-II sequencing adapters were ligated to the fragments, as described by Illumina's Paired-End Sample Preparation Guide (catalogue number PE-930-1001).

### 454 pyrosequencing

For 454 pyrosequencing, the total RNA was thawed and mRNA isolated from each of 3 female synganglion samples, using an Oligotex mRNA isolation kit according to the manufacturer's recommendations. The samples were evaluated using the Bioanalyzer 2100 as described above to insure that they met the minimum requirements for 454 sequencing. Purified mRNA was ethanol precipitated, rehydrated in 2 µl of RNase-free water and combined with 10 pmol of modified 3′ reverse transcription primer (5′-ATTCTAGAGACCGAGGCGGCCGACATGT_(4)_GT_(9)_CT_(10)_VN-3′) [35] and 10 pmol SMART IV oligo (5′-AAGCAGTGGTATCAACGCAGAGTGGCCATTACGGCCGGG-3′) [36]. The resulting 4 µl were incubated at 72°C for 2 min and then combined with the following reagents on ice: 1 µl RNase Out (40 U/µl, 2 µl 5X first strand buffer, 1 µl 20 mM DTT, 1 µl dNTP mix (10 mM each) and 1 µl Superscript II reverse transcriptase) (Invitrogen, Carlsbad, CA). The reaction was incubated at 42°C for 90 min then diluted to 30 µl with TE buffer (10 mM Tris HCL pH 7.5, 1 mM EDTA) and stored at −20°C until further use. To synthesize second strand cDNA, 5 µl of first-strand cDNA was mixed with 10 pmol of modified 3′ PCR primer (5′-ATTCTAGAGGCCGAGGCGGCCGACATGT_(4)_GTCT_(4)_GTTCTGT_(3)_CT_(4)_VN-3′) [35], 10 pmol of 5′ PCR primer (5′-AAGCAGTGGTATCAACGCAGAGT-3′) [36], 5 µl 10X reaction buffer, 1 µl dNTP mix, 2 µl MgSO_4_, 0.4 µl Platinum HiFi Taq Polymerase and 34.6 µl H_2_O (Invitrogen). Thermal cycling conditions were 94°C for 2 min followed by 20 cycles of 94°C for 20 sec, 65°C for 20 sec and 68°C for 6 min. For optimization of the PCR reaction, 5 µl aliquots from cycles 18, 22 and 25 were analyzed on a 1% agarose gel. An additional 5 reactions were carried out with 20 cycles (the optimized number of cycles) to produce sufficient quantities of cDNA for preparation of the 454 library. Following PCR, the contents from the different samples were combined into a single sample and the cDNA was purified using a PCR purification kit (Qiagen) according to the manufacturer's recommendations.

For 454 pyrosequencing, the cDNA library was prepared with the Standard Flex Platform kit (GSLR70 sequencing kit, Cat. No. 04 932 315 001; Roche, Branford CT and Qiagen, Indianapolis, IN) for pyrosequencing on the GS-FLX sequencer according to the manufacturer's recommendations which have been described previously [27], [34], [37]. The only deviation from the protocol was that DNA-positive beads were enriched after emulsification PCR in order to increase the number of reads collected during titration. Enrichment was done so that only beads containing DNA were loaded and the data generated during titration sequencing could also be used in the assembly of contiguous sequences. Enrichment of DNA-positive beads was completed exactly as described by Margulies et al. [37].

### Bioinformatics

Assembly was done using CLC-BIO program. We first trimmed the sequences of ambiguous bases, low quality base calls, and by removing any latent primer or adapter sequences. The *de novo* assembly resulted in ∼41,000 transcripts for sample Il-1 and ∼30,800 transcripts for Il-2 versus 20,600 transcripts for sample 454. Transcripts were identified (annotated) by BLAST [37] against the GenBank nr and EST databases at an E-value of E-06 (or lower) for the Illumina assemblies and E-10 for 454 (although with few exceptions, only matches E-06 or lower were accepted for inclusion in the data tables). Gene Ontology (GO) categorizations of the functional annotations of the top BLASTx hits (1E-06 cutoff) for biological processes (BP) levels 2 and 3 were done using the program Blast2GO [38], [39] in December 2010 for the Illumina transcripts and May, 2011 for the 454 transcripts. Functional assignments were based on an e-6 cut-off (with selected exceptions as justified by other evidence) and conserved domain matches (SMART, KOG and Pfam databases). Additional BLAST searches were done for selected transcripts of interest against the conspecific *I. scapularis* genome, ver. IscaW1.05.1 (www.vectorbase.org).

Additional evidence to support the annotation of transcripts of interest in the different transcripts was done using alignments with conspecific genes or other arthropod genes. Alignments comparing transcriptome transcripts with the conspecific genome and other species were done using Geneious (Biomatters <system@biomatters.com>) and the alignment programs provided.

With few exceptions, transcripts that showed less than 80% pairwise similarity with the conspecific *I. scapularis* genome or other species were regarded as unsupported and were removed from the lists of annotated genes.

### Data deposition

The Illumina transcriptomes were deposited in the **NCBI Sequence Read Archive** under project number PRJNA230499. The mixed unfed-fed-replete female synganglion biosample was deposited under sample number Biosample SAMN0249371, accessible with the following link: http://www.ncbi.nlm.nih.gov/biosample/SAMN02429371. The Transcriptome Shotgun Assembly (TSA) project for the part-fed virgin female synganglion assembly was deposited at DDBJ/EMBL/GenBank under accession number GBBN00000000. The version described in this paper is the first version, GBBN01000000. It is accessible with the following link http://www.ncbi.nlm.nih.gov/biosample/SAMN02678957. The Roche 454 pyrosequencing raw reads file for the part-fed virgin female synganglion assembly was deposited in the NCBI Sequence Read Archive under project number PRJNA242857 with SRR # SRR1214480. The 454 transcriptome was deposited in the TSA and will be published pending curation. The transcriptome fasta file is also available in “Supporting Information”.

## Results and Discussion

### Raw reads, base pairs and assembly

The results of Illumina and 454 sequencing for the three different samples are shown in Table 1. Following sequencing, the raw reads were assembled with the CLC-BIO assembly program (CLC Bio, Cambridge, MA).

**Table 1:** Statistical summary of results of deep sequencing of female synganglion RNA extracts of the tick, *Ixodes scapularis* by Illumina and 454 pyrosequencing.

Sample Il-1. Mixed unfed, partfed & replete females by Illumina			
Parameter	Count	Average length (bp^1^)	Total read length (total bases)
Reads	34,520,330	68	2,346,107,140
Matched	34,273,479	68	2,331,047,269
Not matched	246,851	61	15,059,871
No. Contigs	41,249	480	19,809,620

Sample Il-2. Part-fed females by Illumina			
Parameter	Count	Average length (bp^1^)	Total read length (total bases)
Reads	117,900,476	72	8,488,834,272
Matched	43,351,571	NR^2^	3,121,313,112
Not matched	74,548,905	NR^2^	5,367,521,160
Contigs	30,838	655	20,206,192

Sample 454. Part-fed females by 454 pyrosequencing			
Parameter	Count	Average length (bp^1^)	Total read length (total bases)
Reads	456,073	267	109,342,701
Matched	394,946	267	105,795,232
Not matched	61,127	58	3,547,469
Contigs	20,630	523	10,979,742

1 bp  =  base pairs.

2 NR  =  Not reported.

For sample Il-1 (mixed females), sequencing yielded a total of 34,520,330 raw reads for a total read length of 2,346,107,140. Almost all reads were matched (34,273,479). The average read length (matched reads) was 68 bp, and included a total length of 2,331,047,269 bp. Following vector trimming, the raw reads were assembled to a total of 41,249 transcripts with an average length of 480 bp, comprising 19,809,620 bp.

For sample Il-2 (part-fed females), sequencing yielded a total of 117,900,476 raw reads. The average length was 72 bp and included a total of 8,488,934,272 bp. However, less than half were matched reads (43,351,571), comprising only 3,121,313,112 bp. Following vector trimming, the raw reads were assembled to a total of 30,838 transcripts with an average length of 655 bp, comprising 20,206,192 bp.

For sample 454 (part-fed females), sequencing yielded a total of 394,946 raw reads. The average length was 268 bp and included a total length of 105,795,232 bp. Following vector trimming, the raw reads were assembled to a total 20,630 transcripts, with an average length of 523 bp, comprising 10,979,742 bp.

**Figure 1:**
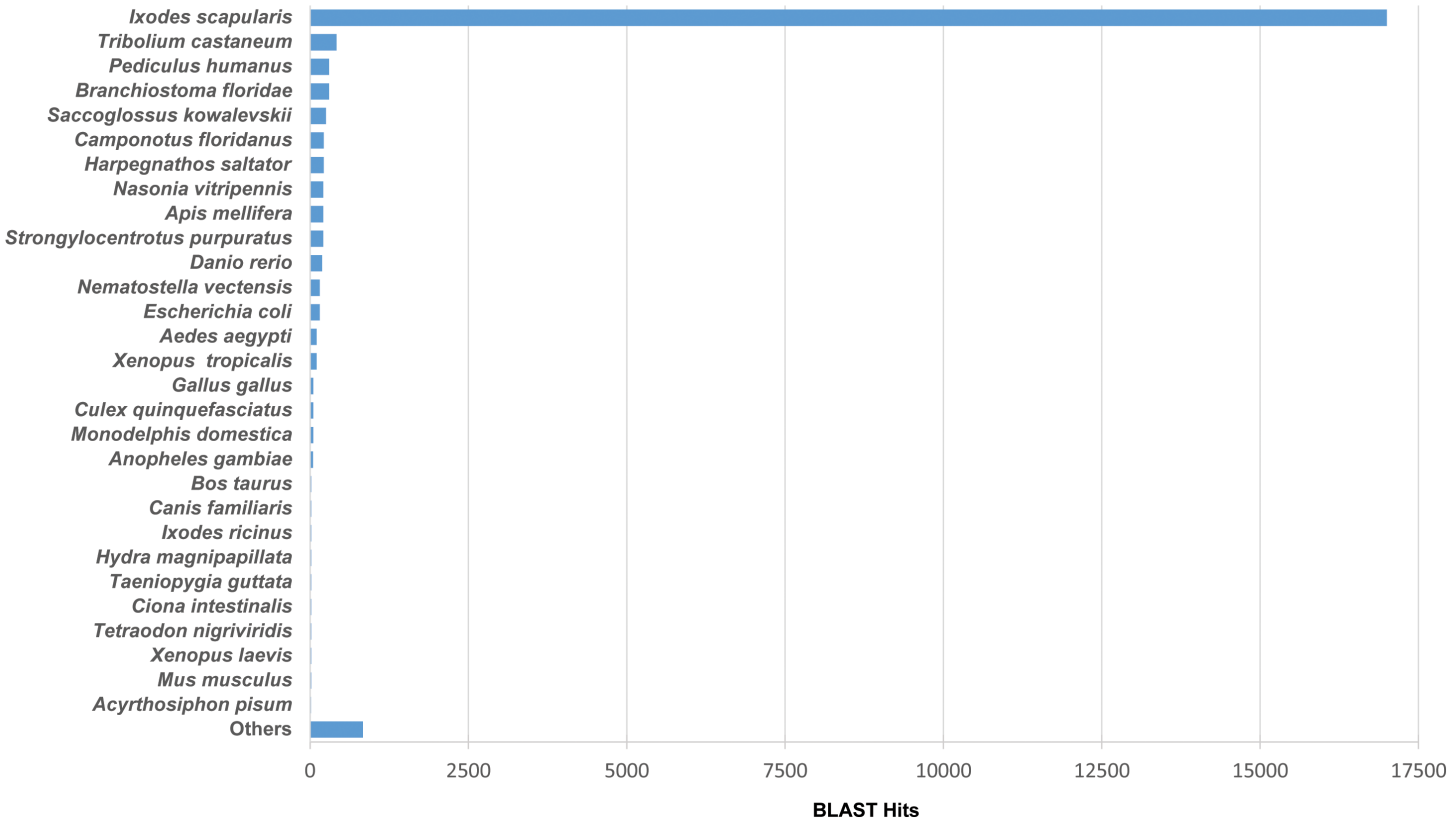
Blast matching of contig files in the transcriptomes showing the top hit species distribution.

BLAST matching of the transcript files showed that almost all of the top hits matched sequences from the *I. scapularis* genome (Fig. 1). Approximately 17,000 sequences matched similar sequences from *I. scapularis*, or approximately 91.5% of all matched transcripts. Similar findings were made with BLAST matches from samples Il-1 and 454 (data not shown).

### Gene ontology

**Figure 2:**
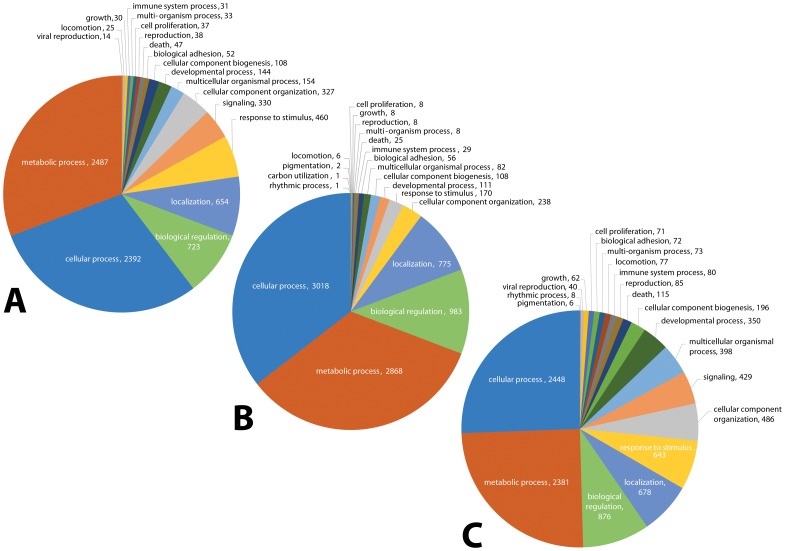
Gene Ontology (GO) term assignments at biological processes level 2 for the three transcriptomes assembled following sequencing by Illumina or 454. Fig. 2A shows the 19 GO term assignments for sample Il-1 (8,086 transcripts); Fig. 2B shows the 20 GO term assignments for sample IL-2 (8,445 transcripts); Fig. 2C shows the 21 GO term assignments for sample 454 (9,574 transcripts).

The program BLAST2GO was used to map the top BLASTx matches (e-value 1e-06) and to assign gene ontology (GO) term annotations [38], [39] in December 2011. For Biological Processes (BioPro) at level 2, GO term searching was highest for sample 454 (46.4%) as compared to only 19.6% and 27.4% for the two Illumina samples. GO term searching success of less than 50% of the expressed genes has been reported in transcriptome studies of other ticks, e.g., the *D. variabilis* synganglion transcriptome [26] and the *D. variabilis* male reproductive organs. The GO term assignments for all BioPro level 2 are shown in Fig. 2. Overall, there was little difference in the GO assignments in these transcriptomes, although there were 38 transcripts assigned to reproduction in Il-1(mixed females) as compared to only 8 transcripts in sample Il-2 (part-fed females) versus 85 transcripts in sample 454. Categories considered of greatest interest for regulating synganglion biological activity, development and reproduction in the 3 transcriptomes are shown in Table 2 (BioPro Level 2).

**Table 2:** Gene Ontology categories in three different transcriptomes from samples of the *Ixodes scapularis* female synganglion: GO terms searching success, similarities and differences.

BioProcess Level 2 (GO term)		% Sample Il-1	% Sample Il-2	% Sample 454
	Metabolic and cellular processes	60.3	69.7	50.4
	Biological regulation	8.9	11.6	9.2
	Signaling	4.0	4.1	4.5
	Response to stimulus	5.7	2.0	6.7
	Developmental processes	1.9	1.3	3.7*
	Immunity	0.4	0.3	0.8
	Locomotion	0.5	0.2	0.8
	Growth	0.4	0.1	0.7
	Reproduction	0.5	0.1	0.9
	Totals (% all categories)	82.5	89.5	73.2
	Search success: total transcripts/(% all transcripts)	8,086 (19.6%)	8,445 (27.5%)	9,574 (46.4%)
**BioProcess Level 2 (GO term)**	Metabolic and cellular processes	50.8	46.3	46.5
	Biological regulation	5.3	5.9	7.3
	Oxidation-reduction	4.1	3.3	3.1
	Signaling	0.8	5.2*	1.3
	Cellular response to stimulus	3.0	0.8*	3.3
	Response to external stimuli	1.4	1.0	3.0
	Cellular developmental processes	0.5	0.7	1.3*
	Immunity	0.2	0.3	0.3
	Hormonal metabolic processes	0.6	2.4*	0.1
	Growth	0.0	0.1	0.2
	Reproduction	0.5	0.3	0.9
	Totals (% all categories)	67.2	66.3	67.3
	Search success: total transcripts/(% all transcripts)	11,740 (28.5%)	23,573 (76.4%)	14,249 (69.1%)
**Molecular Level 3 (GO term)**	Hydrolase activity	13.1	12.7	11.8
	Transferase activity	12.6	11.9	9.5
	Ion binding	11.7	10.8	8.3
	Nucleotide binding	8.8	9.2	10.7
	Nucleoside binding	0.1	6.8*	6.5
	Nucleic acid binding	9.4	10.3	7.8
	Tetrapyrrole binding	1.2	0.8	1.0
	Protein binding	8.4*	10.0*	9.1
	Carbohydrate binding	0.5	0.5	0.6
	Carboxylic acid binding	0.3	0.2	0.2
	Lipid binding	0.5	0.4	2.0
	Oxireductases activity	6.5	4.8	8.0
	Signal transducer activity	3.4	4.4	2.9
	Neurotransmitter binding	---	0.1	0.1
	Kinase regulatory activity	---	0.1	0.2
	Transmembrane transporter activity	3.0	2.9	2.8
	Nucleoside-triphosphatase activity	1.0	1.0	0.2
	Enzyme inhibitor activity	0.7	0.5	0.8
	Enzyme activator activity	0.6	0.5	0.2
	Totals (selected gene categories)	81.8	65.5	83.3
	Search success: total transcipts/(% all transcripts)	8,160 (19.8%)	17,660 (57.3%)	2,629 (12.74%)

* Major difference.

For BioPro level 3, there was little difference between samples Il-2 and 454 (76.4% and 69.1%), but both samples had much higher GO term searching success than sample Il-1. GO term searching success for these samples was comparable to studies of transcriptomes of other tick tissues sequenced by 454 pyrosequencing, e.g., the transcriptome of the *D. variabilis* male reproductive organs (30.2%, [34] and synganglion (29.3%, [26] and the salivary transcriptome of the black-legged tick *I. scapularis* (66%) [16]. It is likely that the large number of transcripts that could be not be mapped was due to novel and/or unknown sequences which have not been annotated, a phenomenon reported previously for transcriptomes of other tick tissues [16].

**Figure 3:**
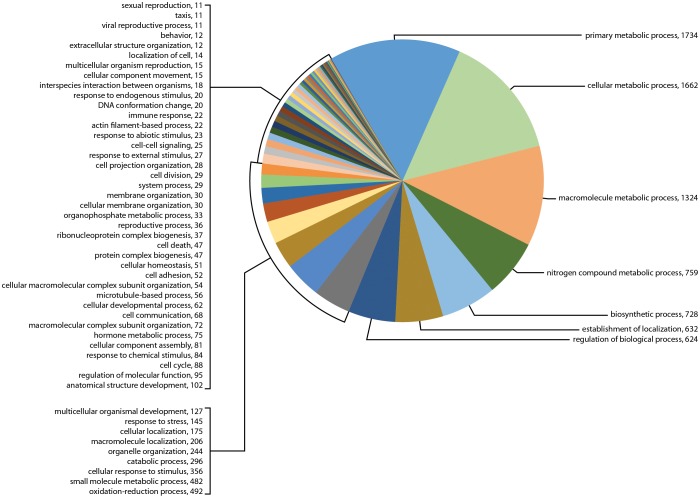
Gene Ontology (GO) term assignments at biological processes level 3 for the transcriptome for sample Il-1 assembled following sequencing by Illumina. This figure shows the 56 GO term assignments categorized in this transcriptome (11,740 transcripts).

**Figure 4:**
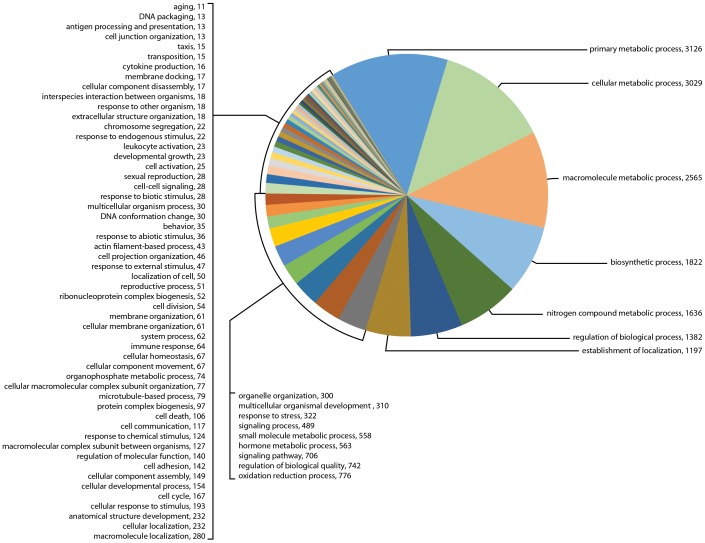
Gene Ontology (GO) term assignments at biological processes level 3 for the transcriptome for sample Il-2 assembled following sequencing by Illumina. This figure shows the 84 GO term assignments categorized in this transcriptome (23,573 transcripts).

**Figure 5:**
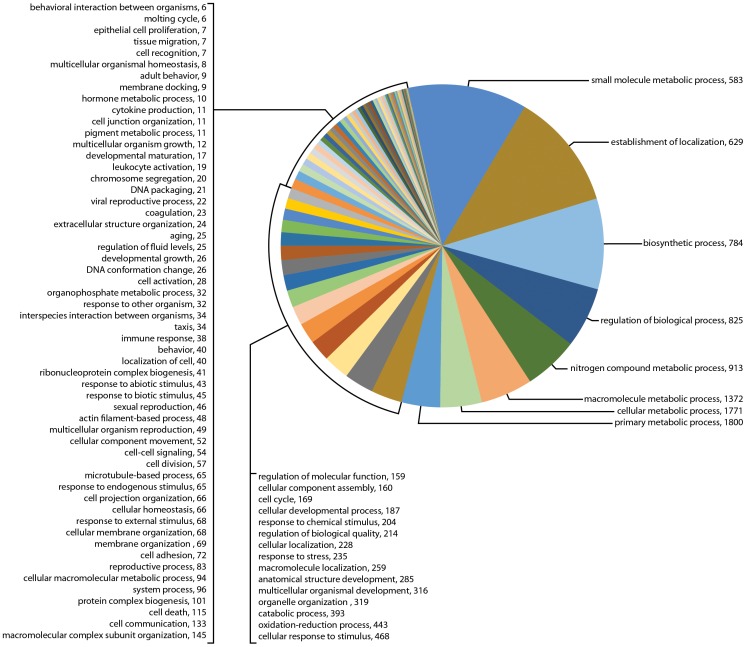
Gene Ontology (GO) term assignments at biological processes level 3 for the transcriptome for sample 454 assembled following sequencing by 454. This figure shows 81 GO term assignments categorized in this transcriptome (14,249 transcripts).

The GO term assignments for biological processes at level 3 are shown in Figures 3, 4, 5 and selected categories of interest in Table 2 (BioProcesses Level 3). Many more categories regulating synganglion biological activity were found than were identified in BioPro level 2, including biological regulation, oxidation-reduction, cellular response to stimulus, cell to cell and other signaling types, cellular developmental processes, hormone metabolic processes, immune response and reproduction. Of special interest for sensory perception was the number of messages for responses to external, abiotic and chemical stimuli, representing as much as 1.4% of genes in sample Il-1, 1.0% in sample Il-2 and 3.0% in sample 454. Transcripts matching genes involved in cell to cell signaling, signaling processes, cell to cell communication and signaling pathways represented only 0.8% of total genes in sample Il-1 versus much larger percentages in the other transcriptomes, specifically as much as 5.2% of the total genes in sample Il-2 and 1.3% in sample 454 (Table 2).

Comparison of the *I. scapularis* synganglion 454 transcriptome with the *D. variabilis* transcriptomes (also created by 454) [26] showed that, for Bioprocesses Level 2, the synganglion of *I. scapularis* expressed a much higher number of transcripts involved in response to stimulus, biological regulation and developmental processes than similar processes in the synganglion of *D. variabilis*. Whether this reflects true differences in expression or is a function of the larger proportion of genes mapped in *I. scapularis* (46.4%) versus the proportion searched in *D. variabilis* (29.3%) is unknown.

**Figure 6:**
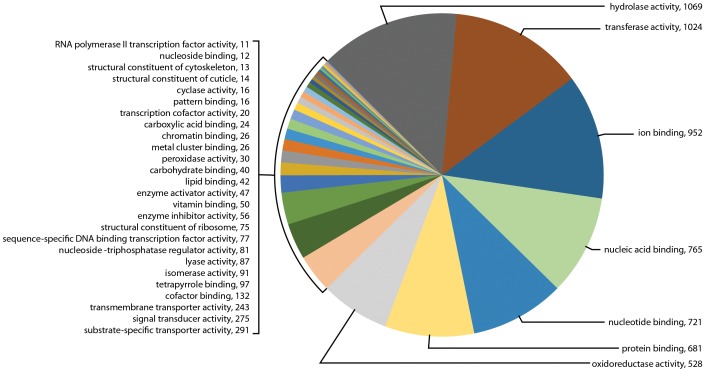
Gene Ontology (GO) term assignments at molecular level 3 for the transcriptome for sample Il-1 assembled following sequencing by Illumina. This figure shows the 34 GO assignments categorized in this transcriptome (8,160 transcripts).

**Figure 7:**
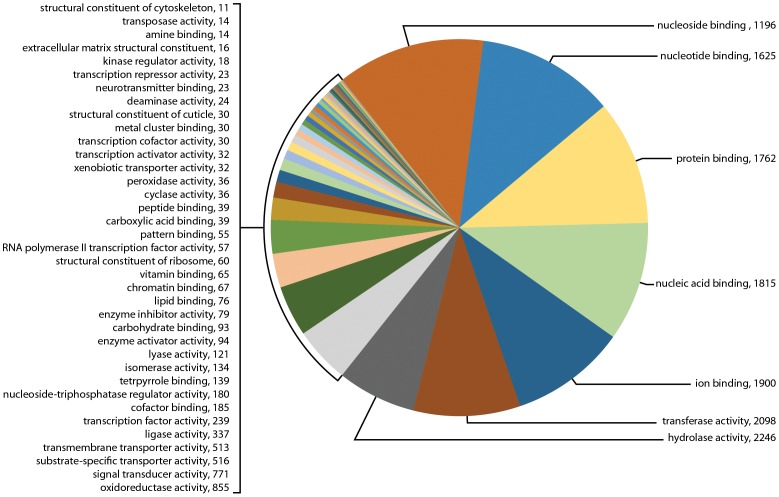
Gene Ontology (GO) term assignments at molecular level 3 for the transcriptome for sample Il-2 assembled following sequencing by Illumina. This figure shows the 44 GO assignments categorized in this transcriptome (17,660 transcripts).

**Figure 8:**
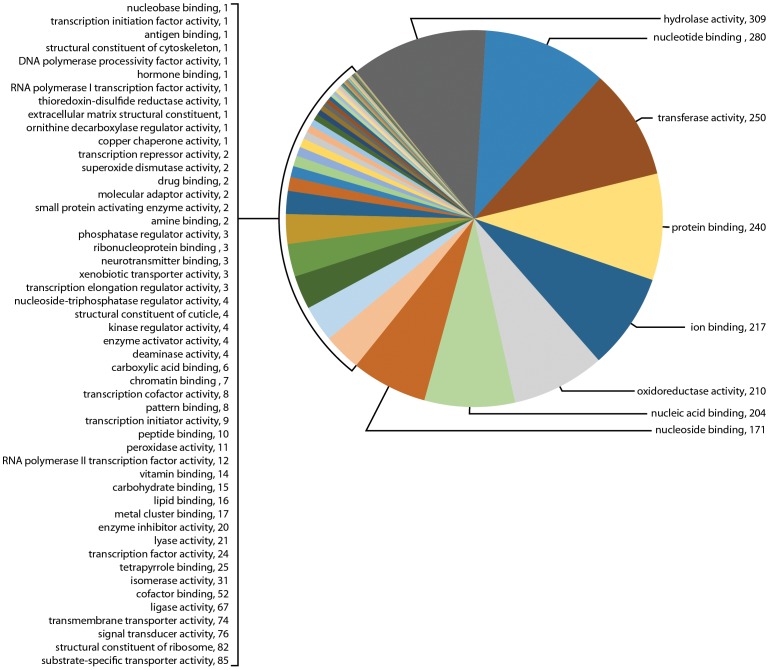
Gene Ontology (GO) term assignments at molecular level 3 for the transcriptome for sample 454 assembled following sequencing by 454. This figure shows the 58 GO assignments categorized for this transcriptome (2,629 transcripts).

In addition to biological functions, GO term searching also examined the numerous GO categories by molecular function. At molecular level 3, GO term searching of the transcriptome for sample Il-1 showed 34 categories with a total of 8,160 genes, or 19.8% of all transcripts (Fig. 6). The most abundant categories were hydrolase activity (1,069), transferase activity (1,024), ion binding (952), nucleic acid binding (765), nucleotide binding (721), protein binding (681), oxireductase activity (528), and signal transducer activity (275). GO term searching of the transcriptome for sample Il-2 showed 44 categories with a total of 17,660 genes, or 57.3% of all transcripts (Fig. 7). The most abundant categories were hydrolase activity (2,246), transferase activity (2,098), ion binding (1,900), nucleic acid binding (1,815), protein binding (1,762), nucleotide binding (1,625), oxireductase activity (885), and signal transducer activity (771). Of special interest for synganglion regulatory activity was neurotransmitter binding (23 genes) and kinase regulatory activity (18 genes) (highlighted in yellow). In contrast, although the transcriptome for sample 454 showed 58 categories, the total number of genes mapped was only 2,629 genes, or 12.7% of all transcripts (Fig. 8). The most abundant categories were hydrolase activity (309), nucleotide binding (280), transferase activity (250), protein binding (240), ion binding (217), oxireductase activity (210), nucleic acid binding (204), nucleoside binding (171) and signal transducer activity (76). Little evidence of neurotransmitter receptors or neuropeptides was found at this level, namely neurotransmitter receptor binding with 3 genes and peptide binding with 10 genes. However, others may have been present but incorporated into broader categories.

Table 2 shows the contrasts for 19 GO molecular categories of interest for the three different transcriptomes as percentages of the total searched genes. Little difference was noted in the percentages of gene messages in these GO categories among the three different transcriptomes.

### Detailed analysis of selected genes/gene categories of interest

Tables 3, 4, 5, 6, 7, 8, 9, 10, 11, 12 compare the three different transcriptomes with respect to the numbers of transcribed genes (i.e., messages), transcript frequency and species matched for 12 gene categories of special interest for understanding synganglion function (<e-06). Transcript frequency indicates the number of transcripts that predicted the same gene identified in GenBank and should not be misconstrued as a measure of gene expression. Variations in transcript frequency may be due to errors in assembly and/or annotations of the same gene in different species. These categories include neuropeptides, neuropeptide receptors, neurotransmitter receptors, other GPCRs, hormone receptors, iron transport/iron storage compounds, immune peptides, reproduction-related compounds, steroid receptor, oxidative or environmental stress compounds and chitin synthesis/cuticle associated compounds.

**Table 3:** Synopsis of 12 major gene categories in the *Ixodes scapularis* female synganglion with comparison of the different transcriptomes-neuropeptides.

	Sample Il-1		Sample Il-2		Sample 454	
Neuropeptide Name (GO:0005184)^a^	No. Trans^b^	Species^c^	No. Trans^b^	Species^c^	No. Trans^b^	Species^c^
Allatostatin	1	*I. scapularis*	0	**-----**	1	*D. variabilis*
Allatotropin	0	**-----**	1	*I. scapularis*	1	*I. scapularis*
Bursicon-α	1	*I. scapularis*	0	*----*	0	*----*
CRF^d^	2	*I. scapularis*	0	*----*	0	*----*
CRF^d^-binding protein	0	**----**	2	*I. scapularis/C. floridanus*	0	**----**
Eclosion hormone	0	**----**	1	*I. scapularis*	0	**----**
FMRFamide	0	**----**	1	*“*	0	**----**
Glycoprotein A	1	*I. scapularis*	1	*“*	0	**----**
Insulin-like peptide	3	*“*	1	*“*	0	**----**
Ion transport peptide	5	*“*	2	*“*	0	**----**
Myoinhibitory peptide	1	*“*	0	*-----*	0	**----**
Neurophysin-isotocin	1	*C. comersonii*	1	*C. commersonii*	0	**----**
Orcokinin 5	1	*I. scapularis*	0	**-----**	0	**-----**
Sulfakinin	1	*D. variabilis*	1	*I. scapularis*	0	**-----**
Precursor SIFamide	1	*I. scapularis*	0	*I. scapularis*	0	*-----*
**No. Neuropeptides/(No Transcripts)**	**11 (18)**		**9 (11)**		**2 (2)**	

a GO terms are Bioprocesses definitions from the Gene Ontology Consortium (www.geneontology.org).

b No. Trans  =  number of Transcripts. Transcript frequency indicates the number of transcripts that were annotated with the same gene identified in GenBank and should not be misconstrued as a measure of gene expression. If multiple transcripts matched the same GenBank accession number, only the transcript with the highest e-value matching the specific accession number was included. High contig frequency may have been due to errors in assembly and/or annotations of the same gene in different species. Transcripts were annotated as a particular gene message based on strong (i.e., low) e-value and very high % sequence alignment. See Table 13 for representative examples and see text for more detailed descriptions of these methods.

c Full names of abbreviated species at the end of Table 12.

d Abbreviations of scientific terms: CRF  =  corticotropin releasing factor.

Total all neuropeptides  =  15; Note: in some cases, more than one neuropeptide may result via post-translational modifications from a single prepropeptide mRNA. Transcripts for Pre-protein convertase, an enzyme essential for conversion of neuropeptides to the mature form, also were found.

**Table 4:** Synopsis of 12 major gene categories in the *Ixodes scapularis* female synganglion with comparison of the different Transcriptomes – neuropeptide receptors.

	Sample Il-1		Sample Il-2		Sample 454	
Receptor Name (GO:0008188)^a^	No. Trans^b^	Species^c^	No. Trans^b^	Species^c^	No. Trans^b^	Species^c^
Allatostatin	3	*I. scapularis*	5	*I. scapularis*	0	**-----**
Calcitonin	5	*I. scapularis*	0	**-----**	2	*I. scapularis*
CCAP^d^	3	*I. scapularis/T. castaneum*	1	*T. castaneum*	0	**-----**
Corazonin	1	*I. scapularis*	1	*I. scapularis*	0	**-----**
CRF^d^	0	*-----*	4	*"*	0	**-----**
Eclosion hormone	1	*I. scapularis*	1	*I. scapularis*	0	**-----**
GnRN/AKH-like^d^	1	*I. scapularis*	1	*"*	0	**-----**
Insulin receptor	4	*I. scapularis*	3	*"*	0	**-----**
Neuropeptide F	0	**-----**	2	*"*	0	**-----**
Proctolin	0	**-----**	0	**-----**	1	*D. pulex*
Pyrokinin	0	**-----**	3	*I. scapularis/D. variabilis*	0	**-----**
SIFamide	1	*I. scapularis*	1	*I. scapularis*	0	**-----**
Sulfakinin	0	**-----**	1	*C. quinquefasciatus*	0	**-----**
Tachykinin	0	*-----*	1	*N. vitripennis*	1	*N. vitripennis*
**No. Receptors/(No. transcripts)**	**8 (19)**		**12 (24)**		**3 (4)**	

Footnotes a and b as in Table 3.

c Full names of abbreviated species at the end of Table 12.

d Abbreviations of scientific terms: CCAP  =  Cardioacceleratory peptide; CRF  =  Corticotropin releasing factor;

GnRH/AKH-like  =  Gonadotropin releasing hormone/adipokinetic hormone-like.

**Table 5:** Synopsis of 12 major gene categories in the *Ixodes scapularis* female synganglion with comparison of the different Transcriptomes –Neurotransmitter receptors and transporters.

	Sample Il-1			Sample Il-2	Sample 454	
Receptor Name (GO:0045213)^a^	No. Trans^b^	Species^c^	No. Trans^b^	Species^c^	No. Trans^b^	Species^c^
Acetylcholine	10	*I. scapularis*/others^1^	13	*I. scapularis*/others^1^	3	*I. scapularis*
GABA^d^	2	*I. scapularis*	3	*I. scapularis/R. microplus*	0	*-----*
GABA transporter	8	*"*	7	*I. scapularis*	5	*I. scapularis*
Dopamine	5		6	*"*	1	*Gallus gallus*
Glutamate (NMDA^d^, Ionotropic, Metabotropic)	21	*I. scapularis*/others^1^	24	*"*	5	*I. scapularis*/others^1^
Na+- neurotransmitter/Symporter	3	*I. scapularis*	3	*"*	0	*-----*
Octopamine	4	*"*	6	*"*	2	*A. gambiae*/others^1^
Serotonin	2	*I. scapularis*	3	*"*	0	-------
**No. Receptors/(No. Transcripts)**	**8 (55)**		**8 (65)**		**5 (16)**	

Footnotes a and b as in Table 3; **^c^** Full names of abbreviated species at the end of Table 12; **^d^**Abbreviations of scientific terms:

GABA  =  γ-aminobutyric acid; NMDA  =  N-methyl-D-aspartate.

1 Others include: *A. gambiae; A. meliffera*; *A. pisum; A. suum; B. mori; C. floridanus; C. intestinalis; D. rerio; D. melanogaster; G. gallus; H. americanus; Homo sapiens; N. vitripennis; P. humanus; P. pseudoannulata; R. microplus; S. kowalevski; T. verrucosum; T. castaneum.*

**Table 6:** Synopsis of 12 major gene categories in the *Ixodes scapularis* female synganglion with comparison of the different Transcriptomes –Other GPCR receptors.

	Sample Il-1		Sample Il-2		Sample 454	
Receptor Name (GO:0007218^a^)	No. Trans^b^	Species^c^	No. Trans^b^	Species^c^	No. Trans^b^	Species^c^
Pheromone odorant receptor	1	*I. scapularis*	2	*I. scapularis*	1	*I. scapularis*
Gustatory receptor	1	*N. vitripennis*	0	-----	1	*N. vitripennis/others*
GPCRs unidentified	28	*I. scapularis/P. humanus*	49	*I. scapularis/An. gambiae*	1	*S. purpuratus/others^1^*
**No. GPCRs/(No. Transcripts)**	**3 (30)**		**2 (51)**		**3 (3)**	

Footnotes a and b as in Table 3; **^c^** Full names of abbreviated species at the end of Table 12; ^1^Others include: *N. vitripennis; T. castaneum; D. pulex.*

**Table 7:** Synopsis of 12 major gene categories in the *Ixodes scapularis* female synganglion with comparison of the different Transcriptomes –Hormone/other steroid proteins and receptors.

	Sample Il-1		Sample Il-2		Sample 454	
Receptor Name (GO:0035076/GO:0003707/GO:0050810^a^)	No. Trans^b^	Species^C^	No. Trans^b^	Species^C^	No. Trans^b^	Species^c^
Ecdysone nuclear receptor	0	-----	4	*I scapularis/others* 1	0	-----
JH esterase binding Protein	0	-----	2	*I. scapularis/A. mellifera*	0	-----
Other steroid receptors	3	*I. scapularis*	3	*I. scapularis*	4	*I. scapularis*
Steroidogenic acute regulatory protein	1	*“*	1	*“*	1	*I. scapularis/others* 1
**No. receptors/(No. Transcripts)**	**2 (4)**		**4 (10)**		**2 (5)**	

Footnotes a and b as in Table 3; **^c^**Full names of abbreviated species at the end of Table 12.

1 Others include: *T. castaneum/P. humanus corporis*.

**Table 8:** Synopsis of 12 major gene categories in the *Ixodes scapularis* female synganglion with comparison of the different Transcriptomes –Reproduction/developmental proteins and enzymes.

	Sample Il-1		Sample Il-2		Sample 454	
Protein/enzyme name/(GO:0048609)^a^	No. Trans^b^	Species^c^	No. Trans^b^	Species^c^	No. Trans^b^	Species^c^
Spermatogenesis-associated protein	5	*I. scapularis/others* 1	3	*I. scapularis*	2	*I. scapularis/C. familiaris*
Major sperm protein	1	*I. scapularis*	1	*"*	0	*-----*
n -acetyl-spermine/spermidine oxidase	2	*"*	2	*"*	1	*I. scapularis*
Spermidine synthase	0	*-----*	1	*"*	1	*A. variegatum*
Epididymal secretory protein	2	*I. scapularis*	1	*“*	2	*I. scapularis*
**No. receptors/(No. Transcripts)**	**4 (10)**		**5 (8)**		**4 (6)**	

Footnotes a and b as in Table 3; **^c^**Full names of abbreviated species at the end of Table 12.

1 Others include: *Gallus gallus; Callithrix jacchus*.

**Table 9:** Synopsis of 12 major gene categories in the *Ixodes scapularis* female synganglion with comparison of the different Transcriptomes –immune peptides/proteins.

	Sample Il-1		Sample Il-2		Sample 454	
Peptide/protein name/(GO006955)^a^	No. Trans^b^	Species^c^	No. Trans^b^	Species^c^	No. Trans^b^	Species^c^
Defensin	2	*I. scapularis/I. ricinus*	2	*I scapularis/I. ricinus*	3	*H. longicornis/others* 1
Hemolectin	6	*I. scapularis/others* 1	8	*I. scapularis/others* 1	1	*I. scapularis*
Ixoderin	7	*I. scapularis/I. ricinus*	4	*I. scapularis*	1	*H. longicornis*
Galectin	3	*I. scapularis*	1	*"*	2	*I. scapularis*
Peptidoglycan recognition proteins	1	*"*	1	*"*	1	*R. microplus*
Microplusin	1	*"*	4	*"*	0	*-----*
Αlpha-macroglobulin	3	*"*	0	-----	0	-----
Subolesin	1	*“*	4	*I. scapularis*	0	*------*
**Total peptides/(No. Transcripts)**	**8(24)**		**7 (24)**		**5 (8)**	

Footnotes a and b as in Table 3; **^c^**Full names of abbreviated species at the end of Table 12.

1 Others include: *A. mellifera*; *D. variabilis; D. marginatus; S. kowalevskii; T. castaneum*.

**Table 10:** Synopsis of 12 major gene categories in the *Ixodes scapularis* female synganglion with comparison of the different Transcriptomes –Oxidative stress.

	Sample Il-1		Sample Il-2		Sample 454	
Peptide/enzyme name (GO: 0055114)^a^	No. Trans^b^	Species^c^	No. Trans^b^	Species^c^	No. Trans^b^	Species^c^
Glutathione S-Transferase	32	*I. scapularis/M.musculus*	24	*I. scapularis/I. pacificus*	18	*I. scapularis/others* 1
Oxidative stress induced-growth	3	*I. scapularis*	1	*I. scapularis*	0	*------*
Oxireductase	8	*I. scapularis/others* 1	7	*I. scapularis/others* 1	1	*I. scapularis/others* 1
Thioredoxin	6	*I. scapularis/others* 1	13	*I. scapularis/others* 1	12	*I. scapularis/others* 1
Superoxide dismutase	4	*I. scapularis/B. floridae*	4	*I. scapularis*	6	*I. scapularis/others* 1
**Total peptides/(No. Transcripts)**	**5 (53)**		**5 (49)**		**4 (37)**	

Footnotes a and b as in Table 3; **^c^**Full names of abbreviated species at the end of Table 12.

1 Others include: *A. variegatum; D. variabilis; C. floridanus; C. intestinalis; H. sapiens; H. marginatum rufipes; I. pacificus; I. ricinus; M. musculus; N. vitripennis; R. norvegicus; R. sanguineus; S. purpuratus; T. infestans*.

**Table 11:** Synopsis of 12 major gene categories in the *Ixodes scapularis* female synganglion with comparison of the different Transcriptomes –Environmental stress.

	Sample Il-1		Sample Il-2		Sample 454	
Peptide (GO: 006950)^a^	No. Trans^b^	Species^c^	No. Trans^b^	Species^c^	No. Trans^b^	Species^c^
Heat shock 20	3	*I. scapularis/I. pacificus*	1	*I. scapularis*	5	*I. scapularis/D. variabilis*
Heat shock 40	0	*-----*	1	*“*	0	-----
Heat shock 70	23	*I. scapularis/others* 1	13	*I. scapularis/A. albimanus*	6	*I. scapularis*
Heat shock 90	1	*I. scapularis*	6	*I. scapularis/others* 1	7	*I. scapularis/others* 1
**Total peptides/(No. Transcripts)**	**3 (27)**		**4 (21)**		**3 (18)**	

Footnotes a and b as in Table 3; **^c^**Full names of abbreviated species at the end of Table 12.

1 Others include: *A. aegypti*; *A. gambiae; B. floridae; C. japonica; Psilochlorus sp.; H. longicornus; H. sapiens; O. cuniculus.*

**Table 12:** Synopsis of 12 major gene categories in the *Ixodes scapularis* female synganglion with comparison of the different Transcriptomes – Cuticle associated.

	Sample Il-1		Sample Il-2		Sample 454	
Enzyme (GO: 006950)^a^	No. Trans^b^	Species^c^	No. Trans^b^	Species^c^	No. Trans^b^	Species^c^
Chitin synthase	3	*I. scapularis/M. brassicae*	5	*I scapularis/others* 1	0	-----
Chitinase	1	*I. scapularis*	1	*I. scapularis*	5	*I scapularis*/*others* 1
**Total Peptides/(No. transcripts)**	**2 (4)**		**2 (6)**		**1 (5)**	

Footnotes a and b as in Table 3; **^c^**Full names of abbreviated species in tables 3 – 12 are cited below.

1 Others include: *A. gambiae; C. quinquefasciatus; M. brassicae; N. vitripennis; P. humanus; R. sanguineus; T. vaginalis*

Species abbreviations all scientific names used in tables 3 - 12: *A. californica  =  Aplysia californica; A. aegypti  =  Aedes aegypti; A. gambiae  =  Anopheles gambiae; A. mellifera  =  Apis mellifera; A. variegatum  =  Amblyomma variegatum; A. pisum  =  Acyrthosiphon; A. suum  =  Ascaris suum; B. mori =  Bombyx mori; B. floridae  =  Branchiostoma floridae; C. floridanus*  =  *Camponotus floridanus; C. camersonii  =  Catastomus comersonii; C. quinquefasciatus  =  Culex quinquefasciatus; D. variabilis  =  Dermacentor variabilis; D. pulex  =  Daphnia pulex; G. gallus  =  Gallus gallus; H. longicornis  =  Haemaphysalis longicornis; H. diversicolor  =  Haliotis diversicolor; H. saltator  =  Harpegnathos saltator; H. sapiens  =  Homo sapiens; I. scapularis =  Ixodes scapularis; M. brassicae  =  Mamestra brassicae; M. domestica  =  Monodelphis domestica; P. tricornutum  =  Phaseodactylum tricornutum; N. vitripennis  =  Nasonia vitripennis; O. cuniculus  =  Oryctolagus cuniculus; P. americana  =  Periplaneta americana; P. tunicata =  Pseudoalteromonas tunicata; P. humanus  =  Pediculus humanus (corporis); Psilochlorus sp.; R. microplus*  =  *Rhipicephalus microplus; R. norvegicus  =  Rattus norvegicus; S. kowalevskii  = Saccoglossus kowalevskii; S. frugiperda  =  Spodoptera frugiperda; S. purpuratus*  =  *Strongylocentrotus purpuratus; T. castaneum  =  Triboleum castaneum; T. vaginalis  =  Trichomonas vaginalis; T. verrucosum  =  Trichophyton verrucosum; X. tropicalis  =  Xenopus tropicalis.*

### Neuropeptides and neuropeptide receptors

The number of true neuropeptides in ticks is uncertain. Estimates of expressed neuropeptides range from as few as 20, characterized by proteomic methods ([29] to as many as 80, characterized by *in silico* searches of publicly accessible EST databases [28]. In the most recent estimate, genes for 56 neuropeptides have been identified in *I. scapularis,* of which 15 are believed novel. Fifty one neuropeptides and/or neurohormones were reported to occur in another acarine, the spider mite (*Tetranynchus urticae*) [40].

Of the 56 neuropeptide genes believed to occur in this tick (Hill et al. pers. commun.), transcripts for mRNA encoding for 15 neuropeptides and 14 neuropeptide receptors were recognized in the transcriptomes of the *I. scapularis* synganglion. Comparing our findings with the transcriptome of *D. variabilis* done by 454 pyrosequencing [26], [27], we also found transcripts encoding for several of the same neuropeptides, specifically, encoding for allatostatin, and encoding for the peptides bursicon, glycoprotein A, eclosion hormone, insulin-like peptide, ion transport peptide, orcokinin, and sulfakinin. The actual number of mature peptides is likely somewhat greater since in some cases several neuropeptides can be processed via post-translational modifications from a prepropeptide, e.g., the transcript mRNA annotated as allatostatin also aligns with *I. scapularis* allatostatin B and *D. variabilis* allatostatin (Fig. S9). We also found transcripts encoding for 7 other neuropeptides, namely, allatotropin, corticotropin-releasing factor (CRF), FMRFamide, myoinhibitory peptide, neurophysin-isotocin, neuropeptide F, and SIFamide (precursor) that were not found in the transcriptome of the *D. variabilis* synganglion. We found transcripts encoding for four of the same neuropeptide receptors, i.e., calcitonin receptor, gonadotropin-releasing hormone/AKH-like receptor, pyrokinin receptor, and sulfakinin receptor that also were found in the synganglion of *D. variabilis*; we did not find evidence of a leucokinin-like receptor. In addition, we found transcripts encoding for 10 other neuropeptide receptors, i.e., allatostatin, cardioacceleratory peptide, corazonin, CRF, eclosion, insulin-like peptide, sulfakinin, proctolin, SIFamide and tachykinin. Supporting evidence for these gene assignments in the *I. scapularis* synganglion is shown in the sequence alignments in the supplementary figures (Figures S1, S2, S3, S4, S5, S6, S7, S8, S9, S10, S11, S12, S13, S14, S15, S16, S17, S18, S19, S20, S21, S22, S23). Comparing the transcripts from the transcriptomes versus the conspecific genes, we found 95.8% pairwise identity for allatostatin (Fig. S9), 100% pairwise identity for allatotropin (Fig. S1), 95.7% pairwise identity for glycoprotein A (Fig. S3), 99.2% pairwise identity for insulin-like peptide (Fig. S4), 69.7% pairwise identity for myoinhibitory peptide (Fig. S15); 98.2% pairwise identity for insulin-like peptide receptor (Fig. S17); 51.2% pairwise identity for sulfakinin receptor (Fig. S18); 99.2% pairwise identity for the tachykinin receptor (Fig. S20); and 89.2% pairwise identity for orcokinin 5 (Fig. S7). For the transcript encoding for sulfakinin, the closest match, 78.3%, was with a similar neuropeptide in *D. variabilis* (Fig. S14). Although not a neuropeptide, another noteworthy finding was the occurrence of transcripts encoding for pro-protein convertase in the two Illumina transcriptomes (3 in each, respectively) similar to that found in *D. variabilis*; sequence alignment showed 100% pairwise identity with the conspecific gene (Fig. S8). Proprotein convertase is essential for the conversion of neuropeptide hormones to the mature form and their subsequent secretions [41] by endoproteolytic cleavage [27]. However, we did not find a transcript encoding for periviscerokinin, previously identified only by MALDI-TOF mass spectrometry [29]. Although we did not find transcripts encoding for the peptides calcitonin, corazonin, gonadotropin-releasing hormone/AKH-like and pyrokinin, neuropeptides reported to occur in the *D. variabilis* synganglion [27], we did find transcripts encoding for their receptors, strongly suggesting that the messages for these peptides are also expressed (Fig. S13, calcitonin receptor, 95.8% pairwise sequence alignment with conspecific gene; Fig. S12 corazonin receptor, 100% pairwise identity with the conspecific gene; Fig. S19, gonadotropin-releasing hormone/AKH-like receptor, 68.1% pairwise sequence alignment with the conspecific gene; and Fig. S10, pyrokinin receptor, 62.2%, pairwise sequence alignment with the conspecific gene; respectively).

Interestingly, we found the transcripts encoding for bursicon α in *I*. *scapularis* (supported by Fig. S5, 59.5% pairwise identity with the conspecific gene) and glycoprotein A, but no evidence of transcripts encoding for bursicon β or glycoprotein B; both bursicon α & β and glycoprotein A & B mRNA were found in the synganglion of *D. variabilis*. Similarly, we only found a transcript encoding for one orcokinin (orcokinin 5), whereas transcripts encoding for 4 different orcokinins were found in the *D. variabilis* synganglion transcriptome. We also found transcripts encoding for corticotropin-releasing factor (CRF) receptor, CRF-binding protein, eclosion hormone and FMRFamide in the *I. scapularis* synganglion transcriptome. For supporting evidence, see Fig. S2, CRF-binding peptide, 100% pairwise sequence alignment with conspecific gene; Fig. S6, eclosion hormone, 97.7% pairwise identity with the conspecific gene; Fig. S11, FMRFamide, 62% pairwise identity with the conspecific gene; and Fig. S16, SIFamide receptor, 100% identity with the mature peptide of the conspecific gene, respectively. These are messages for neuropeptides that were not detected in the *D. variabilis* synganglion. We also found a transcript encoding for neuropeptide F, the importance of which is discussed below; for supporting evidence, see Fig. S21, showing 100% pairwise sequence alignment with the conspecific gene. Also of interest was the finding of the transcript encoding for the cardioacceleratory peptide (CCAP) receptor (see Fig. S22). Also known as crustacean cardioactive peptide (CCAP), this neuropeptide is a member of the inotocin (vasopressin/oxytocin) family that regulates arthropod cardiac activity, digestion, and even reproductive activity. The finding of the transcript encoding for the CCAP receptor, suggests that the message for the corresponding CCAP peptide may also be present even though not detected in the transcriptomes. Finally, also noteworthy was the finding of transcripts predicting ion transport peptide (Fig. S23), a molecule important for chloride transport and water reabsorption in insects and, presumably, in many other organisms.

### Presumed physiological functions of synganglion neuropeptides and receptors

Our findings suggest that in ticks, including *I. scapularis,* the hormonal regulation of water balance and diuresis during blood feeding is done by several neuropeptides, specifically calcitonin, CRF-DH and possibly periviscerokinin. Another possible regulator of tick water balance is inotocin. Although found in many insects [42], we did not find transcripts encoding for their occurrence in the *I. scapularis synganglion* transcriptome, nor were they found by Bissinger et al. [26] in the transcriptome of the *D. variabilis* synganglion. However, we did find messages encoding for neurophysin/isotocin (inotocin), the carrier proteins for these neuropeptides, suggesting their expression in the tick synganglion. Moreover, genes for oxytocin/vasopressin (inotocin) have been annotated in the *I. scapularis* genome.

The hormonal regulation of development during blood feeding and reproduction in ticks is not well understood. Messages encoding several of the neuropeptide hormones (or their receptors) known to regulate these processes in insects [43] were found in the transcriptomes of the *I. scapularis* synganglion including allatotropin, allatostatin, bursicon α, corazonin, glycoprotein A, gonadotropin releasing hormone/AKH-like receptor, insulin-like peptide, orcokinin, sulfakinin and pyrokinin. Most of these same neuropeptides were reported to be differentially expressed in the *D. variabilis* synganglion [26]. The presence of messages encoding for allatostatin and allatotropin in the *I. scapularis* synganglion is surprising since ticks do not produce juvenile hormone [44]. However, there is evidence that some elements of the JH pathway occur in ticks (Roe, R.M., Zhu, J. and Bissinger, B., unpublished) even though the final branch leading to JH is absent. Bissinger et al [26] showed that allatostatin was significantly upregulated after mating and feeding to repletion, suggesting a role in tick reproduction. These authors note that “Allatostatins in insects have other functions in addition to the regulation of JH synthesis that might be applicable to ticks”. Clearly, the evidence for its role in tick feeding and/or reproduction is compelling, but the precise nature of that role remains to be discovered.

Also of interest were the messages encoding for eclosion hormone, bursicon and corazonin in adult female *I. scapularis*. These hormones are associated with ecdysis and cuticle sclerotization in insects. However, ixodid ticks, including *I. scapularis*, do not molt as adults, although they do undergo extensive remodeling of the integumental system during blood feeding [9] so as to allow for the enormous expansion of their body size and subsequent oviposition. Another significant finding was that of a transcript matching the receptor for pyrokinin in *I. scapularis* as well as pyrokinin receptor in *D. variabilis* [27]. It functions in diverse roles in insects [45] but there is no reported evidence of a specific functional role in ticks. The gene for pyrokinin was also reported to occur in the *I. scapularis* genome [28]. BLAST (nr) comparison of the transcript #570 (transcriptome Il-2, Table S1) showed significant alignments with genes for the pyrokinin receptor from *I. scapularis* and *D. variabilis* (Figure S10); also from various insects (e.g., *Tribolium castaneum*, 1e-18, XM 963710 and *Apis mellifera,* 2e-17, NM_001164008). Several neuropeptides are known to have broad physiological functions, not specific to any one organ or organ process. In vertebrates, neurophysin-oxytocin ( =  vasopressin-oxytocin) is believed to stimulate smooth muscle contraction; however, its functions in invertebrates ( = inotocin) are unclear [42]. Neuropeptide F (NPF), the invertebrate version of mammalian neuropeptide Y (NPY) [46] also appears to have several functional roles. Modulation of ion transport by NPF was found in the foregut of the mosquito *Aedes aegypti* and by NPY in the human intestine [47].

We did not find any evidence of the leucokinin receptor reported to occur in the *D. variabilis* synganglion [27] or messages encoding for the receptors for bombyxin, neuroparsin, prothoracicotropic hormone (PTTH), pre-ecdysis triggering hormone (PETH) or pigment dispersing factor (PDF), previously reported to occur in insects [48] in the any of the *I. scapularis* transcriptomes. Immunoreactive staining showed PDF and PTTH reactive neurons in the synganglion of *Rhipicephalus appendiculatus* [24]. However, ETH expression is not believed to occur in the synganglion. Immunoreactive staining suggested ETH activity in pairs of cells termed “pedal endocrine cells” but not in the synganglion of *R. appendiculatus* and *Ixodes ricinus* [24], [49]. However, immunoreactive staining alone may not be compelling evidence of ETH gene expression.

### Comparison between the three different female *I. scapularis* synganglion transcriptomes

Combining the BLAST matches from the three transcriptomes enabled us to predict a total of 15 expressed neuropeptides. In addition, transcripts encoding for receptors for calcitonin, cardioacceleratory peptide, corazonin, gonadotropin-releasing hormone/AKH-like, neuropeptide F, proctolin, pyrokinin and tachykinin were found, suggesting that the true total of neuropeptide messages was at least 23 (Tables 3 and 4). Comparison of the transcripts encoding for neuropeptides receptors found in the three different transcriptomes shows that many more were found by Illumina sequencing, specifically 11 neuropeptides (18 transcripts) in sample Il-1 and 9 neuropeptides in sample Il-2 (11 transcripts) than were found by 454 pyrosequencing, specifically 2 neuropeptides (2 transcripts). This was also the case for neuropeptide receptors, with 8 predicted (19 transcripts in sample Il-1), 13 in sample Il-2 (25 transcripts) versus only 3 (4 transcripts) in sample 454, respectively. A message encoding for only one neuropeptide, proctolin, was found solely by 454 pyrosequencing. Messages encoding for only 2 neuropeptides, allatostatin and allatotropin, and only 2 neuropeptide receptors, calcitonin, and tachykinin were found by both Illumina and 454 pyrosequencing methods. Clearly, although there were benefits obtained by each method of high throughput sequencing, messages encoding for many more neuropeptides and their receptors, with a higher frequency of specific transcripts, were obtained using the Illumina platform than by 454 pyrosequencing.

### Neurotransmitter receptors, transporters and neuromodulators

Transcripts encoding receptors for 6 different types of neurotransmitters were recognized, namely, acetylcholine (Ach), gamma aminobutyric acid (GABA), dopamine, glutamate, octopamine, and serotonin. Transcripts encoding for acetylcholine muscarinic and nicotinic receptors and 3 types of glutamate receptors had the highest frequency in the *I. scapularis* synganglion. Transcripts encoding two different types of GABA receptors (ion-channel and metabotropic) and three different types of glutamate receptors (ionotropic, metabotropic and NMDA) were recognized (Table 5). In addition, transcripts encoding the enzyme acetylcholinesterase, which degrades acetylcholine, also were recognized. Sequence alignments supporting the functional assignments of these transcripts are shown in Fig. S24, S25, S26, S27, S28, S29, S30, S31.

ACh and the enzyme AChE occur in most animals [50] including ticks [14], [20], [26], [51]. Transcripts encoding both muscarinic (See Fig. S24) and nicotinic receptors were found. Many more transcripts encoding for these molecules were found in the transcriptome of the *I. scapularis* synganglion than in *D. variabilis*. Thirty separate transcripts were found for AChE, matching 30 separate published GenBank sequences in the NCBI (nr) database (Table S1).

The inhibitory neurotransmitter, γ-aminobutyric acid (GABA) and its receptors are found in insects (and most other animals) [52], [53]. GABA and GABA receptors are known to occur in ticks, although little is known about their precise functions (summarized by Simo et al.[54]). Bissinger et al. [26] identified 2 GABA receptors and 2 GABA transporters in the synganglion of *D. variabilis.* The transcriptomes of the *I. scapularis* synganglion revealed 5 transcripts encoding for GABA receptors (both metabotropic GABA, Fig. S26, and ionotropic GABA), plus 20 transcripts matching the published sequences for GABA transporter. Most were found by Illumina sequencing; no transcripts encoding for GABA receptors were found by 454 pyrosequencing (Table 5).

Transcripts encoding for glutamate receptors were the most abundant (49.4%) of all the neurotransmitter receptors found in the *I. scapularis* synganglion transcriptomes. Glutamate is a major excitatory neurotransmitter in the nervous system and at the neuromuscular junction of insects and crustaceans. It can act via inotropic and metabotropic receptors. In insects, muscle contraction is controlled by the glutamatergic pathway [55]. Two glutamate-gated chloride channel receptors were reported in the synganglion of *R. appendiculatus* [20] and one glutamate receptor interacting protein (GRIP) was found in the transcriptome of the *D. variabilis* synganglion. In contrast, a remarkable number of transcripts matching sequences for glutamate receptors, including all three types (NDMA, ionotropic and metabotropic), were found in the transcriptomes of the *I. scapularis* synganglion (21 in sample Il-1, 24 in sample Il-2 and 5 in sample 454) (See Fig. S27, Fig. S28 and Fig. S29 for sequence alignments). In addition, numerous transcripts for glutamate synthase were identified (6 in sample Il-1, 3 in sample Il-2 and 1in sample 454, not shown in Table 5). It is not clear why so many more glutamate receptor and glutamate synthase transcripts were uncovered by Illumina than 454 (Table 5).

Transcripts encoding for the neurotransmitter receptors for the monamines dopamine, octopamine and serotonin were also very numerous in the *I. scapularis* synganglion. In insects, dopamine functions in modulation of memory recall and motor behavior [56]. In ticks, one of the most important functions of dopamine is stimulation of salivary secretion via the dopaminergic pathway [24], [54]. Dopamine was also reported to stimulate cuticle plasticization during blood feeding, which is essential for allowing growth in body size [57]. Transcripts encoding for two dopamine receptors were reported in the transcriptome of the *D. variabilis* synganglion [26]. Here we report 11 transcripts encoding genes for dopamine receptors found in the *I. scapularis* synganglion by Illumina versus only 1 for a dopamine receptor found by 454 (Table 5) (See Fig. S25 for sequence alignment).

Octopamine is the invertebrate ortholog of noradrenaline/norepinephrine in vertebrates In insects, octopamine modulates neuromuscular activity thereby enhancing glutamate stimulation of muscle [55]. An octopamine receptor was found in the *D. variabilis* synganglion transcriptome [26] and evidence of similar receptors have been described in other species (reviewed by Simo et al.). Here we report the frequent occurrence of transcripts encoding for octopamine receptors in the synganglion of *I. scapularis*, with 12 transcripts found in the 3 transcriptomes (4 in sample Il-1, 6 in sample Il-2 and 2 in sample 454) (Table 5) (See Fig. S30).

The neurotransmitter serotonin (5-hydroxytryptamine) is believed to occur in ticks, based on evidence of serotonin immunoreactivity in the tick nervous system [19]. Only one transcript encoding for a receptor for serotonin was found (sample Il-1) (Fig. S31); no transcripts encoding for serotonin receptors were found in either of the other two samples. We also report numerous transcripts predicting receptors for a Na+-transmitter symporter, molecules that transport cations into cells innervated by dopaminergic and/or serotoninergic neurons. In cockroaches, it is believed that these transporters are involved in the secretion of the NaCl-rich primary saliva [58], a finding import for ticks in which regulation of the salt composition of saliva is essential for control of body water balance (Table 5).

### Other GPCR/hormone receptors

In addition to the neuropeptide receptors described above, other GPCRs were identified in the three transcriptomes. This includes neuropeptide F, found in samples Il-1 and Il-2, as noted previously (Table 4). Neuropeptide F has multiple physiological roles, including feeding, metabolism, reproduction and stress responses [46]. This is the first report of its occurrence in the synganglion of ticks. Its role in these parasites remains to be established.

Transcripts encoding GPCRs functioning as pheromone odorant and gustatory receptors were also identified in the *I. scapularis* synganglion. This is surprising since sensory receptors are part of the peripheral nervous system and normally found in sensory organs near or at the exterior of the body and appendages. However, evidence of atypical expression of such genes, at least the gustatory receptors, has been reported in *Drosophila* [53]. Since the sex pheromone of *I. scapularis* has not been identified, the discovery of these odorant receptors may prove useful for recognizing the ligand. The same may be true for the gustatory receptors (Table 6).

In addition to these 3 GPCRs, transcripts encoding for numerous other GPCRs for which no specific function has been assigned were also found, namely 28 in sample Il-1, 49 in sample Il-2 and 1 in sample 454. A total of 65 neuropeptide GPCR genes were identified in the genome of the spider mite (*T. urticae*), many of which were orthologs of GPCRs in insects, while others had no identifiable orthologs in other genomes, i.e., had no match [40].

### Other hormone and steroid receptors

A transcript predicting an ecdysone nuclear receptor was identified in the transcriptome of the part-fed female synganglion (Il-2) but not in either of the other two transcriptomes. Alignment of this transcript with the *I. scapularis* sequence in Genbank showed a 100% match (Fig. S32). In *D. variabilis*, the ecdysteroid 20-hydroxyecdsyone increases gradually during blood feeding. However, following mating and rapid engorgement to repletion, its concentrations increase greatly [59]. The much higher expression of this hormone stimulates vitellogenesis and oogenesis [60]. Thus, the expression of this receptor only in sample Il-2 (part-fed female synganglion) does not appear to be consistent with these reported phenotypic effects. However, expression of an ecdysone-activated ribosomal protein L63 was found in both Illumina transcriptomes (not shown in Table 7), suggesting that ecdysone activity was prevalent in both the virgin and mated female synganglia.

A transcript encoding for an ecdysone receptor was also found in the synganglion of *D. variabilis* (which included samples from mated/replete as well as fed virgin females). Similar to the latter species, this may “suggest that ecdysteroids have some role in the regulation of synganglion function during female reproduction in ticks” [26]. Transcripts encoding for other steroid receptors of unknown identity were found in all three transcriptomes while one transcript encoding for juvenile hormone esterase binding protein were found in sample Il-1 and 2 transcripts encoding for this protein were found in sample Il-2 (Table 7). Transcripts for steroid reductase dehydrogenase were also found in all 3 transcriptomes.

### Reproduction and development-related transcripts

Transcripts predicting 5 different genes with functions associated specifically with sperm or spermatogenesis were expressed in the female synganglion transcriptomes. Of the 5 different genes in this category, transcripts encoding for epididymal secretory protein E1 (similar to Nieman-Pick C2 protein), spermidine synthase (see Fig. S33), n-acetyl spermine (spermidine oxidase) and major sperm protein were also found in the *D. variabilis* female synganglion transcriptome. Transcripts predicting expression of these male-specific genes in the female synganglion of this tick is unexpected. We were unable to find evidence of similar expression of sperm or spermatogenesis-related genes in the female brains/CNS of insects, nematodes, mollusks or other invertebrates. However, examination of the sequence for the spermatogenesis-associated protein revealed that it contained a domain also found in the SSP411 protein family, commonly found in spermatids and suggesting a function in fertility regulation. Spermidine was reported to enhance neuronal differentiation in cultures of insect tissues (crickets, *Acheta domesticus*). However, it is not known whether this effect also occurs *in vivo* [61]. A contig encoding for spermine (peroxisomal N(1)-acetyl-spermine/spermidine oxidase), found in the *I. scapularis* synganglion, was also found in the transcriptome of the *D. variabilis* male reproductive organs [34] as well as in the *I. scapularis* genome (gene ISCW005123; GB EEC05394). In mosquitoes, e.g., *Anopheles gambiae,* male accessory gland and sperm associated proteins are transferred to the female during copulation and it is likely that some of these proteins initiate signaling to the insect brain [62], [63] and stimulate subsequent brain regulatory activity. Epididymal secretory protein E1 is reported to regulate capacitation in mammals [64]. Whether similar phenomena occur in ticks is unknown (Table 8).

### Transcripts predicting other genes of interest

These include iron transport/storage peptides, immune peptides, oxidative stress and environmental stress.

Ferritins serve as the primary iron transport/storage peptides in ticks and many other blood-feeding arthropods, essential for removing ferric iron and reducing toxicity. Its expression in blood feeding ticks has also been found to be important as an antimicrobial factor [65], [66]. Multiple transcripts encoding ferritin found in the *I. scapularis* transcriptomes (data not shown) were also found in the transcriptome of the *D. variabilis* synganglion.

Transcripts predicting 8 different types of immune proteins/peptides were found including peptidoglycan recognition proteins (PGRPs), defensin, microplusin, alpha-2-macroglobulin, subolesin and three different lectins. PGRPs are members of a broad class of pathogen-associated molecular pattern (PAMPs) recognition proteins and often initiate signaling activity that leads to inhibition or lysis of the target microbes. Defensins are most often expressed in the hemocytes. However, the *I. scapularis* defensin (scapularisin) was also reported to be expressed in the midgut and fat body [67]. Thus, the occurrence of the transcript predicting defensin (E value 2.98E-147, 98% identity) found in the synganglion of this species extends its occurrence to yet another organ. Transcripts encoding for microplusin and α-macroglobulin also were found. Microplusin is a copper chelating molecule that acts as a bacteriostatic rather than a lytic antimicrobial peptide and is known to act against Gram-positive bacteria [68]. Alpha-2-macroglobulin is a large thioester-type antimicrobial protein that functions by trapping and removing proteases secreted by invading microbes [69]. Transcripts encoding for the other antimicrobial peptides found in the synganglion were lectins, including hemolectin, ixoderin and galectin, important because of their roles in innate immunity. For hemolectin, the largest number of transcripts was found in samples Il-1 and Il-2 (Table 9). No evidence of transcripts encoding for these immunopeptides was reported in the transcriptome of the *D. variabilis* synganglion.

Transcripts predicting genes for combating oxidative and environmental stress were also found. Transcripts encoding glutathione-S-transferase were the most numerous in all three transcriptomes (32 in sample IL-1, 24 in sample Il-2and 18 in sample 454, respectively), followed by transcripts encoding for thioredoxin (6, 13, and 12 in sample 454, respectively), oxireductase (8 in sample Il-1, 7 in sample Il-2 and 1 in sample 454 respectively) and superoxide dismutase (4 in sample Il-1, 4 in sample Il-2, and 6 in sample 454, respectively). Transcripts encoding for oxidative stress-induced growth were found in the two Illumina transcripts, but not in sample 454 (Table 10). The synganglion is one of the most active organs in ticks and must combat oxidative stress, so it is no surprise that transcripts predicting several important genes for these functions were recognized in the transcriptome. In some insect species, oxireductases are also expressed in response to pathogen infection, indicating an important role in immune defense [70].

Transcripts encoding for environmental stress peptides included heat shock (HS) proteins 20, 40, 70 and 90. The great majority of heat shock transcripts were for HS70 (Table 11). Heat shock proteins function in diverse roles, but especially in protecting the cells and tissues by binding to damaged proteins resulting from oxidative and/or environmental stress, thereby preventing them from aggregating; they may also function in post-translational modification, protein transport (chaperones), prevention of apoptosis and other cellular housekeeping roles.

Lastly, transcripts predicting genes for cuticle synthesis and cuticle digestion were also identified in the transcriptomes of the three different synganglion samples. Of special significance for blood feeding adult ticks was the frequent occurrence of transcripts predicting genes for chitin synthesis and chitinase. Chitin is an essential component of the tick's cuticle which undergoes extensive remodeling and growth during blood feeding. In *I. ricinus*, cuticle expansion during the lengthy blood feeding period is accompanied by increases in dityrosine, a molecule believed to function “in stabilizing the cuticular structure during the extensive distension occurring during a blood meal” [71]. Thus it is possible that the presence of transcripts predicting these chitin-associated genes reflects the synganglion's role in regulating cuticle growth during the blood feeding process (Table 12).

As a convenient means for recognizing the neuropeptides, neuropeptide receptors and neurotransmitter receptors identified in this study that regulate specific tick physiologic functions, a table showing the transcripts predicting these genes, organized by their purported function, is included (Table 13).

**Table 13:** Functional role (hypothetical) of neuropeptides and neurotransmitters in adult female *I. scapularis.*

Functional category	Genes predicted in transcriptomes	E-value	Alignment
**Signaling-Neurotransmitters**	Insulin-like peptide receptor –multiple signaling functions	1.3 E-166	99.2%
	Insulin-like peptide –regulates nutrient-dependent growth/metabolism	4.9 E-55	98.2%
	Neurophysin-Oxytocin (inotocin) –multiple signaling functions	8.8 E-22	ND^1^
	Acetylcholine (muscarinic) receptor –major excitatory neurotransmitter	0.0	99.5%
	Γ-aminobutyric acid (GABA) receptor—major inhibitor synaptic transmission	1.0 E-102	93.1%
	Glutamate (Metabotropic) receptor —major excitatory synaptic transmitter	0.0	94.2%
	Octopamine/tyramine –multiple signaling activities	2.4 E-129	93.4%
	Serotonin – stimulates salivary gland secretion, feeding, other functions	7.0 E-27	99.2%
**Salivary gland functions**	Dopamine -stimulates salivary gland secretion	3.5 E-87	93.4%
	Myoinhibitory peptide ( = Allatostatin B) –presumed inhibitor secretion	2.2 E-54	69.7%
	Octopamine/tyramine – regulates salivary gland functions	2.4E-129	93.4%
	SIFamide – regulates secretory activity of salivary glands	5.7 E-35	100%
**Diuresis/H_2_O excess blood meal H_2_O**	Calcitonin (diuretic hormone) –fluid secretion from MT	1.2E-87	100%
	CRF (Corticotropin releasing factor) ( = diuretic hormone) wastes from MT	1.5 E-109	100%
	Ion transport peptide –controls water balance, gut fluid transport	1.8 E-151	100%
	Tachykinin receptor - stimulates gut contractions	9.0 E -54	>99%
**Blood feeding uptake**	FMRFamide –regulate gut muscle contractions	8 E-105	62%
	Neuropeptide F receptor –stimulates feeding	3.3 E-56	100%
	Sulfakinin receptor –downregulation allows increased blood volume	5.5 E-45	51.2%
	Orcokinin –stimulate gut contractions, muscle contractions	5.4 E-41	89.2%
	Tachykinin receptor ---stimulates gut contractions	9.0 E-54	>99%
**Feeding satiety**	sulfakinin –inhibit further feeding (reaches repletion)	2.4 E-27	78.3%
**Ecdysis (molting)**	Bursicon α - insect molting hormone	4.2 E-64	71.2%
	Eclosion hormone - presumed regulates ecdysis behavior	1.5 E-52	97.7%
	Corazonin receptor – stimulate release ecdysis triggering hormone	3.2 E-85	100%
	Cardioacceleratory peptide (CCAP) –regulates heart rate/ecdysis	1.5 E-18	>90%
**Cuticle synthesis**	Bursicon α – —insect molting hormone	4.2 E-64	59.5%
	Eclosion hormone —molting hormone	1.5 E-52	97.7%
**Post-mating reproductive activity**	Allatotropin–stimulates JH pathway, regulates reproduction	4.0 E-46	100%
	Allatostatin ----inhibits elements JH pathway, regulates reproduction	0.0	95.8%
	Myoinhibitory peptide ( = Allatostatin B) inhibits elements JH pathway	0.0	69.7%
	Gonadotropin-releasing hormone/AKH-like – stimulates oocyte growth	2.2 E-54	68.1%
	Pyrokinin (PBAN)—pheromone biosynthesis active hormone, food finding	1.8 E-51	62.2%
	SIFamide –regulates reproductive behavior	5.7 E-35	100%
	Glycoprotein A – regulates reproductive behavior	2.0 E-139	95.7%

ND^1^  =  not done.

## Summary and Conclusions

Transcriptomes have proven useful for global analysis and categorizing of genes from selected tissues or even whole animals. Next generation sequencing has made it possible to examine the complex array of genes associated with selected biological functions in a single sample. Its utility is especially beneficial for comparison with an existing conspecific genome. In this study, we show that Illumina GAIIx sequencing was more efficient than 454 pyrosequencing in terms of total read coverage, GO term searching and annotation of important gene transcripts and gene categories. Total read coverage by Illumina sequencing ranged from 34 million to 117 million reads for the two samples Il-1 and Il-2 whereas coverage by 454 was only 4.6 million reads. For GO biological processes, there was little difference in GO term searching success among the 3 different transcriptomes. However, for GO molecular function, much higher percentages of GO categories were recognized in the samples sequenced by Illumina than by 454. More than 60% of transcripts showing homologies to 19 GO categories were predicted by Illumina sequencing, including many of special interest for synganglion function; only 12.7% were predicted by 454 (Table 2). Moreover, most of the gene matches in samples Il-1 and Il-2 were with *I. scapularis,* 80.7% and 90.2%, respectively, as compared to only 40.5% in sample 454.

Comparison of an in-depth analysis of the transcripts predicting genes in 12 different GO categories also showed higher GO term recognition success with Illumina than with 454 sequencing. For example, of the transcripts encoding for 15 neuropeptides and 14 neuropeptide receptors found in this study, almost all were recognized by Illumina; transcripts for only two transcripts encoding for neuropeptides and three transcripts encoding for neuropeptide receptors were found by 454. Similar differences were found with transcripts encoding for neurotransmitter receptors and transporters. Many more transcripts encoding for other GPCRs were recognized by Illumina (28 in sample Il-1, 49 in sample Il-2) than by 454 (only 1).

We found no evidence to support the hypothesis that 454 pyrosequencing, with the longer read length captured by this technology (267 bp) versus Illumina sequencing (68–72 bp) led to higher success in identifying transcripts, even for large transmembrane receptors or similar large molecules. The diversity of transcripts identified in this study offers a valuable resource that may be mined for further studies that can lead to a better understanding of how the synganglion regulates important physiological functions.

## Supporting Information

Figure S1 **Multiple sequence alignment for allatotropin from the fed female synganglion of the** ***Ixodes scapularis*** **transcriptome versus published sequences (GenBank) from the same and other species.** Multiple sequence alignment (ClustalW) of the deduced amino acid sequence of a putative *I. scapularis* allatropin (contig 8149) from the Illumina sample Il-2 compared with the conspecific *I. scapularis* (Iscap: XP_002407036) and the tobacco horn worm *Manduca sexta* (Msexta: AAB08759). Pairwise identity for contig versus *I. scapularis* sequence from Genbank 100%; multiple identity 43%. Asterisks denote identical residues, dots indicate conservative substitutions.
(TIF)

Figure S2 **Multiple sequence alignments for corticotropin-releasing factor (CRF) binding peptide from the fed female synganglion of the** ***Ixodes scapularis*** **transcriptome versus published sequences (GenBank) from the same and other species**. Multiple sequence alignment (ClustalW) of the deduced amino acid sequence of a putative *I. scapularis* CRF-binding peptide (contig 21180) from the Illumina sample Il-1 compared with the conspecific *I. scapularis* (Iscap: XP_002410820) and the aphid *Acyrthosiphon pisum* (Acyr: XP_003240961). Pairwise identity for contig versus *I. scapularis* sequence from Genbank 100%; multiple identity 59.8%. Asterisks denote identical residues, dots indicate conservative substitutions.
(TIF)

Figure S3 **Multiple sequence alignment for Glycoprotein alpha from the fed female synganglion of the** ***Ixodes scapularis*** **transcriptome versus published sequences (GenBank) from the same the same and other species**. Multiple sequence alignment (ClustalW) of the deduced amino acid sequence of a putative *I. scapularis* Glycoprotein alpha (contig 26726)from the Illumina sample Il-2 compared with the conspecific *I. scapularis* (Iscap: CAR94694) and the American dog tick (Dvar: ACC96601). Pairwise identity for contig 26726 versus the *I. scapularis* sequence from Genbank is 95.7%; multiple identity 83.2%. Asterisks denote identical residues, dots indicate conservative substitutions.
(TIF)

Figure S4 **Multiple sequence alignment for Insulin-like peptide from the fed female synganglion of the** ***Ixodes scapularis*** **transcriptome versus the same and other species**. Multiple sequence alignment (ClustalW) of the deduced amino acid sequence of a putative *I. scapularis* insulin-like peptide (contig 28977)from the Illumina sample Il-1 compared with the conspecific *I. scapularis* (Iscap: XM_002402930) and the American dog tick (Dvar: EU616823). Pairwise identity for contig 28977 versus the *I. scapularis* sequence from Genbank is 99.2%; multiple alignments with the *I. scapularis* and *D. variabilis* sequences are 93.9%. Asterisks denote identical residues, dots indicate conservative substitutions. Contains the I1GF-insulin-bombyxin-like superfamily conserved domain. Note the 4 cysteine residues characteristic of this peptide. Cys1 is linked by a disulfide bond to Cys3, Cys2 and Cys4 are linked by interchain disulfide bonds to cysteines in the "B" chain.
(TIF)

Figure S5 **Multiple sequence alignment for bursicon alpha from the fed female synganglion of the** ***Ixodes scapularis*** **transcriptome versus the conspecific species and other species**. Multiple sequence alignment (ClustalW) of the deduced amino acid sequence of a putative *I. scapularis* bursicon alpha (contig 8916) from sample Il-1 compared with the conspecific *I. scapularis* sequence (Iscap: XM_002407468), the American dog tick (Dvar: ACC99596) and the mosquito *Culex quingquefasciatus* (Culex: XM_001851995). Pairwise identity for contig 8916 versus *I. scapularis* is 71.2%; multiple alignment identity is 54.1%. Asterisks denote identical residues, dots indicate conservative substitutions.
(TIF)

Figure S6 **Multiple sequence alignment for eclosion hormone from the fed female synganglion of the** ***Ixodes scapularis*** **transcriptome versus the same and other species**. Multiple sequence alignment (ClustalW) of the deduced amino acid sequence of a putative *I. scapularis* eclosion hormone (contig16950) from the Illumina sample Il- 2 compared with the conspecific *I. scapularis* (Iscap: XP_002399271), the American dog tick (Dvar: ACC99595) and the mosquito *Aedes aegypti* (Aaegypt: XP_001661508). Pairwise identity for contig 16950 versus the *I. scapularis* sequence from Genbank versus is 97.7*%;* multiple alignment identity 48.6%. Asterisks denote identical residues, dots indicate conservative substitutions. Bold text indicates conserved disulfide bond domain (pfam4736).
(TIF)

Figure S7 **Multiple sequence alignment for precursor orcokinin5 from the fed female synganglion of the** ***Ixodes scapularis*** **transcriptome versus the same and other species**. Multiple sequence alignment (ClustalW) of the deduced amino acid sequence of a putative *I. scapularis* Orcokinin 5 peptide (contig 13501) from the Illumina sample Il-1 compared with the conspecific *I. scapularis* sequence (Iscap: XP_2401726) and the American dog tick sequence (Dvar: ACC99606). Pairwise identity for contig 13501 versus *I. scapularis* is 89.2*%;* versus *D. variabilis* 52.6%; multiple alignment identity 40.0%. Asterisks denote identical residues, dots indicate conservative substitutions.
(TIF)

Figure S8 **Multiple sequence alignment for proprotein convertase from the fed female synganglion of the** ***Ixodes scapularis*** **transcriptome versus the same and other species.** Multiple sequence alignment (ClustalW) of the deduced amino acid sequence of a putative *I. scapularis* Proprotein convertase (contig 23747) from the Illumina sample Il- 2 compared with the conspecific *I. scapularis* (Iscap: XM_2410491), the American dog tick (Dvar: ACD63025) and the marine worm, *Platynereis dumerilii* (Pdumer: E54439). Pairwise identity for contig 23747 versus the *I. scapularis* sequence from Genbank 100*%;* pairwise identity for contig 23747 versus *D. variabilis* sequence from Genbank 80.6%; multiple alignment identity (all 4 sequences) 51.1%. The contig sequence contains regions that correspond to the conserved peptidase domain. The S8 family has an Asp/His/Ser catalytic triad similar to but not identical to that found in trypsin-like proteases; Specific hit] pfam01483, Proprotein convertase P-domain; A unique feature of the eukaryotic subtilisin-like proprotein convertases is the presence of an additional highly conserved sequence of approximately 150 residues (P domain) located immediately downstream of the catalytic domain. Box shows the subtilase family, serine active site. Asterisks denote identical residues, dots indicate conservative substitutions.
(TIF)

Figure S9 **Multiple sequence alignment for allatostatin (prepro) from the fed female synganglion of the** ***Ixodes scapularis*** **transcriptome Illumina sample Il-1 versus the conspecific species and other species**. Multiple sequence alignment (ClustalW) of the deduced amino acid sequence of a putative *I. scapularis* allatostatin (contig 7636) compared with the conspecific *I. scapularis* (Iscap: XP_002416345) and the American dog tick (Dvar: ACC99603). Pairwise identity for contig 7636 versus *I. scapularis* sequence from Genbank versus is 95.8*%;* multiple alignment identity 55.5%. Asterisks denote identical residues, dots indicate conservative substitutions.
(TIF)

Figure S10 **Multiple sequence alignment for putative pyrokinin receptor from the fed female synganglion of the** ***Ixodes scapularis*** **transcriptome versus the same and other species**. Multiple sequence alignment (ClustalW) of the deduced amino acid sequence of a putative *I. scapularis* pyrokinin receptor (contig 570) from the Illumina sample Il- 2 compared with the conspecific *I. scapularis* (Iscap: XM_002401136), the American dog tick (Dvar: ACC99623). Pairwise identity contig 570 versus *I. scapularis* sequence from Genbank versus is 62.2*%;* multiple alignment identity 55.0%. Box shows the GPCR F1_1 domain (PS00237). The disulfide domain (from position 121–206), GPCR F1_2 was also found in all three sequences.
(TIF)

Figure S11 **Multiple sequence alignment of FMRFamide from the fed female synganglion of the** ***Ixodes scapularis*** **transcriptome Illumina sample Il-2 versus the same and other species.** Multiple sequence alignment with the translation of the nucleic acid sequence (contig 6861) versus an *I. scapularis* sequence from Genbank (Iscap: XM_002413792) and *Metaseiulus occidentalis* (Moccid: XP_003741665). Pairwise identity between contig 6861 and *I. scapularis* Genbank sequence is 62%; with the *M. occidentalis* sequence <1.0%.
(TIF)

Figure S12 **Multiple sequence alignment of the corazonin receptor from the fed female synganglion of the** ***Ixodes scapularis*** **transcriptome Illumina sample Il-2 versus the same and other species**. Multiple sequence alignment with the translation of the nucleic acid sequence (contig 8130) versus an *I. scapularis* sequence from Genbank (Iscap: XM_002402071) and *Anopheles gambiae* sequence (Agam: XM_321555). Pairwise identity for contig 8130 versus conspecific Genbank acquired sequence is 100%; pairwise identity between contig 8130, XM_002402071 and XM_321555 in three-way alignment is 42.1%. Bold-face sequences represent the GPCR family signature features (F1_2 from 131–206 domain) and F1-1 from 146–162 domain, bold italics, respectively).
(TIF)

Figure S13 **Multiple sequence alignment for calcitonin receptor from the fed female synganglion of the** ***Ixodes scapularis*** **transcriptome versus the same and other species.** Multiple sequence alignment (ClustalW) of the deduced amino acid sequence of a putative *I. scapularis* calcitonin receptor (contig 08281) from the Illumina sample Il- 2 compared with the conspecific *I. scapularis* (Iscap: XM_002408382) and *Rhipicephalus pulchellus* (Rpul: JAA63077). Pairwise identity contig 08281 versus *I. scapularis* sequence from Genbank 95.8*%;* multiple alignment identity 79.5%. Asterisks denote identical residues.
(TIF)

Figure S14 **Sequence alignment for sulfakinin (prepro) from the fed female synganglion of the** ***Ixodes scapularis*** **transcriptome sample Il-1 versus the same and other species**. Pairwise sequence alignment for sulfakinin (prepro) from the fed female synganglion of the *Ixodes scapularis* transcriptome versus the same from *Dermacentor variabilis*. Pairwise sequence alignment (ClustalW) of the deduced amino acid sequence of the putative *I. scapularis* preprosulfakinin (contig 30943) versus the same from *D. variabilis* (Dvar: ACC99604). Pairwise identity  = 78.3%. Asterisks denote identical residues.
(TIF)

Figure S15 **Multiple sequence alignment for myoinhibitory peptide from the fed female synganglion of the** ***Ixodes scapularis*** **transcriptome sample Il-1 versus the same and other species.** Multiple sequence alignment (ClustalW) of the deduced amino acid sequence of a putative *I. scapularis* myoinhibitory peptide (contig 38613) from the Illumina sample Il- 1 compared with the conspecific *I. scapularis* (Iscap: XP_002434041) and *Drosophila melanogaster* (Dmel: NP648971). Pairwise identity for contig 38613 versus the *I. scapularis* sequence from Genbank is 69.7*%;* multiple alignment identity 17.5%. Asterisks denote identical residues, dots indicate conservative substitutions.
(TIF)

Figure S16 **Sequence alignment (pairwise) for SIFamide peptide from the fed female synganglion of the** ***Ixodes scapularis*** **transcriptome sample Il-1 versus the conspecific species.** Pairwise sequence alignment (ClustalW) of the deduced amino acid sequence of the putative *I. scapularis* SIFamide (contig39081) compared with the conspecific *I. scapularis* sequence from Genbank (Iscap: XP_002414623) Pairwise identity  = 100% (excluding non-coding regions). Asterisks denote identical residues.
(TIF)

Figure S17 Multiple sequence alignment for insulin-like receptor from the fed female synganglion of the *Ixodes scapularis* transcriptome sample Il-2 versus the same and other species. Multiple sequence alignment (ClustalW) of the deduced amino acid sequence of a putative *I. scapularis* insulin receptor (contig13135) compared with the conspecific *I. scapularis* (Iscap: XM_002416179) and *Apis florea* (Apis: XR_143184). Pairwise identity for contig 13135 versus *I. scapularis* sequence from Genbank 98.2%*;* versus *Apis florea* sequence from Genbank, identity 47.5%. Asterisks denote identical residues, dots indicate conservative substitutions.
(TIF)

Figure S18 **Multiple sequence alignment for the sulfakinin receptor from the fed female synganglion of the** ***Ixodes scapularis*** **transcriptome sample Il-2 versus the conspecific species and other species**. Multiple sequence alignment (ClustalW) of the deduced amino acid sequence of a putative *I. scapularis* sulfakinin receptor (contig17295) compared with the conspecific *I. scapularis* (Iscap: XM_002413275) and *Culex quinquefasciatus* from Genbank (Culex: XM_001866703). Pairwise identity contig 17295 versus *I. scapularis* sequence from Genbank 51.2%*;* versus *C. quinquefasciatus* 44.2%. Asterisks denote identical residues, dots indicate conservative substitutions. The transmembrane domain (Pfam00001) is present.
(TIF)

Figure S19 **Pairwise sequence alignment for gonadotropin releasing hormone/AKH-like (GnRH) receptor from the fed female synganglion of the** ***Ixodes scapularis*** **transcriptome sample Il-2 versus the published sequence (GenBank) for this hormone of the same species.** Pairwise sequence alignment (ClustalW) of the deduced amino acid sequence of a putative *I. scapularis* GRH receptor (contig 12746) compared with the conspecific *I. scapularis* (Iscap: XM_002407293). Pairwise identity  = 68.1%. Asterisks denote identical residues.
(TIF)

Figure S20 **Multiple sequence alignment for the tachykinin receptor from the fed female synganglion of the** ***Ixodes scapularis*** **Il-2 versus the published sequences (GenBank) for this hormone for the same and other species**. Multiple sequence alignment (ClustalW) of the deduced amino acid sequence of a putative *I. scapularis* tachykinin receptor (contig 10361) compared with the conspecific *I. scapularis* GPCR receptor (XM_002411163) and *Sorex araneus* from Genbank (Sorex: XM_004612733). Pairwise sequence identity  = 99.9%; multiple sequence identity  = 59.9%. Asterisks denote identical residues, dots indicate conservative substitutions. This GPCR receptor includes the Serpentine type 7 TM GPCR chemoreceptor Srsx domain (Superfamily cl18179) characteristic of chemosensory functions.
(TIF)

Figure S21 **Sequence alignment (pairwise) for the neuropeptide F receptor from the fed female synganglion of the** ***Ixodes scapularis*** **transcriptome sample Il-2 versus the conspecific species**. Pairwise sequence alignment (ClustalW) of the deduced amino acid sequence of the putative *I. scapularis* neuropeptide F receptor (contig3925) compared with the conspecific *I. scapularis* (Iscap Genbank XP_002402168) Pairwise identity  = 100%. Asterisks denote identical residues. This receptor includes the serpentine type 7 TM GPCR chemoreceptor Srsx domain the (Super family cl18571) characteristic of chemosensory functions.
(TIF)

Figure S22 **Sequence alignment (pairwise) for the cardioacceleratory peptide (CCAP) receptor from the fed female synganglion of the** ***Ixodes scapularis*** **transcriptome sample Il-2 versus the conspecific species**. Pairwise sequence alignment (ClustalW) of the deduced amino acid sequence of the putative *I. scapularis* (CCAP) receptor (contig 19265) compared with the conspecific *I. scapularis* (Iscap: Genbank XP_002407935). Pairwise identity  = 52.4%. Pairwise identity for the coding region >90%. Asterisks denote identical residues. When compared with the sequence for *I. scapularis* (Genbank XP_002400846), both the GPCR domain F1.1 and F1.2 characteristic of these transmembrane receptors were identified.
(TIF)

Figure S23 **Sequence alignment (pairwise) for the ion binding peptide from the fed female synganglion of the** ***Ixodes scapularis*** **transcriptome sample Il-1 versus the conspecific species**. Pairwise sequence alignment (ClustalW) of the deduced amino acid sequence of the putative *I. scapularis* ion binding peptide (contig contig7332) compared with the conspecific *I. scapularis* sequence (Iscap Genbank XM_002399497). Pairwise identity  = 100.0%. Asterisks denote identical residues.
(TIF)

Figure S24 **Multiple sequence alignment for muscarinic acetylcholine receptor from the fed female synganglion of the** ***Ixodes scapularis*** **transcriptome Il-2 versus the conspecific species and other species**. Pairwise sequence alignment (ClustalW) of the deduced amino acid sequence of a putative *I. scapularis* muscarinic acetylcholine receptor (contig6186, Il-2) compared with the conspecific *I. scapularis* (Iscap: XP_0024003135) and the *Rhipicephalus microplus* (Rmicro: AFC88982). Pairwise identity  = 99.5%; multiple sequence identity 86.2%. Asterisks denote identical residues; dots indicate conserved residues.
(TIF)

Figure S25 **Sequence alignment (pairwise) for a putative dopamine receptor from the fed female synganglion of** ***Ixodes scapularis*** **transcriptome Il-2 versus the published sequences (GenBank) for this neurotransmitter for the conspecific species and other species.** Pairwise sequence alignment (ClustalW) of the deduced amino acid sequence of a putative octopamine receptor (contig 6007) compared with the conspecific *I. scapularis* dopamine receptor (XP_002408422). Pairwise sequence identity  = 93.4%. Asterisks denote identical residues. Dots indicate conserved residues.
(TIF)

Figure S26 **Multiple sequence alignment for metabotropic gamma aminobutyric acid (GABA) receptor from the fed female synganglion of the** ***Ixodes scapularis*** **transcriptome Il-2 versus the conspecific species and other species**. Pairwise sequence alignment (ClustalW) of the deduced amino acid sequence of a putative *I. scapularis* metabotropic GABA receptor (contig6166, Il-2) compared with the conspecific *I. scapularis* (Iscap: XM_002406043) and the *Rhipicephalus microplus* strain NRFS metabotropic GABA receptor, (Rmicro*:* JN974907). Pairwise identity  = 93.1%; multiple sequence identity 85.8%. Asterisks denote identical residues; dots indicate conserved residues.
(TIF)

Figure S27 **Sequence alignment (pairwise) for the glutamate (metabotropic) receptor from the fed female synganglion of the** ***Ixodes scapularis*** **transcriptome Il-1 versus the conspecific and other species.** Sequence alignment (ClustalW) of the deduced amino acid sequence of a putative *I. scapularis* glutamate (NMDA) receptor (contig 37672) from the Illumina sample Il- 1 compared with the conspecific *I. scapularis* (Iscap: XP_002413279). Pairwise identity contig 37672 versus *I. scapularis* sequence from Genbank versus 94.2%. Asterisks denote identical residues.
(TIF)

Figure S28 **Sequence alignment (pairwise) for the glutamate (ionotropic) receptor from the fed female synganglion of the** ***Ixodes scapularis*** **transcriptome sample Il-2 versus the published sequence (GenBank) for the conspecific species.** Pairwise sequence alignment (ClustalW) of the deduced amino acid sequence of the putative glutamate (ionotropic) receptor (contig 8353) compared with the *Ixodes scapularis* sequence (Iscap: XP_002407641). Pairwise identity  = 86.5%. Asterisks denote identical residues.
(TIF)

Figure S29 **Sequence alignment (pairwise) for the glutamate (NMDA) receptor from the fed female synganglion of the** ***Ixodes scapularis*** **transcriptome sample Il-1versus the conspecific species.** Pairwise sequence alignment (ClustalW) of the deduced amino acid sequence of the putative glutamate NDMA receptor (contig36704) compared with the *Ixodes scapularis* sequence (Iscap: XP_002408667). Pairwise identity  = 96.7%. Asterisks denote identical residues. The four transmembrane regions characteristic of the ionotropic and NMDA domains (pfam00060) as well as the N-terminal leucine/isoleucine/valine-binding protein (LIVBP)-like domain of the NMDA (cd06351) are present in this receptor (as well as other domains).
(TIF)

Figure S30 **Pairwise sequence alignment for the octopamine neurotransmitter receptor from the fed female synganglion of the** ***Ixodes scapularis*** **transcriptome (Il-2) versus published sequences (GenBank) from the conspecific species.** Pairwise sequence alignment (ClustalW) of the deduced amino acid sequence of a putative *I. scapularis* octopamine (contig 6007) from the Illumina sample Il-2 compared with the conspecific *I. scapularis* (Iscap: XP_002408422). Pairwise identity for contig 6007 versus the *I. scapularis* sequence from Genbank 93.4%. Asterisks denote identical residues. The 7-transmembrane receptor (rhodopsin family) is present in this receptor.
(TIF)

Figure S31 **Multiple sequence alignment for a putative serotonin receptor from the fed female synganglion of the** ***Ixodes scapularis*** **transcriptome (Il-2) versus published Genbank sequences from the conspecific and other species.** Multiple sequence alignment (ClustalW) of the deduced amino acid sequence of a putative *I. scapularis* serotonin receptor (contig 17069) from the Illumina sample Il-2 compared with the conspecific *I. scapularis* (Iscap: XP_0024050230) and the cattle tick *R. microplus* (Rmicro: AAQ89933) sequences. Pairwise identity for contig 17069 versus the *I. scapularis* sequence from Genbank  = 99.5*%;* versus *R. microplus* sequence from Genbank 91.4%%. Asterisks denote identical residues, dots indicate conservative substitutions. The 7-transmembrane receptor (rhodopsin family) is present in this receptor sequence.
(TIF)

Figure S32 **Sequence alignment (pairwise) for the ecdysone nuclear receptor from the fed female synganglion of the** ***Ixodes scapularis*** **transcriptome sample Il-2 versus the same for this species.** Pairwise sequence alignment (ClustalW) of the deduced amino acid sequence of the putative ecdysone nuclear receptor (contig 20681) compared with the *Ixodes scapularis* sequence (Iscap: XP_0024100149). Pairwise identity  = 100%. Asterisks denote identical residues. The DNA binding domain (cd07166) and ligand binding domain (cd06940) characteristic of nuclear receptors are present in this receptor sequence.
(TIF)

Figure S33 **Multiple sequence alignment for a putative spermidine receptor from the fed female synganglion of the** ***Ixodes scapularis*** **transcriptome (Il-2) versus published Genbank sequences from the conspecific and other species.** Multiple sequence alignment (ClustalW) of the deduced amino acid sequence (contig 6482) from the Illumina sample Il-2 compared with the conspecific *I. scapularis* sequence (Iscap: XP_002434346) and the yellow fever mosquito *Aedes aegypti* sequence (Aegypt: XP_001653177). Pairwise identity of contig 6482 versus *I. scapularis* sequence from Genbank  = 94.1*%;* versus *A.aegypti* sequence from Genbank  = 27.5%. Asterisks denote identical residues, dots indicate conservative substitutions. The Spermine/spermidine synthase domain (pfam01564) is highlighted in bold and enclosed in blocks (Spermine and spermidine are polyamines).
(TIF)

Figure S34 **RoeNoble.RL10_ Sample 454 Assembly**. Fasta file of the transcriptome assembly.
(FASTA)

Table S1 **Major gene categories in the** ***Ixodes scapularis*** **female synganglion.** Comparison of findings in the three different transcriptomes: 1  =  sample Il-1 (Illumina mixed unfed/fed/replete); 2  =  sample Il-2 (part-fed); 3  =  sample 454 (part-fed).
(DOCX)
