# Supplementary figures and images for: Transcriptome of the Female Synganglion of the Black-Legged Tick Ixodes scapularis (Acari: Ixodidae) with Comparison between Illumina and 454 Systems
Source: PLoS One. 2014 Jul 30;9(7):e102667. doi: 10.1371/journal.pone.0102667 (PMC4116169; doi:10.1371/journal.pone.0102667)

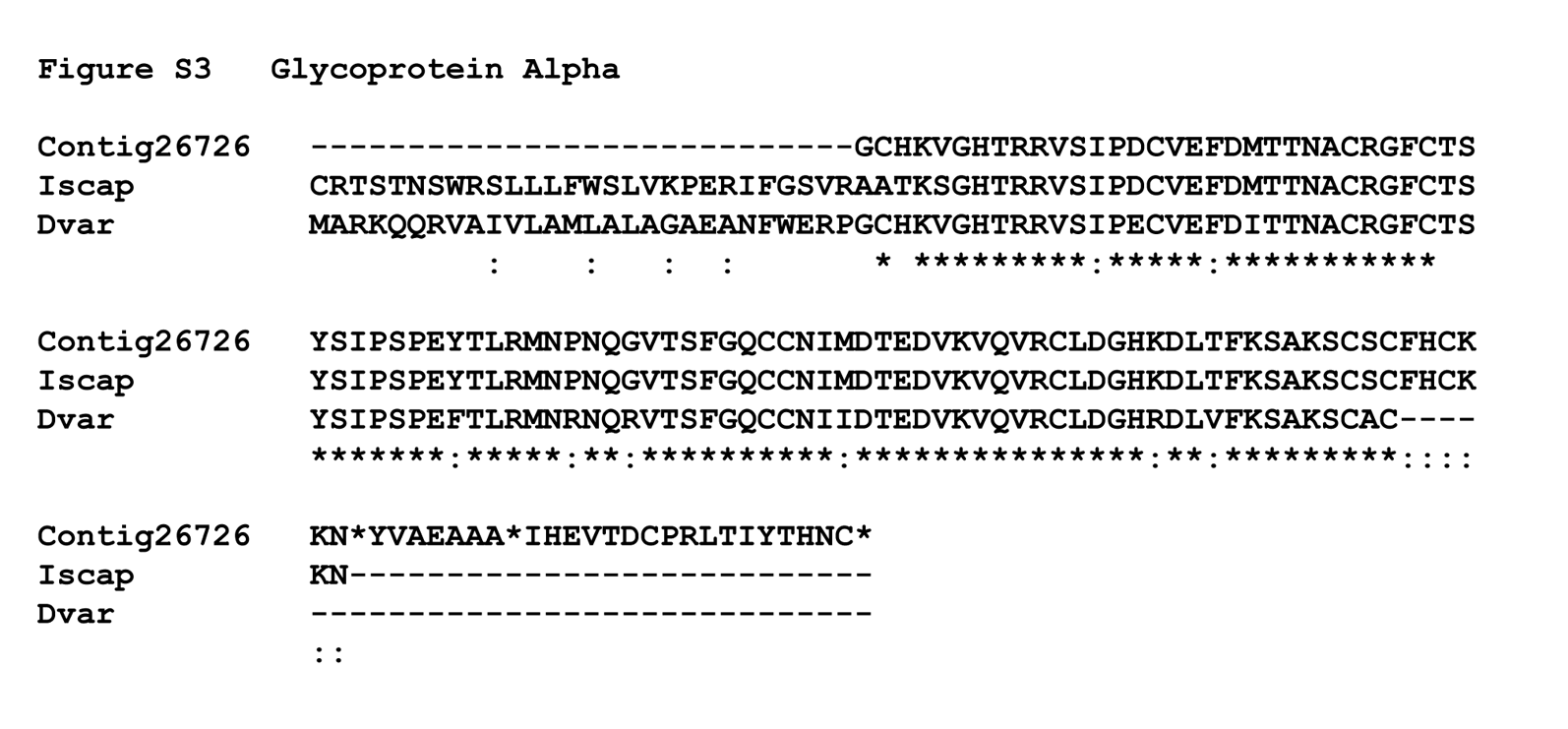

Supplement: Figure S3 — Multiple sequence alignment for Glycoprotein alpha from the fed female synganglion of the Ixodes scapularis transcriptome versus published sequences (GenBank) from the same the same and other species. Multiple sequence alignment (ClustalW) of the deduced amino acid sequence of a putative I. scapularis Glycoprotein alpha (contig 26726)from the Illumina sample Il-2 compared with the conspecific I. scapularis (Iscap: CAR94694) and the American dog tick (Dvar: ACC96601). Pairwise identity for contig 26726 versus the I. scapularis sequence from Genbank is 95.7%; multiple identity 83.2%. Asterisks denote identical residues, dots indicate conservative substitutions. (TIF) [file pone.0102667.s003.tif]

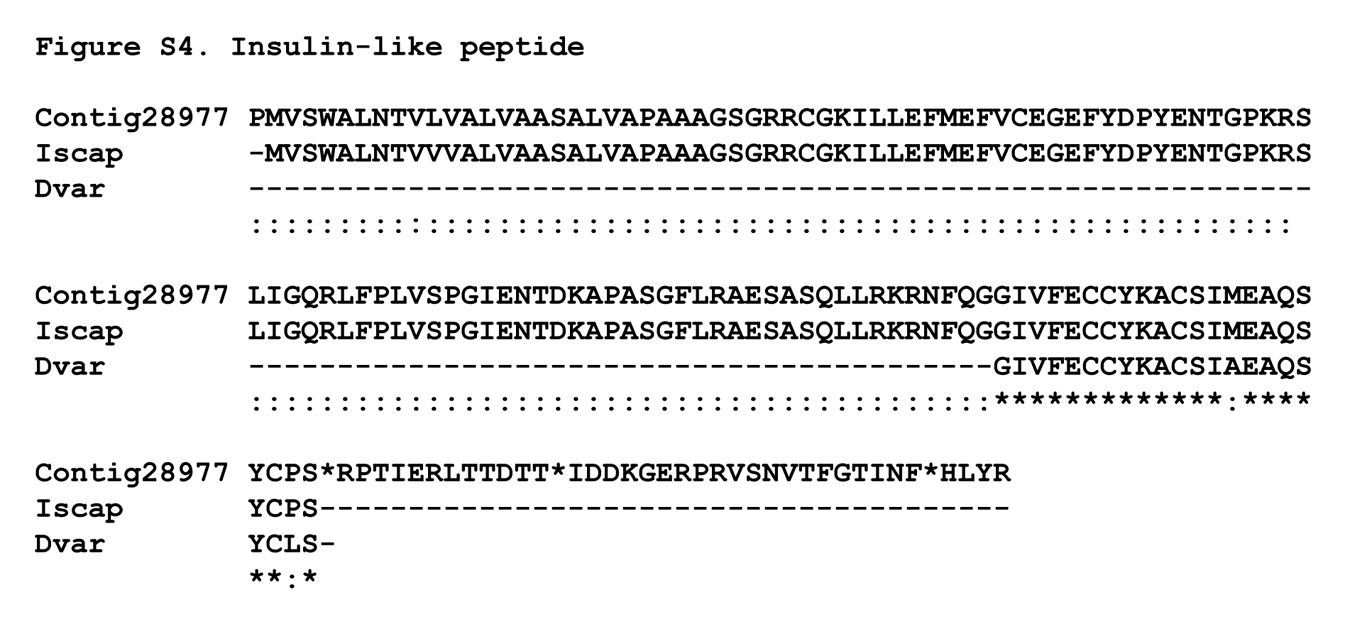

Supplement: Figure S4 — Multiple sequence alignment for Insulin-like peptide from the fed female synganglion of the Ixodes scapularis transcriptome versus the same and other species. Multiple sequence alignment (ClustalW) of the deduced amino acid sequence of a putative I. scapularis insulin-like peptide (contig 28977)from the Illumina sample Il-1 compared with the conspecific I. scapularis (Iscap: XM_002402930) and the American dog tick (Dvar: EU616823). Pairwise identity for contig 28977 versus the I. scapularis sequence from Genbank is 99.2%; multiple alignments with the I. scapularis and D. variabilis sequences are 93.9%. Asterisks denote identical residues, dots indicate conservative substitutions. Contains the I1GF-insulin-bombyxin-like superfamily conserved domain. Note the 4 cysteine residues characteristic of this peptide. Cys1 is linked by a disulfide bond to Cys3, Cys2 and Cys4 are linked by interchain disulfide bonds to cysteines in the "B" chain. (TIF) [file pone.0102667.s004.tif]

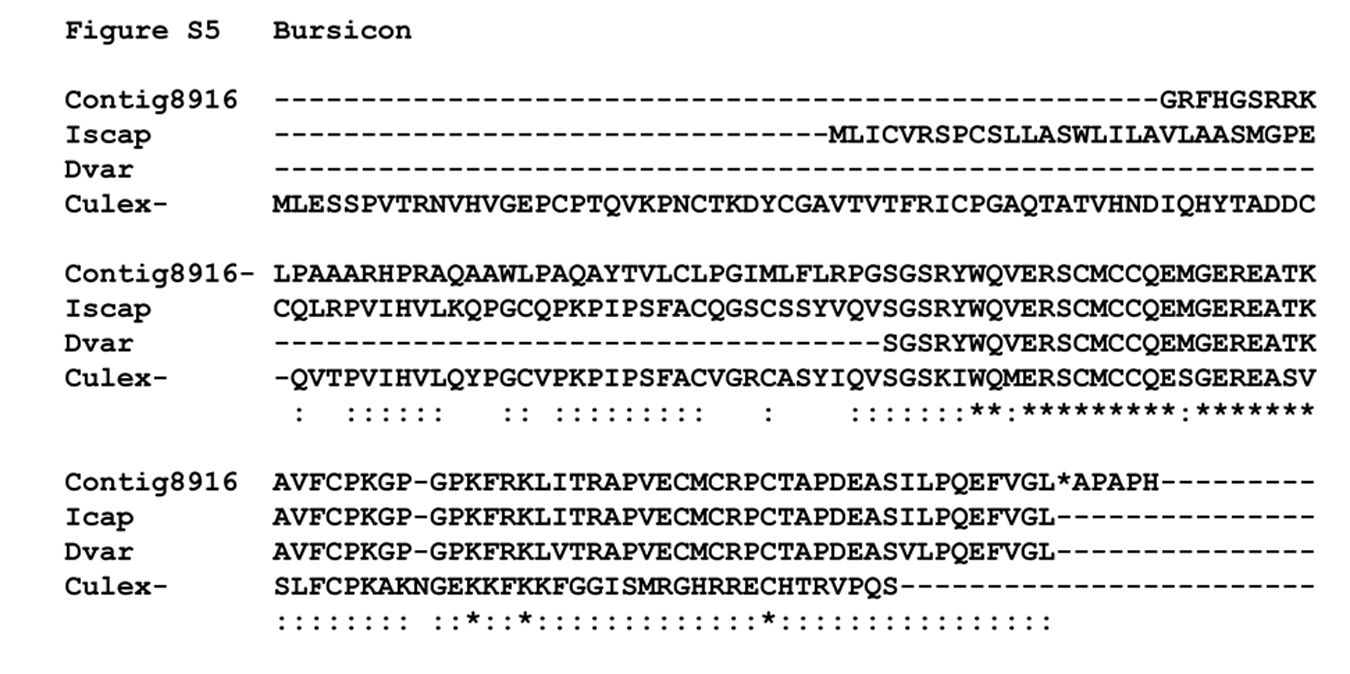

Supplement: Figure S5 — Multiple sequence alignment for bursicon alpha from the fed female synganglion of the Ixodes scapularis transcriptome versus the conspecific species and other species. Multiple sequence alignment (ClustalW) of the deduced amino acid sequence of a putative I. scapularis bursicon alpha (contig 8916) from sample Il-1 compared with the conspecific I. scapularis sequence (Iscap: XM_002407468), the American dog tick (Dvar: ACC99596) and the mosquito Culex quingquefasciatus (Culex: XM_001851995). Pairwise identity for contig 8916 versus I. scapularis is 71.2%; multiple alignment identity is 54.1%. Asterisks denote identical residues, dots indicate conservative substitutions. (TIF) [file pone.0102667.s005.tif]

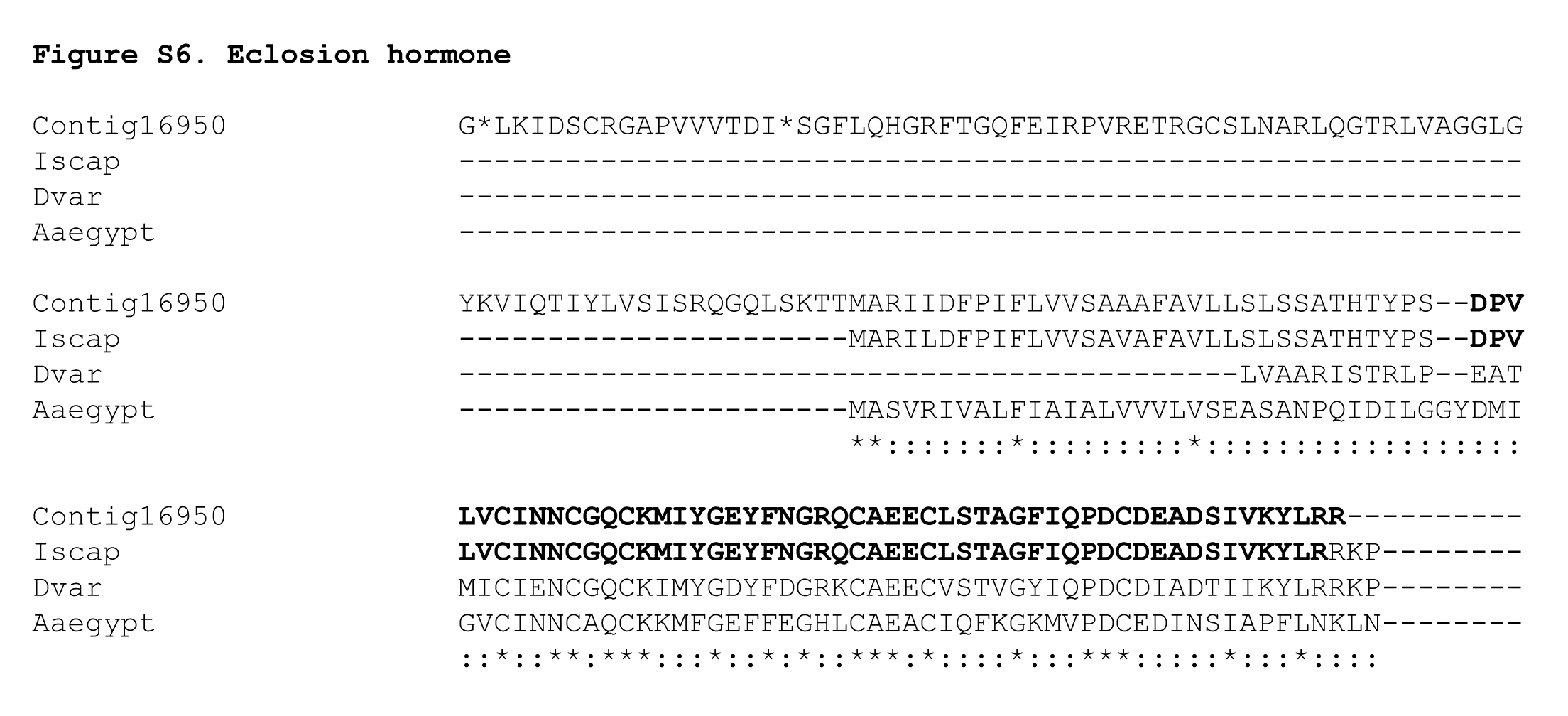

Supplement: Figure S6 — Multiple sequence alignment for eclosion hormone from the fed female synganglion of the Ixodes scapularis transcriptome versus the same and other species. Multiple sequence alignment (ClustalW) of the deduced amino acid sequence of a putative I. scapularis eclosion hormone (contig16950) from the Illumina sample Il- 2 compared with the conspecific I. scapularis (Iscap: XP_002399271), the American dog tick (Dvar: ACC99595) and the mosquito Aedes aegypti (Aaegypt: XP_001661508). Pairwise identity for contig 16950 versus the I. scapularis sequence from Genbank versus is 97.7%; multiple alignment identity 48.6%. Asterisks denote identical residues, dots indicate conservative substitutions. Bold text indicates conserved disulfide bond domain (pfam4736). (TIF) [file pone.0102667.s006.tif]

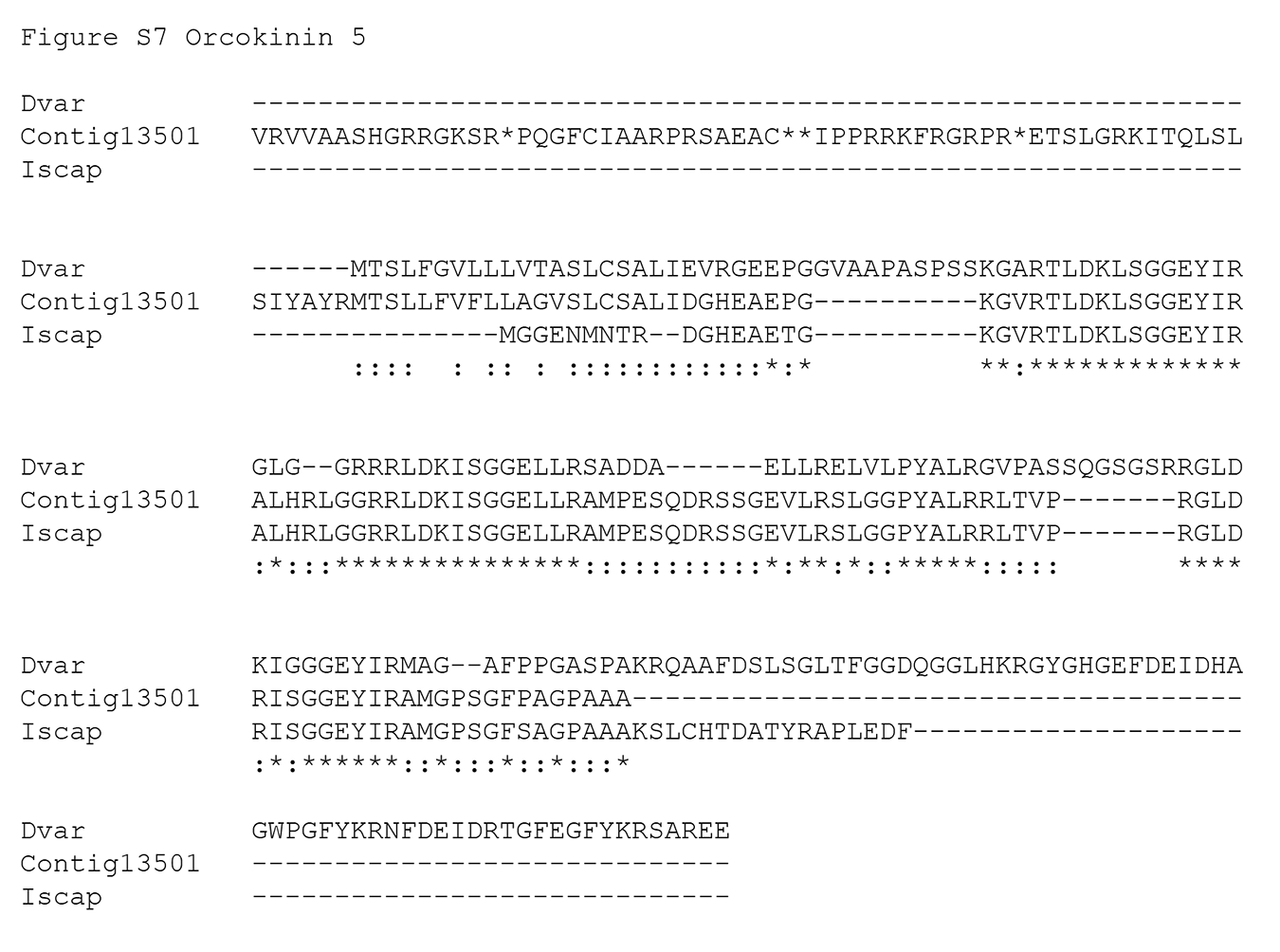

Supplement: Figure S7 — Multiple sequence alignment for precursor orcokinin5 from the fed female synganglion of the Ixodes scapularis transcriptome versus the same and other species. Multiple sequence alignment (ClustalW) of the deduced amino acid sequence of a putative I. scapularis Orcokinin 5 peptide (contig 13501) from the Illumina sample Il-1 compared with the conspecific I. scapularis sequence (Iscap: XP_2401726) and the American dog tick sequence (Dvar: ACC99606). Pairwise identity for contig 13501 versus I. scapularis is 89.2%; versus D. variabilis 52.6%; multiple alignment identity 40.0%. Asterisks denote identical residues, dots indicate conservative substitutions. (TIF) [file pone.0102667.s007.tif]

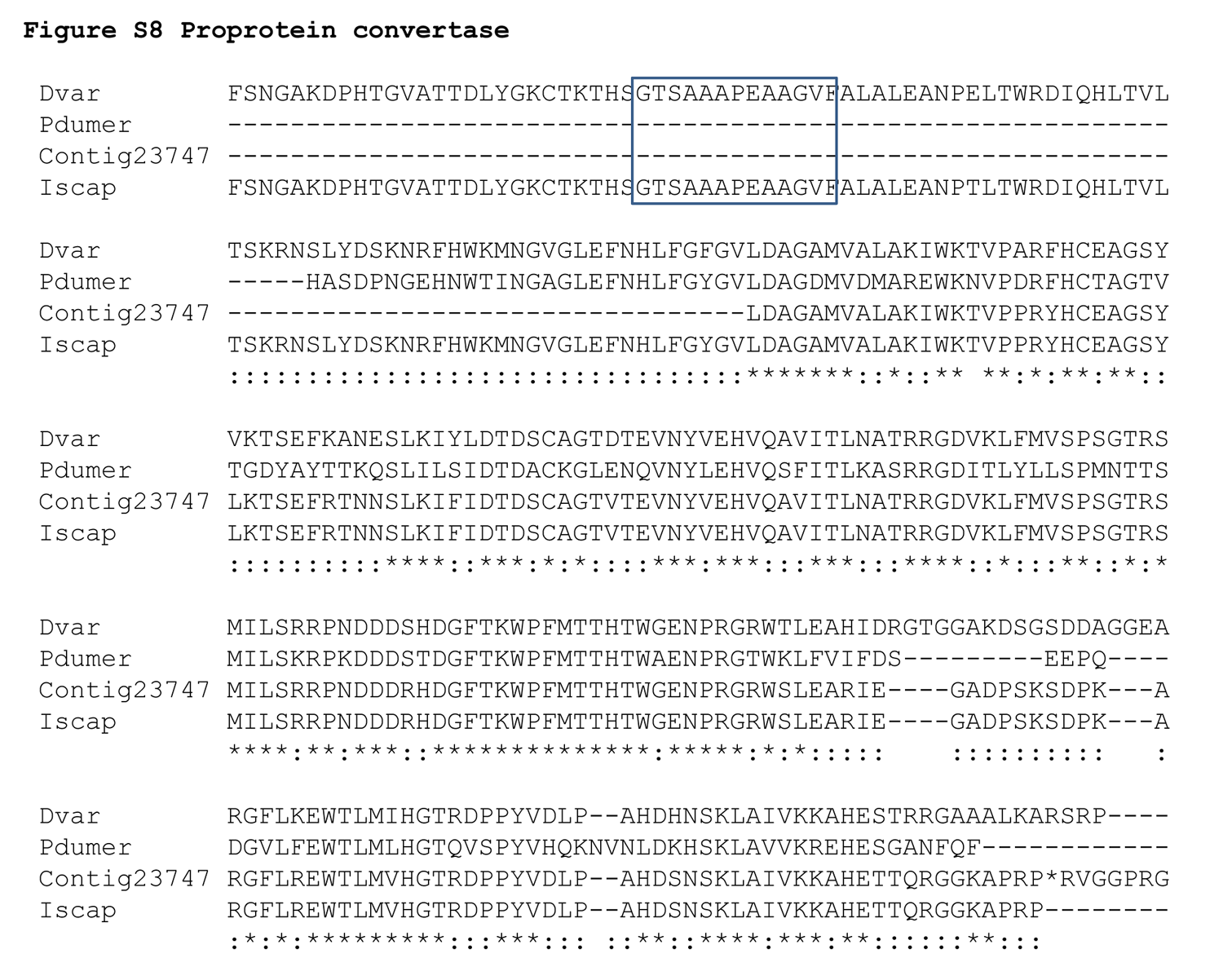

Supplement: Figure S8 — Multiple sequence alignment for proprotein convertase from the fed female synganglion of the Ixodes scapularis transcriptome versus the same and other species. Multiple sequence alignment (ClustalW) of the deduced amino acid sequence of a putative I. scapularis Proprotein convertase (contig 23747) from the Illumina sample Il- 2 compared with the conspecific I. scapularis (Iscap: XM_2410491), the American dog tick (Dvar: ACD63025) and the marine worm, Platynereis dumerilii (Pdumer: E54439). Pairwise identity for contig 23747 versus the I. scapularis sequence from Genbank 100%; pairwise identity for contig 23747 versus D. variabilis sequence from Genbank 80.6%; multiple alignment identity (all 4 sequences) 51.1%. The contig sequence contains regions that correspond to the conserved peptidase domain. The S8 family has an Asp/His/Ser catalytic triad similar to but not identical to that found in trypsin-like proteases; Specific hit] pfam01483, Proprotein convertase P-domain; A unique feature of the eukaryotic subtilisin-like proprotein convertases is the presence of an additional highly conserved sequence of approximately 150 residues (P domain) located immediately downstream of the catalytic domain. Box shows the subtilase family, serine active site. Asterisks denote identical residues, dots indicate conservative substitutions. (TIF) [file pone.0102667.s008.tif]

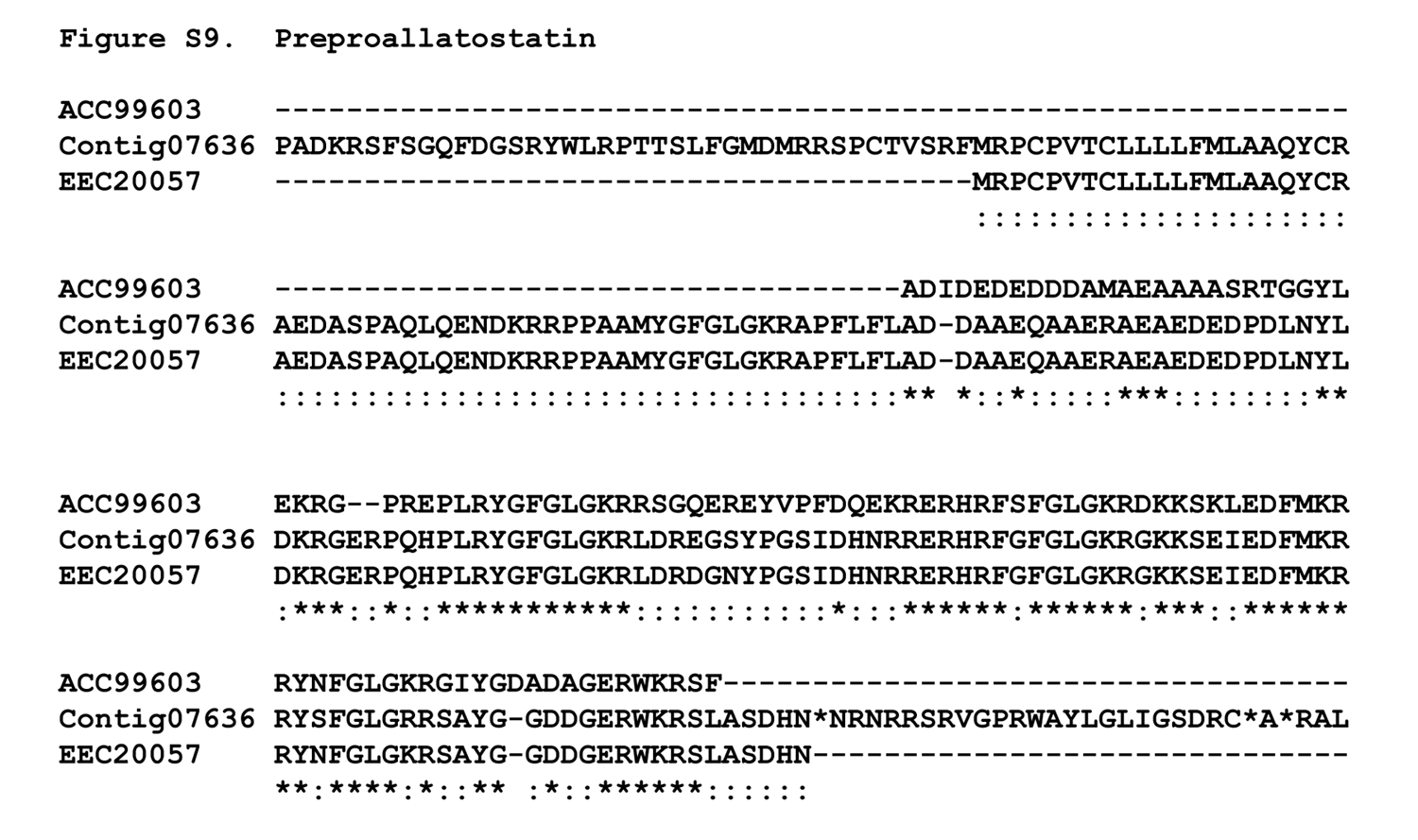

Supplement: Figure S9 — Multiple sequence alignment for allatostatin (prepro) from the fed female synganglion of the Ixodes scapularis transcriptome Illumina sample Il-1 versus the conspecific species and other species. Multiple sequence alignment (ClustalW) of the deduced amino acid sequence of a putative I. scapularis allatostatin (contig 7636) compared with the conspecific I. scapularis (Iscap: XP_002416345) and the American dog tick (Dvar: ACC99603). Pairwise identity for contig 7636 versus I. scapularis sequence from Genbank versus is 95.8%; multiple alignment identity 55.5%. Asterisks denote identical residues, dots indicate conservative substitutions. (TIF) [file pone.0102667.s009.tif]

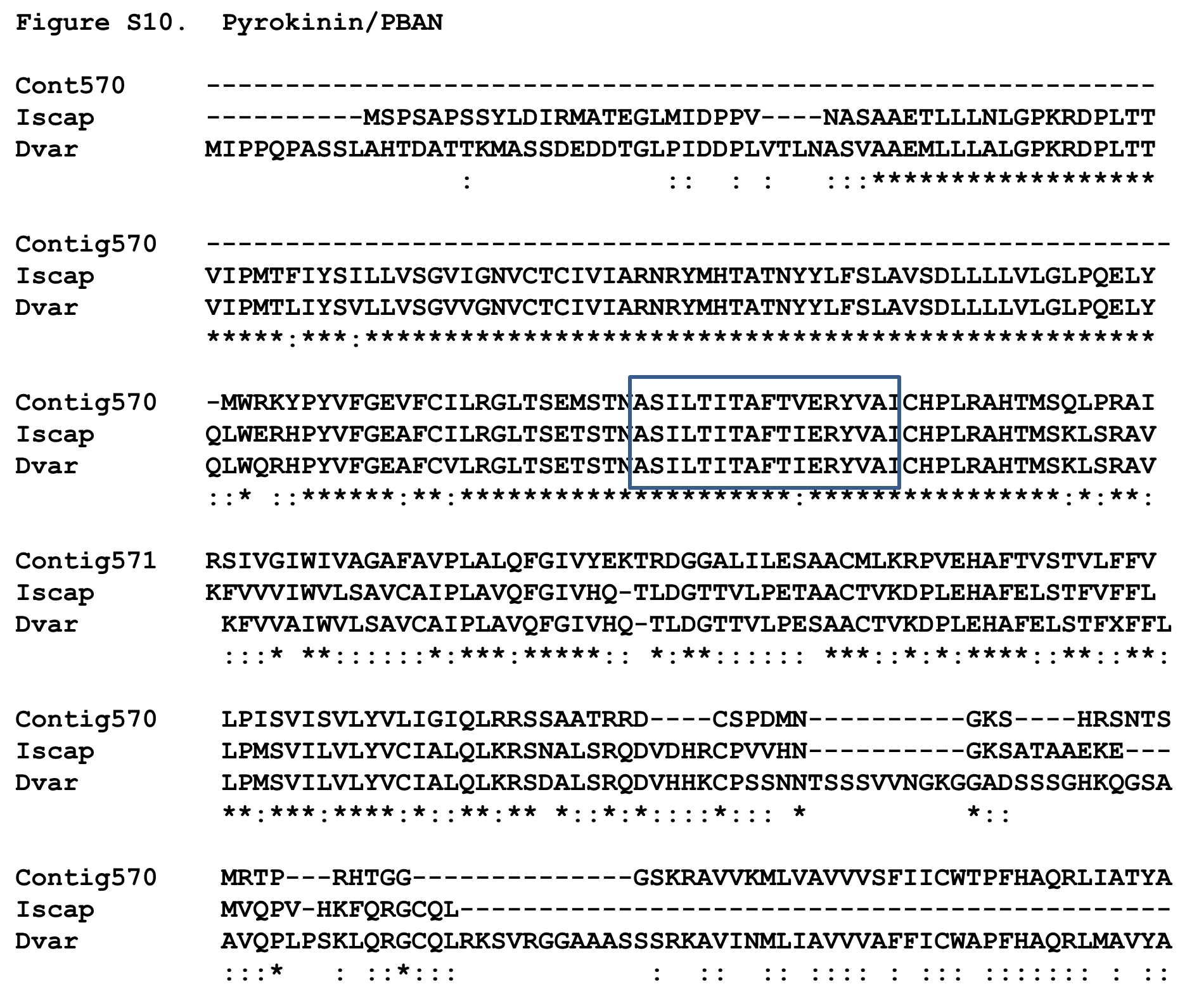

Supplement: Figure S10 — Multiple sequence alignment for putative pyrokinin receptor from the fed female synganglion of the Ixodes scapularis transcriptome versus the same and other species. Multiple sequence alignment (ClustalW) of the deduced amino acid sequence of a putative I. scapularis pyrokinin receptor (contig 570) from the Illumina sample Il- 2 compared with the conspecific I. scapularis (Iscap: XM_002401136), the American dog tick (Dvar: ACC99623). Pairwise identity contig 570 versus I. scapularis sequence from Genbank versus is 62.2%; multiple alignment identity 55.0%. Box shows the GPCR F1_1 domain (PS00237). The disulfide domain (from position 121–206), GPCR F1_2 was also found in all three sequences. (TIF) [file pone.0102667.s010.tif]

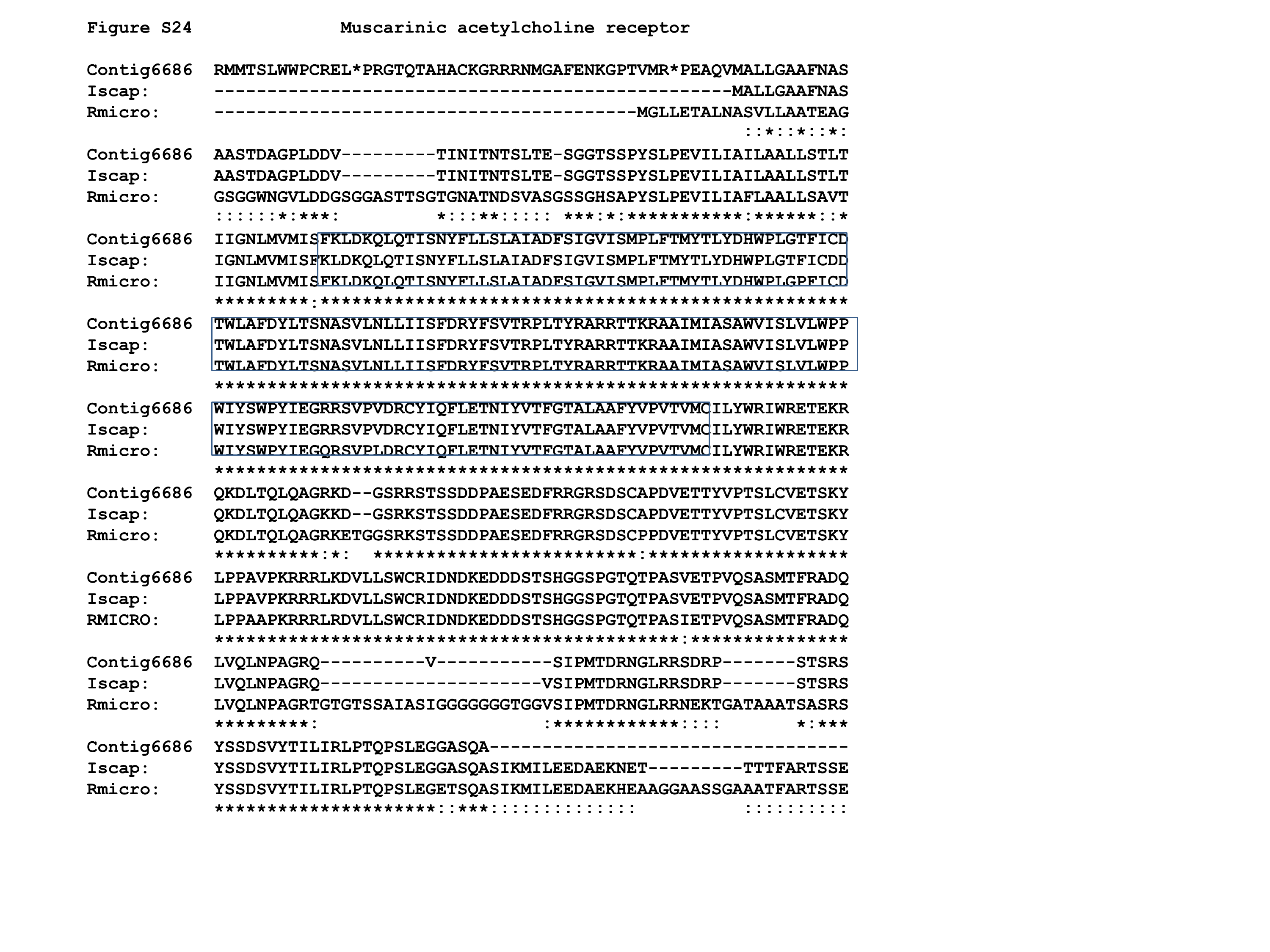

Supplement: Figure S24 — Multiple sequence alignment for muscarinic acetylcholine receptor from the fed female synganglion of the Ixodes scapularis transcriptome Il-2 versus the conspecific species and other species. Pairwise sequence alignment (ClustalW) of the deduced amino acid sequence of a putative I. scapularis muscarinic acetylcholine receptor (contig6186, Il-2) compared with the conspecific I. scapularis (Iscap: XP_0024003135) and the Rhipicephalus microplus (Rmicro: AFC88982). Pairwise identity = 99.5%; multiple sequence identity 86.2%. Asterisks denote identical residues; dots indicate conserved residues. (TIF) [file pone.0102667.s024.tif]

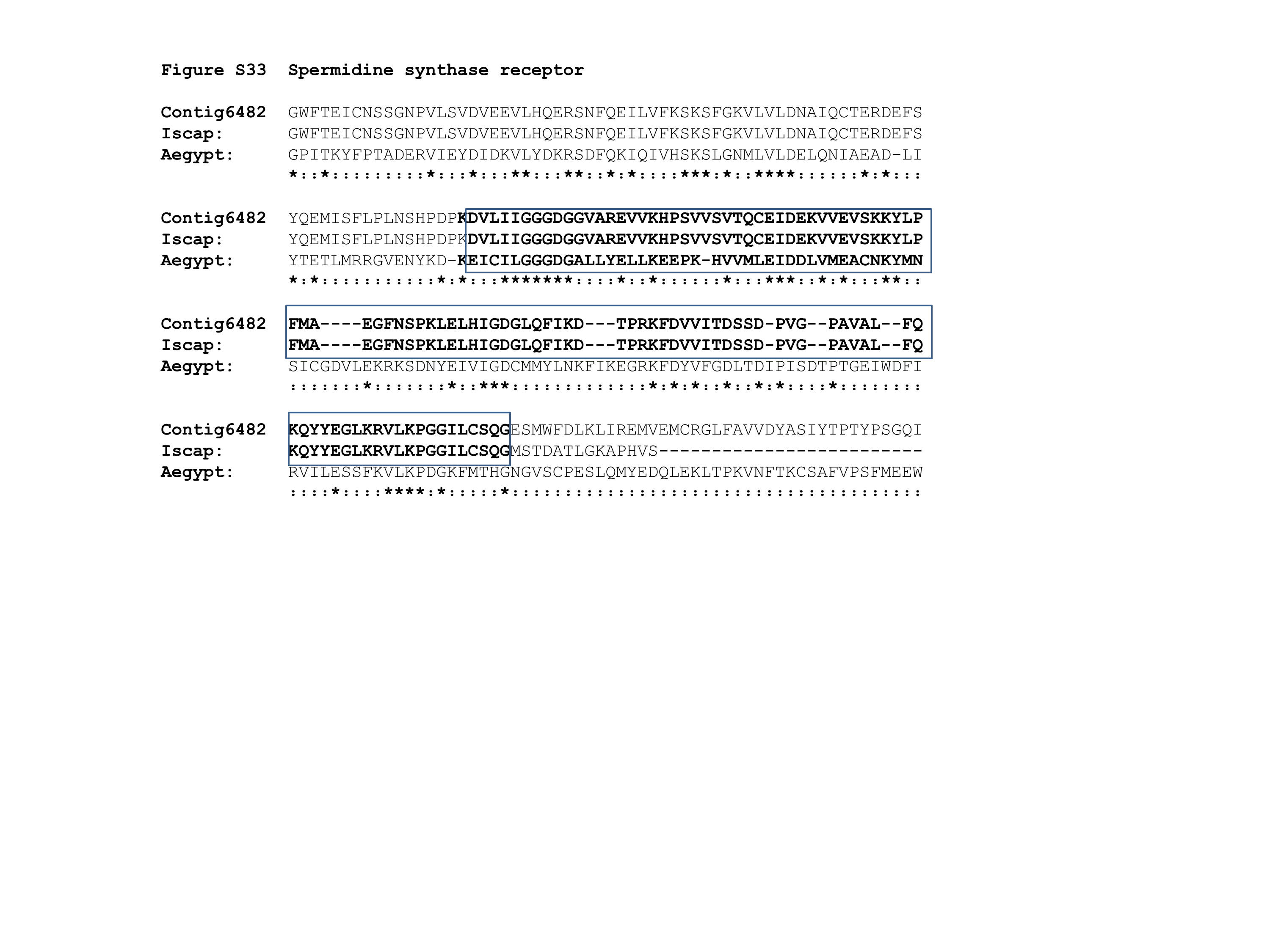

Supplement: Figure S33 — Multiple sequence alignment for a putative spermidine receptor from the fed female synganglion of the Ixodes scapularis transcriptome (Il-2) versus published Genbank sequences from the conspecific and other species. Multiple sequence alignment (ClustalW) of the deduced amino acid sequence (contig 6482) from the Illumina sample Il-2 compared with the conspecific I. scapularis sequence (Iscap: XP_002434346) and the yellow fever mosquito Aedes aegypti sequence (Aegypt: XP_001653177). Pairwise identity of contig 6482 versus I. scapularis sequence from Genbank = 94.1%; versus A.aegypti sequence from Genbank = 27.5%. Asterisks denote identical residues, dots indicate conservative substitutions. The Spermine/spermidine synthase domain (pfam01564) is highlighted in bold and enclosed in blocks (Spermine and spermidine are polyamines). (TIF) [file pone.0102667.s033.tif]
