# Supplementary material for: Transcriptome of the Female Synganglion of the Black-Legged Tick Ixodes scapularis (Acari: Ixodidae) with Comparison between Illumina and 454 Systems
Source: PLoS One. 2014 Jul 30;9(7):e102667. doi: 10.1371/journal.pone.0102667 (PMC4116169; doi:10.1371/journal.pone.0102667)
Supplement: Table S1 — Major gene categories in the Ixodes scapularis female synganglion. Comparison of findings in the three different transcriptomes: 1 = sample Il-1 (Illumina mixed unfed/fed/replete); 2 = sample Il-2 (part-fed); 3 = sample 454 (part-fed). (DOCX) [file pone.0102667.s035.docx]

Table S1. Major gene categories in the *Ixodes scapularis* female synganglion. Comparison of findings in the three different transcriptomes: 1 = sample Il-1 (Illumina mixed unfed/fed/replete); 2 = sample Il-2 (part-fed); 3 = sample 454 (part-fed)

___________________________________________________________________________________________________________________

**Gene category Size** Contig Gene Bank Species

**A. Neuropeptides** (GO:0005184)**^a^** (bp) No.^b^ E-value Accession No.^b^ (Top hit)^c^ Annotation

| Allatostatin preprohormone | 1513 | 1)07636  3)04922 | 0.0  3.00E-15 | XP_002416345  ACC99603 | *Ixodes scapularis*  *Dermacentor variabilis* | Bombystatin, myoinhibitory peptide |
| --- | --- | --- | --- | --- | --- | --- |
| Allatotropin | 847 | 2)08149  3)03453 | 3.76E-46  4.00E-46 | XP_002407036  XP_002407036 | *Ixodes scapularis*  *“ “* | In insects, stimulates CA to secrete JH; function in ticks unknown |
| Bursicon alpha | 362 | 1)8916 | 4.18E-64 | XP_002407512 | *Ixodes scapularis* | Cuticle plasticization/ tanning |
| Crustacean cardioactive peptide (CCAP) | 216  649 | 1)21311  2)22853 | 7.67E-10  1.47E-18 | XP_002176774  XP_002182735 | *Phaseodactylum tricornutum* | Phospholipid methyltransferase |
| Corticotropin-releasing factor-binding protein | 613  666  759 | 1)38695  2)07079  2)21180 | 7.6E-27  1.06E-06  1.49E-109 | XP_002410820  EFN70026  XP_002410820 | *Ixodes scapularis*  *Ixodes scapularis*  *“ “* | Transports CRF; Inhibits CRF |
| Diuretic hormone | 627 | 2)9802 | 5.02E-21 | XP_002435658 | *Ixodes scapularis* | Regulates water elimination (diuresis) similar to CRF |
| Eclosion hormone | 506 | 2)16950 | 1.52E-32 | XP_002399271 | *Ixodes scapularis* | Triggers ecdysis performance |
| FMRFamide | 1529 | 2) 6861 | 1.75E-105 | XP_002413837 | *Ixodes scapularis* | Stimulatory/inhibitory peptides |
| Glycoprotein hormone A | 548  365 | 1)13854  2)26726 | 2.0E-52  2.0E-139 | CAR94694  CAR94694 | *Ixodes scapularis*  *“ “* | Control aspects tick reproduction (Bissinger et al. (2011) |
| Insulin-like peptide/precursor | 491  769  559  1470 | 1)28977  1)37924  1)38625  2)12247 | 7.47E-53  1.10E-35  2.05E-40  6.00E-84 | XP_002402974  XP_002410559  XP_002413811  XP_002402974 | *Ixodes scapularis*  *“ “*  *“ “*  *“ “* | Regulates metabolism, stimulates ecdysteroidogenesis, controls growth and body size, reproduction |
| Insulin-like peptide/binding peptide | 612 | 2)17460 | 4.97E-55 | XP_002733803 | *Saccoglossus kowalesvskii* | Insulin-like inhibitor peptide |
| Ion transport peptide | 223  240  264  963  275  436  471 | 1)04754  1)04775  1)05422  1)07332  1)27396  2)04468  2)03084 | 2.54E-24  4.98E-41  2.42E-35  1.81E-151  5.88E-42  2.80E-71  2.51E-80 | XP_002435666  XP_002436129  XP_002414101  XP_002399539  XP_002434178  XP_557860  XP_002435666 | *Ixodes scapularis*  *“ “*  *“ “*  *“ “*  *“ “*  *Anopheles gambiae*  *“ “* | Organic anion transporter family. Controls water balance. |
| Myoinhibitory peptide precursor | 669 | 1)38613 | 2.18E-54 | XP_002434041 | *Ixodes scapularis* |  |
| Neurophysin/isotocin | 368  594 | 1)41001  2)03384 | 1.65E-20  8.84E-22 | P15211  P15211 | *Catostomus commersonii*  *“ “* | Transporter for oxytocin/vasopressin |
| Orcokinin 5 | 560 | 1)13501 | 5.37E-41 | XP_002401726 | *Ixodes scapularis* |  |
| Proprotein convertase | 215  2192  1389  508  530  772 | 1)20079  1)21646  1)21757  2)01125  2)04020  2)23747 | 5.14E-22  0.0  4.35E-28  3.69E-10  4.18E-88  5.67E-104 | XP_002435042  ACD63025  XP_002435648  XP_002939688  XP_002435648  XP_002410536 | *Ixodes scapularis*  *Dermacentor variabilis*  *Ixodes scapularis*  *Xenopus tropicalis*  *Ixodes scapularis*  *“ “* | Conversion of proteins to active form; post-translation modification. |
| Preprosulfakinin | 333  337 | 1)30943  2)23345 | 2.40E-27  2.43E-27 | ACC99604  ACC99604 | *Dermacentor variabilis*  *Dermacentor variabilis* |  |
| SIF precursor | 514 | 1)39081 | 5.65E-30 | XP_002414623 | *Ixodes scapularis* | Regulate sexual behavior |

**Gene Category**

**A. Neuropeptides** (continued) **^a^** Size Contig Gene Bank Species

**Receptors** GO:0008188 (bp) No. E-value^b^ Accession No.^b^ (Top hit)^c^ Annotation/function

| Adipokinetic hormone receptor | 64 | 3)03547 | 4.6 | ABD60146 | *Anopheles gambiae* |  |
| --- | --- | --- | --- | --- | --- | --- |
| Allatostatin | 223  670  212  433  535  982  423  496 | 1)18698  1)14652  1)19248  2)01184  2)10565  2)19456  2)26869  2)30445 | 4.46E-37  5.03E-126  7.18E-32  3.86E-57  2.27E-99  2.75E-142  1.65E-55  3.63E-47 | XP_002414997  XP_002403852  XP_002414997  XP_002433373  XP_002403852  XP_002414997  XP_002433372  XP_002433372 | *Ixodes scapularis*    *“ “*  *“ “*  *“ “* | Inhibit JH, myoinhibitory  “ “ |
| Calcitonin  “  “  “  “ | 833  1408  761  100 | 1)8150  1)8150  1)11300  1)18334  1)26346  3)08030  3)08281 | 1.67E-75  1.53E-84  1.23E-87  6.71E-46  4.50E-60  1.00E-11  1.53E-84 | XP_002414039 XP_002408426  XP_002414039  XP_002402486  XP_002436229  XP_002739426  XP_002408426 | *Ixodes scapularis*  *“ “*  *“ “*  *“ “*  *“ “*  *Saccoglossus kowalevskii*  *Ixodes scapularis* | Secretin type hormone receptor (7-TM 2 superfamily)  “  “  “ |
| Cardioacceleratory protein | 265  286  735  974 | 1)17216  1)32768  1)37595  2)19265 | 1.02E-09  2.37E-46  8.22E-70  6.74E-109 | XP_002407935  XP_002400846  NP_001076795  “ | *Ixodes scapularis*  *“ “*  *Tribolium castaneum*  *“ “* |  |
| Corazonin | 310  1774 | 1)20361  2)08130 | 1.81E-51  3.18e-85 | XP_002435340  XP_002435340 | *Ixodes scapularis*  *“ “* | Regulates cardiac activity |
| Corticotropin-releasing factor | 1058  541  321  759 | 2) 799  2) 1541  2) 16533  2) 21180 | 2.48E-91  3.10E-83  3.56E-42  1.49E-109 | XP_002434070  XP_002403968  XP_002406301  XP_002410820 | *“ “*  *“ “*  *“ “*  *“ “* | Diuretic hormone receptor activity |
| Eclosion hormone | 506 | 2)16950 | 1.50E-32 | XP_002399271 | *Ixodes scapularis* | Triggers ecdysis |
| Gonadotropin releasing hormone ii receptor | 310  469 | 1)20361  2)12746 | 1.81E-51  2.56E-16 | XP_002435340  XP_002407337 | *Ixodes scapularis*  *“ “* | 7 Transmembrane receptor (rhodopsin) |
| Insulin receptor | 200  2070  313  492  976  2363  1170 | 1)27807  1)23024  1)20054  1)17037  2)07890  2)09150  2)10166 | 1.24E-31  4.66E-67  1.58E-50  6.48E-65  0.00  0.00  1.08E-138 | XP_002416224  XP_002400683  XP_002413130  XP_002400684  XP_002416224  XP_002400684  XP_002413130 | *Ixodes scapularis*  *“ “*  *“ “*  *“ “*  *“ “*  *“ “*  *“ “* | Regulates metabolism, controls growth and body size. |
| Neuropeptide Y | 317  409 | 2)03925  2)05327 | 3.34E-56  1.29E-36 | XP_002402168  XP_002413319 | *Ixodes scapularis*  *“ “* | 7 Tm-4 superfamily) Analogous neuropeptide F in *Drosophila* |
| Perisulfakinin | 218  618  37 | 1)00982  2)13955  3)05840 | 4.95E-25  4.62E-56  6.0 | AAX56942  XP_002404195  AAX56942 | *Periplaneta americana*  *Ixodes scapularis Periplaneta americana* | 7 Transmembrane receptor  (rhodopsin family) GPCR |
| Proctolin | 122 | 3)07349 | 3.00E-35 | EFX81804 | *Daphnia pulex* | Neuromodulator of muscle contractions (Lange 2002) |
| Pyrokinin | 642  816  674 | 2)00570  2)08246  2)10989 | 1.35E-56  7.72E-86  5.43E-75 | ACC99623  XP_002709778  XP_002401180 | *Dermacentor variabilis*  *Ixodes scapularis*  *“ “* | 7 Transmembrane receptor GPCR; PBAN |
| SIFamide | 221  334 | 1)03255  2)0280 | 9.32E-35  2.07E-26 | XP_002406272  XP_001947491 | *Ixodes scapularis*  *“ “* |  |
| Sulfakinin | 590 | 2)17295 | 5.53E-45 | XP_001866738 | *Culex quinquefasicatus* | Transmembrane olfactory receptor GPCR |
| Tachykinin | 413 | 2)02906  3)00817 | 6.44E-12  9.00E-64 | XP_002735999  XP_001607956 | *Saccoglossus kowalevskii*  *Nasonia vitripennis* | Transmembrane olfactory  receptor (7 Tm-4 superfamily) |

**Gene Category**

**B. Neurohormones/**

**Neurotransmitters receptors and transporters**

Peptides GO:0006576

| Acetylcholine | 2126  1443 | 1)37062  1)06081 | 0.00  0.00 | XP_002409079  XP_002406474 | *Ixodes scapularis*  *“ “* |  |
| --- | --- | --- | --- | --- | --- | --- |
| Acetylcholinesterase | 263  368  1009  2131  536  399  369  311  331  1553  636  800  2857  347  390  472  414  525  403  793  329  338  74  224  126  87  178  134  271  282  111  293 | 1)02142  1)02236  1)22170  1)22310  1)38206  2)02895  2)04828  2)05417  2)05606  2)07246  2)09198  2)11833  2)13505  2)19031  2)19111  2)20560  2)24033  2)25204  2)27953  2)28368  2)28565  2)29612  3)01075  3)01510  3)01873  3)02217  3)02674  3)03215  3)03858  3)04618  3)07043  3)15679 | 1.68E-44  4.55E-55  8.23E-142  0.00  3.89E-86  2.04E-05  2.31E-57  4.69E-55  7.84E-42  0.0  2.93E-56  3.40E-115  0.0  8.56E-28  3.64E-15  6.59E-81  8.79E-78  3.31E-55  4.38E-08  8.44E-151  1.65E-39  6.40E-36  3.00E-06  3.00E-73  2.00E-73  3.00E-41  3.00E-52  5.00E-74  1.00E-98  1.00E-158  4.00E-49  4.00E-127 | XP_002406790  XP_002410153  XP_002410152  XP_002402744  XP_002414017  XP_002402745  XP_002404877  XP_002410116  XP_002434456  XP_002399759  XP_002402741  XP_002402739  XP_002413212  XP_002414016  XP_002404385  XP_002410153  XP_002403057  XP_002410153  XP_002413764  XP_002406790  XP_002410153  XP_002400147  XP_002416389  XP002415881  XP_002410152  XP_002436252  XP_002409707  XP_002401227  XP_002411692  XP_002406790  XP_002403057  XP_002410151 | *Ixodes scapularis*  *“ “*  *“ “*  *“ “*  *“ “*  *“ “*  *“ “*  *“ “*  *“ “*  *“ “*  *“ “*  *“ “*  *“ “*  *“ “*  *“ “*    *“ “*  *“ “*  *“ “*  *“ “*  *“ “*  *“ “*  *“ “*  *“ “* | Catalyzes acetylcholine, terminate neurotransmission (Superfamily cl12031, Esterases) |
| γ-aminobutyric acid (GABA) | 1138  494  842  1012  479  136  140  181 | 1)11392  1)13736  2)00515  2)28199  2)18816  3)07822  3)15171  3)20080 | 4.96E-85  1.49E-77  1.49E-89  1.27E-81  5.37E-67  3.00E-38  2.00E-96  1.00E-102 | XP_002434689  XP_002410380  EFN70737  XP_002434689  ACV07675  XP_002411566  XP_001607062  XP_002411566 | *Ixodes scapularis*  *“ “*  *“ “*  *Ixodes scapularis*  *R. microplus*  *Ixodes scapularis*  *“ “*  *“ “* | Neurotransmitter gated ion channel. Transiently opens anion-selective ion channels; inhibits uptake neurotransmitters |
| GABA transporter | 437  363  1296  2540  1306  2939  1098  532  2130  1114  442  2273  434  2197  863  441  462  145  140  39 | 1)01005  1)10674  1)07340  1)21887  1)24667  1)25423  1)36575  1)38627  2)05880  2)07525  2)22218  2)12024  2)14056  2)14179  2)19597  3) 00537  3)04386  3)09663  3)15171  3)16240 | 6.96E-19  2.36E-67  1.02E-100  0.00  9.87E-136  0.00  0.00  7.07E-82  0.00  1.82E-156  1.49E-72  0.00  8.02E-71  0.00  1.57E-140  6.00E-122  2.00E-126  5.00E-63  1.00E-97  2.00E-13 | XP_002411212  XP_002406618  XP_002405499  XP_002405708  XP_002413104  XP_002433531  XP_002406618  XP_002413104  XP_002413104  XP_002405499  XP_002405706  XP_002405708  XP_002411212  XP_002406618  XP_002433531  XP_002405706  XP_002405708  XP_002413104  XP_002406618  XP_002405499 | *Ixodes scapularis*  *“ “*  *“ “*  *“ “*  *“ “*  *“ “*  *“ “*  *“ “*  *“ “*  *“ “*  *“ “*  *“ “*  *“ “*  *“ “*  *“ “*  *“ “*  *“ “*  *“ “*  *“ “*  *“ “* | Transports GABA |
| Dopamine d1 | 257  476  438 | 1)17334  1)37976  2)18535 | 1.58E-42  3.53E-87  2.12E-31 | XP_002435581  XP_002409287  XP_002425605 | *Ixodes scapularis*  *“ “*  *Pediculus humanus corporis* |  |
| Glutamate synthase | 833  273  529  273  322  330  2549  1303  887  114 | 1)07033  1)13366  1)20548  1)25528  1)27749  1)37376  2)06408  2)08625  2)27837  3)00121 | 3.26E-124  1.15E-21  9.97E-52  2.16E-164  1.20E-34  0.00  0.00  0.00  1.32E-121  2.00E-60 | XP_316385  XP_002402057  EFN72550  XP_002408118  AAV31916  XP_002435411  EFN72550  XP_002408118  XP_316385  XP_002435411 | *Anopheles gambiae*  *Ixodes scapularis*  *Camponotus floridanus*  *Ixodes scapularis*  *Aedes aegypti*  *Ixodes scapularis*  *Camponotus floridanus*  *Ixodes scapularis*  *Anopheles gambiae*  *Ixodes scapularis* | Flavoprotein catalyzes synthesis of L-glutamate, releasing active neurotransmitter. |
| Noradrenalin/norepinephrine | 437  1002 | 1)01005  2)19932 | 6.96E-19  5.00E-54 | XP_002411212  XP_002411212 | *Ixodes scapularis*  *“ “* |  |
| Octopamine | 399  622  280  928  515  467  306  2169  673  472  1352 | 1)00252  1)16101  1)18983  1)24237  2)01746  2)02831  2)05029  2)06007  2)07981  2)11022  2)12937 | 2.84E-60  5.78E-67  7.50E-24  2.40E-129  8.50E-58  6.05E-58  8.31E-52  2.04E-169  3.75E-92  2.61E-45  2.55E-174 | XP_002411135  XP_002405020  XP_002401109  XP_00241113  XP_004011135  XP_002408812  XP_002405020  XP_002408422  XP_002401109  XP_002406095  XP_002400568 | *Ixodes scapularis*  *“ “*  *“ “*  *“ “*  *“ “*  *“ “*  *“ “*  *“ “*  *“ “*  *“ “*  *“ “* |  |
| Serotonin | 235 | 1)00587 | 7.03E-27 | XP_002404998 | *Ixodes scapularis* |  |
| Na+- neurotransmitter symporter | 229  571  2149  2533  1150  664 | 1)05982  1)10483  1)21548  2)06259  2)22467  2)23558 | 5.58E-40  6.04E-99  0.00  0.0  6.43E-120  1.09E-96 | XP_002415786  XP_002414864  XP_002415798  XP_002415786  XP_002415798  XP_002414864 | *Ixodes scapularis*  *“ “*  *“ “*  *“ “*  *“ “*  *“ “* | Catalyze uptake neurotransmitters at synapse |

**Gene Category**

**B. Neurohormones/**

**Neurotransmitters** (continued)

Receptors (GO:0045213)

| Acetylcholine (muscarinic/nicotinic) | 1443  455  246  677  285  1447  510  720  336  2126  472  1808  320  922  1258  619  645  306  042  716  374  837  91  408  316  130 | 1)06081  1)09892  1)11163  1)13629  1)16104  1)21524  1)28247  1)24448  1)26324  1)37062  2)02504  2)06187  2)11787  2)17503  2)20736  2)21709  2)21504  2)23252  2)27432  2)27668  2)28247  2)28680  3)03753  3)11444  3)18084  3)19165 | 0.00  3.79E-65  2.30E-38  5.54E-96  1.13E-40  1.60E-113  1.47E-46  2.01E-41  4.47E-50  0.00  3.00E-25  1.46E-166  2.02E-53  1.04E-47  1.47E-46  0.00  8.26E-20  1.79E-40  8.83E-118  2.98E-61  1.66E-47  1.61E-41  3.00E-57  5.00E-66  4.00E-24  2.00E-145 | XP_002406474  XP_001945224  XP_002405720  XP_002408268  XP_002428480  NP_001103387  XP_002403135  ACT35385  XP_001848873  XP_002409079  NP_001155994  ABV72689  XP_002405720  ACY82688  XP_002403135  XP_002409079  ADG63461  XP_002408268  ACZ37230  XP_002406474  XP_002407301  ACT35385  XP_002409079  AAQ62631  XP_002408268  XP_002405720 | *I. scapularis*  *Acyrthosiphon pisum*  *Ixodes scapularis*  *“ “*  *P. humanus*  *B. mori*  *“ “*  *Pardosa pseudoannulata*  *Culex quinquefasciatus*  *Ixodes scapularis*  *Tribolium castaneum*  *Bombyx mori*  *I. scapularis*  *Nasonia Vitripennis*  *“ “*  *Ixodes scapularis*  *Pardosa pseudoannulata*  *Ixodes scapularis*  *A. Suum*  *I. scapularis*  *“ “*  *Pardosa pseudoannulata*  *I. scapularis*  “ “  *Takifugu rubripes*  *I. scapularis* | Neurotransmitter-gated ion-channel ligand binding domain |
| --- | --- | --- | --- | --- | --- | --- |
| γ-aminobutyric acid | 306  1454  842  531  1012 | 1)04210  1)13504  2)00515  2)27756  2)28199 | 4.95E-49  6.40E-62  1.49E-87  4.87E-46  1.27E-81 | XP_002415877  XP_002408370  EFN70737  XP_002434013  XP_002434689 | *Ixodes scapularis*  *“ “*  *Campanotus floridanus*  *Ixodes scapularis*  *“ “* | Inhibits neurotransmission.  Neurotransmitter-gated ion-channel transmembrane region Family includes the four transmembrane helices that form the ion channel. |
| Dopamine (D1, D2, D4 or uncharacterized) | 257  860  798  508  985  2130  270  205 | 1)17334  2)00433  2)01474  2)15135  2)19256  2)27487  3)05350  3)06706 | 1.58E-42  1.15E-50  8.38E-106  1.60E-21  1.27E-70  0.0  3.00E-37  7.00E-98 | XP_002435581  XP_002416450  XP_002415734  XP_002435581  FA02832  XP_002409287  NP_001136143  XP_002409287 | *Ixodes scapularis*  *“ “*  *“ “*  *“ “*  *Tribolium castaneum*  *Ixodes scapularis*  *Gallus gallus*  *Ixodes scapularis* | 7 Transmembrane receptor (rhodopsin family) |
| Glutamate | 236  330  365  237  326  316  256  205  1895  374  388  586  361  1311  1462  1769  1743  474  493  1037  1886  37  1440  3261  600  1238  2192  957  2356  1391  704  1302  1075  677  2581  1557  417  858  670  779  383  527  758  1047  378  101  128  156  420  260 | 1)00375  1)01267  1)01300  1)01720  1)03957  1)04554  1)05474  1)05754  1)06224  1)08144  1)08566  1)10162  1)15826  1)21754  1)21808  1)21909  1)22564  1)25365  1)29296  1)36704  1)38370  2)02300  2)06022  2)06066  2)07114  2)07184  2)07388  2)07502  2)08356  2)10171  2)11962  2)13581  2)14167  2)17502  2)19496  2)19973  2)21017  2)21856  2)21960  2)23691  2)25804  2)27846  2)28000  2)28665  2)29426  3)01033  3)08009  3)17693  3)17704  3)18885 | 1.28E-20  2.40E-27  3.09E-59  2.50E-08  8.53E-57  7.30E-56  2.55E-08  1.08E-27  0.0  2.78E-39  3.55E-39  5.27E-85  4.06E-43  0.0  0.00  9.54E-135  1.77E-133  3.52E-87  3.01E-27  2.03E-151  3.47E-66  9.76E-69  7.22E-130  0  4.78E-55  1.16E-170  0.00  6.52E-125  0.00  0.00  1.52E-62  8.28E-106  3.70E-74  8.61E-28  0.00  2.40E-134  2.33E-33  1.93E-143  1.31E-81  4.56E-109  4.02E-54  5.64E-55  1.91E-141  1.42E-139  1.27E-0  6.00E-08  2.00E-59  1.00E-57  5.00-130  6.00E-48 | XP_002404506  ACT09396  XP_002413220  XP_002415470  XP_002411203  XP_002408667  EFN65141  XP_002435695  XP_002408425  XP_002410449  XP_002401771  XP_002430445  XP_002431270  XP_002407641  XP_002409162  NP_001011573  XP_002410441  XP_002405695  XP_002406475  XP_002413279  XP_002413508  XP_002407136  AAM47017  XP_002413279  XP_002413220  XP_002411203  XP_002435695  XP_314741  XP_002407641  XP_002400087  XP_002404506  ACT09396  XP_969654  XP_002433650  XP_002408425  XP_002410441  XP_002406357  XP_002409162  XP_002406475  XP_002415471  XP_002413464  XP_002401771  XP_002408667  XP_002424050  XP_002436005  XP_002400088  XP_002409162  XP_002129091  CCA67993  XP_312021 | *Ixodes scapularis*  *Drosophila melanogaster*  *Ixodes scapularis*  *“ “*  *“ “*  *“ “*  *Campanotus floridanus*  *Ixodes scapularis*  *“ “*  *“ “*  *“ “*  *Pediculus humanus*  *“ “*  *Ixodes scapularis*  *“ “*  *Apis mellifera*  *Ixodes scapularis*  *“ “*  *“ “*  *“ “*  *“ “*  *“ “*  *Ixodes scapularis*  *Homarus americanus*  *Ixodes scapularis*  *“ “*  *“ “*  *“ “*  *Anopheles gambiae*  *Ixodes scapularis*  *“ “*  *“ “*  *Drosophila melanogaster*  *Tribolium castaneum*  *Ixodes scapularis*  *“ “*  *“ “*  *“ “*  *“ “*  *“ “*  *“ “*  *“ “*  *“ “*  *“ “*  *Pediculus humanus*  *Ixodes scapularis*  *“ “*  *Ciona intestinalis Drosophila melanogaster Anopheles gambiae* | Three types, nmda, ionotropic and metabotropic types found  7 transmembrane sweet-taste receptor of 3 GCPR |
| Octopamine | 399  622  280  928  704  673  472  1352  841  504  678  270  70 | 1)00252  1)16101  1)18983  1)24237  2)07909  2)07981  2)11022  2)12937  2)17391  2)27930  2)29991  3)05350  3)08847 | 2.84E-60  5.78E-67  7.50E-24  2.40E-129  2.04E-169  3.75E-92  2.61E-45  2.55E-174  6.48E-83  7.89E-74  2.64E-109  2.00E-36  Top of Form  7.00E-63 | XP_002411135  XP_002405020  XP_002401109  XP_002411130  XP_002408422  XP_002401109  XP_002416095  XP_002400568  XP_002408812  XP_002401109  XP_002411135  XP_002733926  XP_311113 | *Ixodes scapularis*  *“ “*  *“ “*  *“ “*  *“ “*  *“ “*  *“ “*  *“ “*  *“ “*  *“ “*  *“ “*  *Saccoglossus kowalesvkii*  *Anopheles gambiae* | 7 Tm superfamily Transmembrane (Olfactory – rhodopsin) receptor |
| Opioid growth factor | 529  1068 | 1)38340  2)22279 | 1.09E-26  3.35E-43 | XP_002591748  XP_001373266 | *Branchiostoma floridae*  *Monodelphis domestica* | Modulates neurotransmission growth , controls food intake |
| Serotonin | 235  296  1299  1139  1473 | 1)00587  1)18641  2)09185  2)09746  2)17069 | 7.03E-27  5.38E-43  1.77E-124  0.0  0.0 | XP_002404998  XP_002410573  XP_002406470  XP_002404998  XP_002405023 | *Ixodes scapularis*  *“ “*  *“ “*  *“ “*  *“ “* | Modulate many neurotransmitters and many hormones. |
| Pyrokinin-like | 916 | 2)08246 | 7.72E-86 | ACC99623 | *Dermacentor variabilis* | Matches *I. scapularis* untitled GPCR XM_002401136 |

**Gene Category**

**C. Other GPCR receptors**

(Neuropeptide signaling pathway):

**GO:0007218,**

| Pheromone/odorant receptor | 227  910  5737  729  165 | 1)00323  1)08895  2)06663  2)12038  3)13923 | 4.59E-34  2.01E-157  0.00  5.98E-04  1.00E-30 | XP_002415642  “  XP_002415642  XP_002943183  “ | *Ixodes scapularis*  *“ “*  *“ “*  *“ “*  *“ “* | | Type 1 periplasmic binding fold superfamily of GPCRs; includes 7 transmembrane sweet-taste receptor of 3 GPCRs. |
| --- | --- | --- | --- | --- | --- | --- | --- |
| Gustatory receptor | 228 | 1)18657 | 4.76E-15 | XP_001606656 | *Nasonia vitripennis* | |  |
| Other GPCRs unidentified | 223  203  224  211  609  1170  876  1522  979  527  288  1016  496  247  732  438  324  220  284  1202  206  234  369  469  304  436  375  736  614  308  334  3072  1925  1492  2092  2649  1645  1248  980  795  1043  1477  1339  598  683  622  1055  534  1977  438  470  416  1465  950  380  631  527  686  315  925  616  504  155 | 1)01033  1)01150  1)03505  1)03811  1)03639  1)06126  1)06884  1)07897  1)07190  1)09192  1)10114  1)10574  1)13116  1)13437  1)16712  1)19398  1)19585  1)19885  1)25524  1)28111  1)35509  1)40037  1)40323  1)40936  2)00243  2)01829  2)01938  2)01952  2)02080  2)02934  2)03182  2)05852  2)05959  2)06292  2)06310  2)06695  2)06888  2)07709  2)07965  2)08072  2)10093  2)10169  2)11256  2)11278  2)11367  2)11628  2)11986  2)12402  2)12998  2)14026  2)15940  2)17223  2)19545  2)20872  2)21108  2)22607  2)22929  2)24011  2)24187  2)27473  2)27492  2)27930  3)03060 | 5.26E-38  3.85E-25  1.26E-31  6.97E-19  6.59E-68  8.06E-179  9.81E-90  4.98E-89  7.73E-65  1.39E-90  4.14E-51  3.38E-135  1.39E-67  1.03E-38  1.21E-73  6.22E-44  1.20E-42  1.44E-35  1.30E-36  1.45E-138  8.29E-28  1.52E-37  2.07E-34  2.36E-06  9.57E-40  2.85E-52  7.88E-58  1.09E-61  4.75E-90  2.42E-51  1.98E-53  0.0  0.0  1.52E-130  1.96E-137  0.0  1.61E-152  0.0  2.57E-132  2.51E-94  2.10E-127  1.61E-124  0.0  4.16E-27  2.10E-13  2.48E-65  0.0  6.19E-89  0.0  4.09E-67  6.32E-07  6.22E-07  0.0  1.16E-142  1.43E-67  8.78E-98  1.48E-63  1.12E-46  3.23E-59  4.28E-134  4.52E-88  7.89E-74  6.00E-15 | XP_002403373  XP_002407612  XP_002401251  XP_002403880  XP_002402353  XP_002400964  XP_002400445  XP_002400785  XP_002409036  XP_002413122  XP_002409036  XP_002415939  XP_002413666  XP_002413122  XP_002431747  XP_002413383  XP_002401744  XP_002401850  XP_002413201  XP_002409856  XP_002400785  XP_002413201  XP_002413122  XP_002422806 XP_002407612  XP_002407991  XP_002409145  XP_002402366  XP_002399655  XP_002403373  XP_002407612  XP_002400445  XP_002413201  XP_002400785  XP_002409856  XP_321715  XP_002401850  XP_002415939  XP_002435684  XP_002400970  XP_002435623  XP_002408545  XP_002409036  XP_002400970  XP_002435623  XP_002433795  XP_002400964  XP_002416227  XP_002401251  XP_002409829  XP_001149617  XP_002410254  XP_002408737  XP_002403880  XP_002409399  XP_002414124  XP_002414124  XP_002413122  XP_002401744  XP_002399655  XP_002413383  XP_002401109  XP_001189214 | | *Ixodes scapularis*  *“ “*  *“ “*  *“ “*  *“ “*  *“ “*  *“ “*  *“ “*  *“ “*  *“ “*  *“ “*  *“ “*  *Pediculus humanus*  *Ixodes scapularis*  *“ “*  *“ “*  *“ “*  *“ “*  *“ “*  *“ “*  *“ “*  *“ “*  *“ “*    *“ “*  *“ “*  *“ “*  *“ “*  *“ “*  *“ “*  *“ “*  *“ “*  *“ “*  *“ “*  *“ “*  *“ “*  *Anopheles gambiae*  *Ixodes scapularis*  *“ “*  *“ “*  *“ “*  *“ “*  *“ “*  *“ “*  *“ “*  *“ “*  *“ “*  *“ “*  *“ “*  *“ “*  *“ “*  *“ “*  *“ “*  *“ “*  *“ “*  *“ “*  *“ “*  *“ “*  *“ “*  *“ “*  *“ “*  *“ “*  *S. purpuratus* | 7 transmembrane superfamily of GPCRs; members of the Rhodopsin family. Many are also olfactory receptors. |

**Gene Category**

**D. Hormone receptors/**

**Other/steroid receptors**

**(**GO: 0035076/GO:0003707/

GO: 0050810) GO:0050810

| Ecdysone nuclear receptor | | 2336  699  926  620 | | 2)10098  2)14526  2)20609  2)20681 | | 4.77E-79  9.06E-84  1.26E-93  7.58E-75 | | XP_970369  XP_002410972 XP_002427418  XP_002410049 | | *Tribolium castaneum*  *Ixodes scapularis*  *Pediculus humanus corporis*  *Ixodes scapularis* | | Regulates ecdysial process, molting, reproduction |
| --- | --- | --- | --- | --- | --- | --- | --- | --- | --- | --- | --- | --- |
| Juvenile hormone esterase binding protein | | 454 | | 2)17587 | | 9.60E-32 | | XP_002411729 | | *Ixodes scapularis* | | Inhibits JH esterase expression |
| Other steroid receptors | | 2000  192  474  1533  1313  518  118  966  247  24 | | 1)28003  1)40161  1)40333  2)06453  2)08266  2)23498  3)00673  3)04321  3)04948  3)17587 | | 0.0  8.12E-31  4.47E-82  0.0  8.00E-141  5.99E-83  8.00E-29  0.0  0.0  0.0 | | XP_002412008  XP_002403573  XP_002412008  “  XP_392402  XP_002412008  “  XP_002409132  XP_002407037  “ | | *Ixodes scapularis*  *“ “*  *“ “*  *“ “*  *“ “*    *“ “*  *Apis mellifera*  *“ “*  *Ixodes scapularis* | | [intracellular mostly nuclear receptors](http://en.wikipedia.org/wiki/Intracellular_receptor) initiate [signal transduction](http://en.wikipedia.org/wiki/Signal_transduction); receptors for thyroid hormone, retinoids, cholesterol by-products, lipids and heme |
| Steroidogenic activating/regulatory protein | | 1174  1117  271 | | 1)06160  2)08212  3)13885 | | 1.10E-151  1.59E-176  1.00E-114 | | XP_002404588  XP_002413856  XP_002413856 | | *Ixodes scapularis*  *“ “*  *“ “* | |  |
| Steroid reductase/dehydrogenase | 437  1346  1208  658  404  688  355  284 | | 1)10944  1)27847  1)28853  1)28963  1)33404  2)18510  2)30768  3)11886 | | 4.05E-43  1.80E-180  1.90E-154  1.97E-87  3.35E-69  1.41E-81  1.54E-37  2.00E-154 | | XP_002411079  XP_002434360  XP_002410985  XP_002407062  XP_002434323  XP_002411079  XP_002410985 XP_002410985 | | *Ixodes scapularis*  *“ “*  *“ “*  *“ “*  *“ “*  *“ “*  *“ “*  *“ “* | | Steroid biosynthesis/metabolism | |

**Gene Category**

**E. Reproduction/development**

**related** (GO: 0048609)

| Spermatogenesis-associated protein | 689  656  779  305  227  2437  1216  82  104  127 | 1)00124  1)23137  1)25978  1)33467  1)34946  2)07257  2)27857  2)03284  3)17213  3)19582 | 6.24E-66  2.36E-30  2.68E-146  9.37E-56  9.42E-78  0.0  4.00E-30  1.00E-61  3.00E-17  8.00E-41 | XP_002416007  XP_424288  XP_002399229  XP_002399229  XP_002416007  XP_002399229  XP_002399229  XP_002399229  XP_002752480  XP_540960 | *Ixodes scapularis*  *Gallus gallus*  *Ixodes scapularis*  *“ “*  *“ “*  *“ “*  *“ “*  *“ “*  *“ “*  *Canis familiaris* | Zinc-finger containing PUB domain as in p97 adaptor proteins such as PNGase and others |
| --- | --- | --- | --- | --- | --- | --- |
| Major sperm | 1518  1056 | 1)37011  2)22870 | 2.75E-71  1.24E-74 | XP_002412020  XP_002412020 | *Ixodes scapularis*  *Ixodes scapularis* | Sec14p-like lipid-binding domain. |
| n-acetyl-spermine/ spermidine oxidase | 1097  207  1707  1707  390 | 1)06904  1)16980  2)27608  2)03024  3)13480 | 6.93E-137  4.23E-24  1.30E-136  2.72E-94  8.00E-115 | XP_002433393  XP_002433994  XP_002433993  X_002433994  XP_002433994 | *Ixodes scapularis*  *“ “*  *“ “*  *“ “*  *“ “* |  |
| Spermidine synthase | 739  195 | 2)08441  3)13196 | 2.96E-59  3.00E-99 | XP_002401286  DAA34588 | *Ixodes scapularis*  *Amblyomma variegatum* | S-adenosylmethionine-dependent methyl transferases superfamily |
| Epididymal secretory protein | 594  398 | 1)22168  1)32263 | 1.61E-84  6.60E-49 | XP_002435260  XP_002400803 | *Ixodes scapularis*  *“ “* |  |

**Gene Category**

**F. Iron transport protein/ferritin**

(GO:0070288)

| Ferritin | 831  404  537  650  172 | 1)06662  2)22249  2)23131  2)29208  3)00091 | 1.09E-103  3.00E-49  2.68E-63  1.30E-91  2.00E-87 | ACJ70653  ACJ70653  NP_001095158  XP_002721094  AAL75582 | *Ixodes ricinus*  *“ “*  *Oryctolagus cuniculus*  *“ “*  *Dermacentor variabilis* | Protection against toxic levels of iron. Takes up ferrous iron, converts to ferric hydroxide and stores ready for use. |
| --- | --- | --- | --- | --- | --- | --- |

**Gene Category**

**G. Immune peptides** (GO:006955)

| Preprodefensin | 322  395  317  325  67  74  29 | 1)18941  1)37527  2)12814  2)22230  3)06916  3)13785  3)15267 | 2.86E-36  1.26E-31  2.94E-149  1.36E-17  8.00E-16  4.00E-29  7.00E-25 | XP_002401521  ABC88432  XP_002401477  ABC88432  AAO18363  ACJ04433  ABW08118 | *Ixodes scapularis*  *Ixodes ricinus*  *Ixodes scapularis*  *Ixodes ricinus*  *D. variabilis*  *D. marginatus*  *H. longicornis* | Antimicrobial peptide |
| --- | --- | --- | --- | --- | --- | --- |
| Hemolectin | 617  318  977  429  278  399  1034  1046  355  433  646  540  496  425  1024 | 1)12994  1)17896  1)22726  1)26732  1)26835  1)34361  2)08910  2)21544  2)21996  2)22360  2)22904  2)24365  2)27864  2)28827  3)03872 | 1.36E-12  1.87E-27  9.72E-60  9.85E-05  9.13E-11  1.97E-77  1.28E-69  0.0  7.86E-50  2.23E-04  6.41E-131  6.88E-99  1.40E-06  5.11E-65  9.00E-146 | XP_395067  XP_002415168  ABO09954  XP_002738041  EFA10333  XP_002409356  EFA10333  XP_002436012  XP_002409356  XP_002430354  XP_002436012  XP_002409356  XP_395067  XP_002409356  XP_002409356 | *A. mellifera*  *I. scapularis*  *I. ricinus*  *S. kowalevskii*  *T. castaneum*  *I. scapularis*  *Triboleum castaneum*  *I. scapularis “*  *I. scapularis*  *Pediculus humanus*  *I. scapularis*  *I. scapularis*  *Apis mellifera*  *I. scapularis*  *“ “* | Lectin; coagulation factor domain, Cell surface-attached carbohydrate-binding domain, |
| Ixoderin | 217  757  334  977  250  238  252  392  940  378  522  163 | 1)04768  1)11042  1)13569  1)22726  1)31404  1)33155  1)39274  2)21216  2)25100  2)26605  2)29757  3)04637 | 1.49E-13  5.07E-46  7.90E-55  9.72E-60  1.30E-41  5.05E-17  7.68E-21  6.40E-20  2.85E-101  6.91E-46  4.36E-68  2.00E-59 | XP_002403569  XP_002399618  XP_002411719  ABO09954  XP_002415669  AAQ93650  XP_002415352  XP_002412814  XP_002401248  ABO09954  ABO09955  XP_002411719 | *Ixodes scapularis*  *“ “*  *“ “*  *Ixodes ricinus*  *Ixodes scapularis*  *Ixodes ricinus*  *“ “*  *Ixodes ricinus*  *Ixodes scapularis*  *Ixodes ricinus*  *Ixodes ricinus*  *Ixodes scapularis* | Fibrolectin Fred Domain |
| Galectin | 1268  613  665  709  303  304 | 1)25467  1)29659  1)37300  2)08606  3)10685  3)17581 | 3.27E-112  1.05E-113  5.96E-47  4.26E-28  5.00E-105  1.00E-109 | XP_002410170  XP_002403321 “  XP_002411050  XP_002411050  XP_002410170  XP_002403321 | *Ixodes scapularis*  *“ “*  *“ “*  *“ “*  *Ixodes scapularis*  *“ “* | Lectin Fibrolectin Fred Domain |
| Peptidoglycan recognition protein | 452  355  855  453 | 1)37155  2)02955  2)14247  2)14638 | 4.39E-45  1.88e-27  6.12e-84  5.99E-50 | XP_002413091  XP_002411776  XP_002433689  AAY66906 | *Ixodes scapularis*  *“ “*  *“ “*  *“ “* | PGRP |
| Microplusin | 483 | 1)13802 | 2.86E-52 | AAY66495 | *Ixodes scapularis* | Antimicrobial peptide |
| Alpha-macroglobulin | 204  5054  1773  2453  353  744  492 | 1)19003  1)21451  1)21848  2)06871  2)13396  2)21734  2)22914 | 2.32.E-30  0.0  4.56E-161  2.49E-86  1.50E-24  1.31-54  2.76E-87 | XP_002404137  XP_002405338  XP_002401146  XP_002405336  XP_002410474  XP_002405338  XP_002416333 | *Ixodes scapularis*  *“ “*  *“ “*  *“ “*  *“ “*  *“ “*  *“ “* | Antimicrobial peptide |
| Subolesin (4D8) possible immune-related | 2681  1196  165 | 1)21720  2)07466  3)04031 | E 0.0  6.78D-27  8.00E-82 | XP_002411799  ABA62325  ADA85876 | *Ixodes scapularis*  *Ixodes ricinus*  *R. microplus* | Regulates expression of innate immune response genes. |

**Gene Category**

**H. Oxidative Stress**

(GO: 0055114)

| Oxidative stress induced-growth | 1104  900  613  1735 | 1)09482  1)25612  1)29657  2)06711 | 0.0  1.21E-106  7.60E-88  0.0 | XP_002403487  XP_002433496  XP_002409819  XP_002403487 | *Ixodes scapularis*  *“ “*  *“ “*  *“ “* |  |
| --- | --- | --- | --- | --- | --- | --- |
| Oxireductases | 487  663  1415  977  593  396  377  345  1049  1874  2112  339  343  1725  505  311 | 1)07855  1)08866  1)22371  1)28349  1)30864  1)031293  1)32656  1)35120  2)03131  2)06616  2)06934  2)26988  2)27249  2)27426  2)30294  3)03988 | 1.78E-62  3.94E-83  0.0  1.79E-146  8.65E-99  8.90E-54  1.12E-40  1.02E-49  9.09E-163  9.65E-165  1.50E-177  1.01E-57  4.24E-43  0.0  1.30E-68  7.00E-119 | AAQ21387  XP_002434769  XP_002405626  XP_002413990  XP_002409125  ADN23574  XP_002410402  XP_002416195  XP_002434997  XP_002408150  XP_002411785  XP_002405626  XP_002415709  XP_002405450 XP_002435000 XP_002405626 | *Ixodes ricinus*  *Ixodes scapularis*  *“ “*  *“ “*  *“ “*  *Hyalomma marginatum ruf.*  *Ixodes scapularis*  *“ “*  *“ “*  *“ “*  *“ “*  *“ “*  *“ “*  *“ “*  *“ "*  *“ “* | Catalyzes redox reactions |
| Thioredoxin | 669  850  864  201  642  1002  1189  592  364  529  1160  676  713  1778  1073  379  402  578  380  130  285  215  198  107  60  61  142  60  237  200  67 | 1)07192  1)22380  1)23649  1)27237  1)37031  1)37339  2)08502  2)08618  2)13339  2)14079  2)17148  2)18052  2)18257  2)19640  2)23608  2)23631  2)24274  2)24544  2)25718  3)02930  3)04021  3)04425  3)04735  3)05639  3)07357  3)08475  3)09421  3)10734  3)13320  3)13498  3)14744 | 1.91E-125  70E-158  8.24E-142  6.82E-14  1.02E-32  3.84E-99  0.0  7.94E-108  4.28E-32  1.38E-69  1.07E-146  2.75E-50  1.47E-44  1.96E-151  3.37E-152  5.13E-33  1.93E-72  1.18E-60  2.28E-65  4.00E-57  1.00E-100  2.00E-55  4.00E-111  9.00E-46  6.00E-28  3.00E-08  2.00E-76  6.00E-21  3.00E-120  4.00E-145  2.00E-22 | XP_002408098  XP_002404402  XP_002415327  XP_002436274  ACF35500  XP_001608075  XP_002408098 XP_002435441  XP_002404157  XP_002434886  XP_002436084  XP_780032  XP_002435776  XP_002406290  XP_002404402  ACF35500 XP_002415327  XP_001608075  XP_002435441 XP_002434886 XP_001608075 NP_001072538 ACX54025  DAA34571 XP_002406290  XP_002129555  NP_006692  AAY66989  AAY66580  XP_002435441  DAA34100 | *Ixodes scapularis*  *“ “*  *“ “*  *“ “*  *Dermacentor variabilis*  *Nasonia vitripennis*  *Ixodes scapularis*  *“ “*  *“ “*  *“ “*  *“ “*  *S. purpuratus*  *Ixodes scapularis*  *“ “*  *“ “*  *Dermacentor variabilis*  *Ixodes scapularis*  *Nasonia vitripennis*  *Ixodes scapularis*  *Ixodes scapularis*  *Nasonia vitripennis*  *Xenopus tropicalis*  *Rhipicephalus sanguineus*  *Amblyomma variegatum*  *Ixodes scapularis*  *Ciona intestinalis*  *Homo sapiens*  *Ixodes scapularis*  *“ “*  *“ “*  *Amblyomma variegatum* | Antioxidants facilitating reduction of other proteins. Thioredoxins are thiol-specific antioxidants; peroxidase activity by reducing hydrogen peroxide, peroxynitrite, and organic hydroperoxides. |
| Superoxide dismutase | 432  330  749  1045  321  491  1175  463  149  154  48  75  161  89 | 1)03357  1)34959  1)36917  1)36826  2)15984  2)17348  2)19258  2)25814  3)06113  3)06308  3)07383  3)07636  3)13424  3)13947 | 1.56E-10  1.50E-45  1.16E-111  3.74E-52  1.12E-11  9.90E-69  4.97E-83  2.22E-84  1.00E-44  2.00E-79  2.00E-06  6.00E-42  1.00E-99  5.00E-35 | XP_002596245  XP_002405905  XP_002403511  XP_002408837  XP_314137  XP_002414489  XP_002413277  XP_002414082 ABR27983  ACF35508  XP_002433575 DAA34214 XP_002403511  Swiss-Prot: P11418 | *Branchiostoma floridae*  *Ixodes scapularis*  *“ “*  *“ “*  *“ “*  *“ “*  *“ “*  *“ “*  *Triatoma infestans*  *Dermacentor variabilis*  *Ixodes scapularis*  *Amblyomma variegatum*  *Ixodes scapularis*  “ “ | Antioxidant; main [reactive oxygen species](http://en.wikipedia.org/wiki/Reactive_oxygen_species)  In eukaryotic cells |
| Glutathione S-transferase | 202  287  252  956  849  514  430  683  861  370  218  249  329  517  218  775  316  763  305  762  200  1318  377  579  592  219  211  220  542  570  657  447  316  465  498  614  769  325  1293  893  1135  670  370  724  806  381  827  722  416  489  445  546  624  1313  432  300  118  220  229  207  216  118  121  106  82  159  170  105  104  157  213  223  191  134 | 1)01647  1)01914  1)05130  1)07368  1)07915  1)08127  1)11816  1)12680  1)13397  1)13656  1)14932  1)18122  1)18445  1)23043  1)24064  1)24140  1)24426  1)24443  1)25289  1)25301  1)27140  1)28377  1)30382  1)30489  1)32298  1)34020  1)36097  1)36262  1)37221  1)38367  1)38835  1)40135  2)00145  2)02343  2)03005  2)03329  2)03448  2)04683  2)08435  2)08937  2)09076  2)09439  2)11216  2)16270  2)17544  2)17639  2)18026  2)19299  2)21262  2)22551  2)24314  2)25132  2)28284  2)28883  2)29306  2)29358  3)01361  3)04144  3)04351  3)04654  3)04829  3)04880  3)06287  3)06541  3)09969  3)11683  3)14523  3)15733  3)18073  3)18075  3)18099  3)18242  3)18256  3)18513 | 1.05E-30  6.44E-36  3.91E-41  3.60E-128  3.39E-132  4.12E-73  8.71E-78  3.36E-56  1.48E-82  9.84E-61  1.44E-24  1.35E-30  5.37E-19  1.04E-79  1.26E-36  1.80E-59  7.80E-114  2.42E-128  9.12E-27  2.79E-92  1.86E-27  0.0  5.07E-25  7.33E-63  4.22E-53  3.93E-33  3.82E-33  7.92E-34  3.19E-40  1.76E-98  1.74E-67  1.78E-70  1.05E-33  8.19E-47  2.09E-87  5.41E-102  2.66E-146  8.70E-57  6.06E-109  9.28E-90  9.64E-90  9.36E-120  1.66E-47  2.54E-132  4.91E-53  9.88E-61  2.91E-80  1.50E-76  3.97E-54  8.15E-47  4.19E-59  8.32E-39  1.27E-109  8.08E-117  9.87E-53  5.29E-30  2.00E-34  6.00E-99  5.00E-80  6.00E-59  8.00E-72  2.00E-92  3.00E-101  3.00E-33  8.00E-30  2.00E-42  2.00E-56  2.00E-36  5.00E-47  3.00E-75  1.00E-84  5.00E-113  1.00E-112  2.00E-50 | XP_002412446  XP_002436292  XP_002434206  XP_002402685  XP_002400733  XP_002434207  XP_002416663  XP_002402865  XP_002436291  XP_002416387  XP_002399298  XP_002408098  EDL02925  XP_002410551  XP_002407710  XP_002434207  XP_002401397  XP_002401423  XP_002402637  XP_002410551  XP_002406052  XP_002401856  XP_002401749  XP_002414188  XP_002400734  XP_002407711  XP_002436292  XP_002412446  XP_002435664  XP_002436290  XP_002406485  XP_002405467  [XP_002400727](http://www.ncbi.nlm.nih.gov/gquery/?term=XP_002400727)  XP_00240263  XP_002435663  XP_002414672  XP_002416663  XP_002434206  XP_002406052  XP_002405750  XP_002407711  XP_002401423  XP_002436292  XP_002400733  XP_002402865  XP_002407710  XP_002410641  XP_002400734  XP_002436292  XP_002434207  XP_002400351  AAT92165 XP_002406485  XP_002412446  XP_002400734  XP_002402685 XP_002406485  XP_002402685  XP_002434207  XP_002416387  XP_002436292  XP_002400734  XP_002406052  XP_004213127  XP_002436292  XP_002436291  XP_002412446 XP_002435664  DAA34299  ACF35506  XP_2401423  ACF35539  ABB46494  XP_2416387 | *Ixodes scapularis*  *“ “*  *“ “*  *“ “*  *“ “*  *“ “*  *“ “*  *“ “*  *“ “*  *“ “*  *“ “*  *“ “*  *Mus musculus*  *Ixodes scapularis*  *“ “*  *“ “*  *“ “*  *“ “*  *“ “*  *“ “*  *“ “*  *“ “*  *“ “*  *“ “*  *“ “*  *“ “*  *“ “*  *“ “*  *“ “*  *“ “*  *“ “*  *“ “*  *“ “*  *“ “*  *“ “*  *“ “*  *“ “*  *“ “*  *“ “*  *“ “*  *“ “*  *“ “*  *“ “*  *Ixodes scapularis*  *“ “*  *“ “*  *“ “*  *“ “*  *“ “*  *“ “*  *“ “*  *Ixodes pacificus*  *Ixodes scapularis*  *“ “*  *“ “*  *“ “*  *“ “*  *“ “*  *“ “*  *“ “*  *“ “*  *“ “*  *“ “*  *“ “*  *Ixodes scapularis*  *“ “*  *Ixodes scapularis*  *“ “*  *Amblyomma variegatum*  *Dermacentor variabilis*  *Ixodes scapularis*  *Dermacentor variabilis*  *“ “*  *Ixodes scapularis* | Cellular detoxification by catalyzing the conjugation of glutathione (GSH) detoxifying environmental toxins and products of oxidative stress. GSTs also show GSH peroxidase activity and are involved in synthesis of prostaglandins and leukotrienes. |

**Gene Category**

**I. Environmental Stress** (**GO006950**)

| Heat shock 20 | 363  658  221  565  194  185  177  179  176 | 1)25836  1)30680  1)33285  2)23004  3)00223  3)04174  3)07243  3)10471  3)10513 | 1.18E-42  8.67E-68  1.50E-24  6.21E-72  1.00E-79  1.00E-79  2.00E-51  8.00E-51  2.00E-97 | XP_002414523  XP_002405513  XP_002416542  XP_002409819  XP_002409748  XP_002405513  AAO92281  XP_002415502  AAO92281 | *Ixodes scapulari*  *Ixodes pacificus*  *“ “*  *“ “*  *“ “*  *“ “*  *“ “*  *Ixodes scapularis*  *Dermacentor variabilis* | Small heat shock protein; chaperone;  Prevent aggregation, unfolding, etc. |
| --- | --- | --- | --- | --- | --- | --- |
| Heat shock 40 | 477 | 2)22738 | 2.74E-87 | XP_002410908 | *Ixodes scapularis* | Small heart shock protein; chaperone |
| Heat shock 70 | 269  236  362  468  385  454  407  203  2563  2711  2429  1194  1983  1525  271  310  346  293  2252  1516  1040  662  220  651  1222  1698  1306  701  1397  592  849  472  1058  626  989  1208  219  470  612  282  129  241 | 1)00144  1)00222  1)01222  1)03449  1)11196  1)11966  1)12784  1)15954  1)21439  1)21540  1)21837  1)22563  1)22862  1)24469  1)30593  1)32360  1)32778  1)35727  1)37042  1)37053  1)38114  1)38963  1)41015  2)02433  2)05902  2)06694  2)06774  2)09890  2)13587  2)14315  2)14559  2)14581  2)14940  2)18252  2)19308  2)22461  3)01145  3)04323  3)05298  3)06350  3)06857  3)11331 | 1.97E-45  5.57E-24  4.35E-37  1.37E-38  4.94E-65  5.09E-78  4.35E-61  1.46E-32  0.0  0.0  0.0  1.41E-93  0.0  2.86E-169  9.80E-21  1.64E-52  2.13E-47  1.25E-31  0.0  0.0  2.78E-116  3.18E-109  7.16E-35  9.06E-109  1.71E-125  0.0  3.91E-172  1.17E-123  0.0  1.36E-91  5.00E-131  1.28E-84  2.69E-154  2.53E-33  1.80E-149  7.46E-98  7.00E-103  0.0  0.0  8.00E-45  1.00E-118  4.00E-116 | XP_002406287  XP_002596379  XP_002435150  XP_002412200  XP_002410673  XP_002435736  ABQ12812  XP_002406287  XP_002402562  XP_002401154  XP_002433656  XP_002408076  XP_002415926  XP_311405  XP_002433721  XP_002415063  XP_002415502  XP_002409711  XP_002407132  XP_002407411  XP_002733893  XP_002409748  XP_002407411  XP_002400940  XP_002406287  XP_002404675  XP_002412200  XP_002415926  XP_002435736  XP_002406560  XP_002407411  XP_002401154  XP_002435150  XP_002402562  AAC41542  XP_002410673  XP_002414808  XP_002407411  XP_002407132  XP_002404675  XP_002401154  XP_002400940 | *Ixodes scapularis*  *Branchiostoma floridae*  *Ixodes scapularis*    *“ “*    *“ “*  *“ “*  *Ixodes scapularis*  *“ “*  *“ “*  *“ “*  *“ “*  *“ “*  *“ “*  *“ “*  *Anopheles gambiae*  *Ixodes scapularis*  *Ixodes scapularis*  *“ “*  *“ “*  *“ “*  *“ “*  *“ “*  *S. kowalevskii*  *Ixodes scapularis*  *“ “*    *“ “*  *“ “*  *“ “*  *“ “*  *“ “*  *“ “*  *“ “*  *“ “*  *“ “*  *Anopheles albimanus*  *Ixodes scapularis*  *“*  *“ “*  *“ “* | Heat shock proteins regulate translation, folding, unfolding, translocation, and degradation. |
| Heat shock 90 | 1095  1013  524  304  457  308  410  72  612  611  240  270  733  335 | 1)23174  2)00520  2)01655  2)02501  2)17584  2)23452  2)26141  3)00257  3)03789  3)03797  3)04045  3)04745  3)05485  3)16334 | 8.85E-100  1.13E-130  4.04E-90  1.81E-30  8.55E-73  3.87E-49  2.35E-62  3.00E-36  0.0  0.0  2.00E-66  7.00E-57  0.0  6.00E-134 | XP_002402135  XP_002400940  XP_002711276  EAW97723  XP_002711276  XP_002415167  XP_002403553  XP_002414808 XP_002402562  ACA84007  XP_002408076  XP_002403553  BAI23206  XP_001654758 | *Ixodes scapularis*  *“ “*  *“ “*  *Homo sapiens*  *Oryctolagus cuniculus*  *Ixodes scapularis*  *“ “*  *“ “*  *“ “*  *Haemaphysalis longicornis*  *Ixodes scapularis*  *“ “*  *Coturnix japonica*  *Aedes aegypti* |  |

**Gene Category**

**J. Cuticle synthesis –digestion**

| Chitin synthase | 206  209  280  412  363  1720  416  625 | 1)04157  1)05778  1)27810  2)01254  2)03775  2)08141  2)21348  2)21795 | 3.59E-31  5.91E-26  9.08E-19  2.79E-15  9.21E-35  0.0  3.46E-74  1.10E-28 | XP_002402530  XP_002405231  ABX56676  XP_002423597  XP_002405234  XP_002402530  XP_002405231  XP_320185 | *Ixodes scapularis*  *“ “*  *Mamesta brassicae*  *Pediculus humanus corporis Ixodes scapularis*  *“ “*  *“ “*  *Anopheles gambiae* | Stimulate cuticle synthesis |
| --- | --- | --- | --- | --- | --- | --- |
| Chitin synthase activator | 225 | 1)18107 | 1.83E-35 | XP_002410938 | *Ixodes scapularis* | Cuticle synthesis during feeding |
| Chitinase | 219  1091  121  297  105  277  222 | 1)10594  2)00480  3)01563  3)05361  3)06229  3)13610  3)13656 | 2.71E-34  6.13E-16  7.00E-33  3.00E-123  6.00E-45  0.0  6.00E-90 | XP_002413490  XP_002399542  XP_001862401  XP_002407799  XP_002407801  ACX33152  XP_002407799 | *Ixodes scapularis*  *“ “*  *Culex quinquefasicatus*  *Ixodes scapularis*  *“ “*  *Rhipicephalus sanguineus*  *Ixodes scapularis* | Digestion/degradation chitin |

**^a^** GO terms are Bioprocesses definitions from the Gene Ontology Consortium ([www.geneontology.org](http://www.geneontology.org)).

^b^ If multiple contigs matched the same GenBank accession number, only contigs with the highest e-values matching that accession number were included.

^c^ Only the matching GenBank accession number with the highest e-value (top hit) are listed.

.
